# An improved protein extraction method applied to cotton leaves is compatible with 2-DE and LC-MS

**DOI:** 10.1186/s12864-019-5658-5

**Published:** 2019-04-11

**Authors:** Xiang Jin, Liping Zhu, Chengcheng Tao, Quanliang Xie, Xinyang Xu, Lili Chang, Yanhua Tan, Guohua Ding, Hongbin Li, Xuchu Wang

**Affiliations:** 1 Ministry of Education Key Laboratory for Ecology of Tropical Islands, College of Life Sciences, Hainan Normal University, Haikou, 571158 China; 2 College of Life Sciences, Key Laboratory of Xinjiang Phytomedicine Resource and Utilization of Ministry of Education, Shihezi University, Shihezi, 832003 China; 3 Institute of Tropical Biosciences and Biotechnology, Chinese Academy of Tropical Agricultural Sciences, Haikou, 571101 Hainan China

**Keywords:** Cotton leaf, Protein extraction method, Proteomics, Polysaccharide and polyphenol, Two-dimensional electrophoresis, High-throughput LC-MS/MS

## Abstract

**Background:**

Two-dimensional electrophoresis (2-DE) and liquid chromatography-tandem mass spectrometry (LC-MS/MS) are widely used in plant proteomics research. However, these two techniques cannot be simultaneously satisfied by traditional protein extraction methods when investigate cotton leaf proteome.

**Results:**

Here, we evaluated the efficiency of three different protein extraction methods for 2-DE and LC-MS/MS analyses of total proteins obtained from cotton leaves. The protein yield of the borax/PVPP/phenol (BPP) method (0.14%) was significantly lower than the yields of the trichloroacetic acid/acetone (TCA) precipitation method (1.42%) and optimized TCA combined with BPP (TCA-B) method (0.47%). The BPP method was failed to get a clear 2-DE electrophoretogram. Fifty pairs of protein spots were randomly selected from the 2-DE gels of TCA- and TCA-B-extracted proteins for identification by MALDI TOF/TOF, and the results of 42 pairs were consistent. High-throughput proteomic analysis showed that 6339, 9282 and 9697 unique proteins were identified from the total cotton leaf proteins extracted by the TCA, BPP and TCA-B methods, respectively. Gene Ontology (GO) analysis revealed that the proteins specifically identified by TCA method were primarily distributed in the plasma membrane, while BPP and TCA-B methods specific proteins distributed in the cytosol, indicating the sub-cellular preference of different protein extraction methods. Further, ATP-dependent zinc metalloprotease FTSH 8 could be observed in the 2-DE gels of TCA and TCA-B methods, and could only be detected in the LC-MS/MS results of the BPP and TCA-B methods, showing that TCA-B method might be the optimized choice for both 2-DE and LC-MS/MS.

**Conclusion:**

Our data provided an improved TCA-B method for protein extraction that is compatible with 2-DE and LC-MS/MS for cotton leaves and similar plant tissues which is rich in polysaccharides and polyphenols.

**Electronic supplementary material:**

The online version of this article (10.1186/s12864-019-5658-5) contains supplementary material, which is available to authorized users.

## Background

Proteomics has been developed as an important approach for studying plant functional genomics [1], which bridges the gap from transcriptome to metabolome [2] and can be used to detect the post-transcriptional modification [3, 4]. Numerous techniques, such as two-dimensional electrophoresis (2-DE), differential in-gel electrophoresis (DIGE), matrix-assisted laser desorption/ionization time-of-flight/time-of-flight mass spectrometry (MALDI-TOF/TOF MS), label-free quantification and isobaric tags for relative and absolute quantitation (iTRAQ) have been used extensively to study the global protein accumulation level in plant tissues [2, 5–9]. Compared with gel-based techniques, gel-free techniques (label-free and iTRAQ) are characterized by their high sensitivity and ability to identify highly acidic/alkaline or hydrophobic proteins [10]. Still, gel-based proteomics techniques are valuable for the visualized protein gels, protein spots abundance and information of protein isoforms coding by the same transcript, which could not be achieved by gel-free techniques [11, 12].

Cotton (*Gossypium hirsutum* L.) is an important industrial crop that is worldwide cultured for the production of textile fibres and cottonseed oil [13]. Recently, gel-free high-throughput proteomic techniques have identified a much higher number of proteins in cotton tissues than before. Using iTRAQ, we identified 2729 differential abundant proteins in ovules from Xuzhou 142 and a fuzzless-lintless mutant (*fl*) cotton at the day of anthesis, indicating that very-long-chain fatty acids play an important role in cotton fibre initiation [14]. A comparative proteomics study of cold stress in upland cotton leaves identified 7388 proteins, 443 of which were significantly differentially expressed. Further analysis revealed that cold stress was closely associated with cotton leaf senescence [15]. In addition, gel-based comparative proteomic analysis of different stages of developmental initiation and somatic embryogenesis found 149 differentialy abundant proteins, indicating that stress responses might regulate the balance of reactive oxygen species in cotton through interaction with auxin during somatic embryogenesis [16]. Also, we identified three ovule specific protein isoforms of 70-kD heat shock protein (HSP70) using 2-DE technique. Further study showed that the site-specific truncation of HSP70 might be involved in cotton fibre development [17].

The protein extraction methods used for processing plant samples may affect subsequent experimental results [18]. With regard to low-abundance proteins in rubber latex, we developed an improved protein extraction method for proteomics studies on rubber particles [19]. To study the proteins in the leaves and flowers of pigeon pea (*Cajanus cajan*), phenol extraction was found to work better in concert with 2-DE compared with the TCA method [20]. Regarding the methods used for extracting proteins from maize leaves, phenol-extracted protein functions better with 2-DE analysis, and the TCA method achieves a higher protein yield [21]. For the Australian seagrass *Zostera muelleri* and *Posidonia australis*, the borax/PVPP/phenol (BPP) method of protein extraction works better for 2-DE analysis [22]. It is necessary to evaluate and improve the effects of different protein extraction methods on the results of proteomic experiments with regards to specific plant tissues [23].

In our previous study, we found that the BPP method failed to produce high-quality 2-DE maps for cotton leaves; thus, we used the TCA method to extract total proteins for this experiment [24]. However, our subsequent study showed that the LC-MS/MS results obtained using the TCA-extracted proteins from cotton leaves were not qualified. Therefore, in this study, we evaluated the effects of the BPP, TCA and the improved TCA-combined BPP (TCA-B) protein extraction methods for 2-DE and LC-MS/MS. Our results showed that the BPP method achieved a significantly lower protein yield than the TCA and TCA-B methods. Gene ontology (GO) analysis and identification of representative proteins showed that for cotton leaves, the improved TCA-B method is compatible with both 2-DE and LC-MS/MS analyses of polysaccharide- and polyphenol-rich plant tissue, especially cotton leaves.

## Results

### Procedures and protein yields of the three different extraction methods

A schematic diagram of the experimental design for evaluating the three different extraction methods are shown (Additional file 1a). The improved TCA-B method involved an additional phenol extraction of the TCA-extracted proteins, avoiding the direct contact between the phenol and the plant tissues. Detailed protocols for the experimental methods are available in Additional file 1b. The concentrations of the proteins extracted by three different methods were 2.49 μg/μl (BPP), 9.78 μg/μl (TCA) and 7.23 μg/μl (TCA-B). The corresponding protein yields were 1371.18 μg (0.14%) for the BPP method, 14,177.69 μg (1.42%) for the TCA method and 4698.26 μg (0.47%) for TCA-B method. These results showed that the protein yield of the BPP method ranked the lowest, while the protein yield of the TCA method was the highest (Additional file 2).

### 2-DE analysis of proteins extracted by the three different methods

**Fig. 1:**
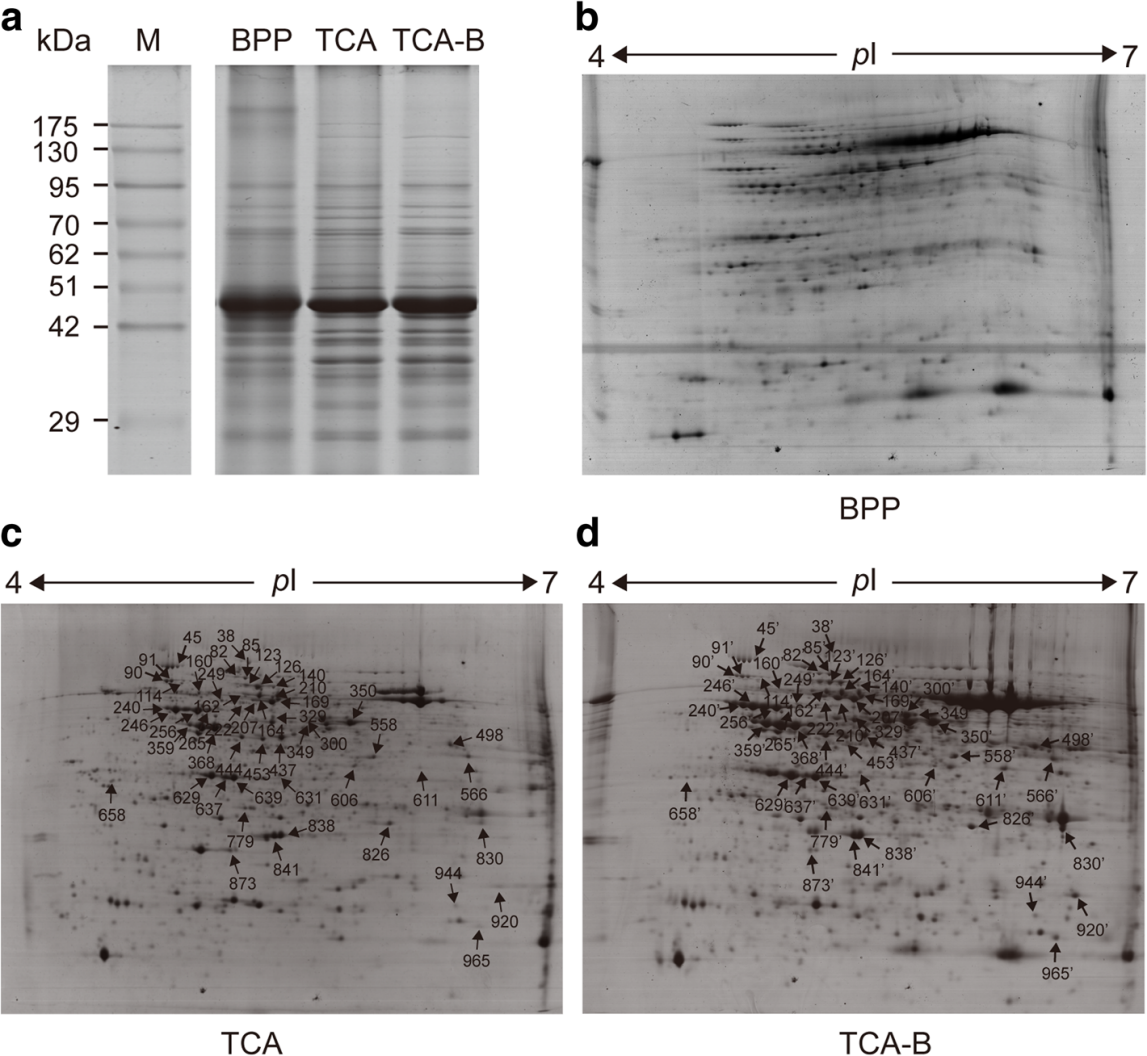
Representative SDS-PAGE (**a**) and 2-DE gels of the total proteins extracted by three methods. In the 2-DE gels, the isoelectric point ranged from 4 to 7. Fifty pairs of protein spots were randomly selected from the TCA (**c**) and TCA-B (**d**) gels for MALDI TOF/TOF (indicated by arrows and numbers). Due to the failure to obtain a clear 2-DE map, no protein spots were selected from the BPP gel (**b**) for MS identification

The quality of proteins extracted by the three methods was assessed by SDS-PAGE and 2-DE. The results showed that the BPP method failed to produce either clear electrophoretic bands (Fig. 1a) or clear protein spots (Fig. 1b). By contrast, both the TCA and TCA-B methods supported the acquisition of clear electrophoretic bands (Fig. 1a) and high-quality two-dimensional electropherograms (Fig. 1c, d). To evaluate the effect of the TCA-B method on the 2-DE results, we randomly selected 50 pairs of protein spots from the 2-DE gels of the TCA and TCA-B methods for mass spectrometric identification by MALDI TOF/TOF MS (arrows in Fig. 1c, d). A total of 42 pairs of protein spots showed consistent identification results (Additional file 3), four pairs yielded inconsistent results (114/114′, 160/160′, 249/249′, and 566/566′), one pair had no hits (830/830′), two pairs yielded successful identification only for the TCA-B method (611/611′ and 658/658′) and one pair showed successful identification only for the TCA method (944/944′). The results of the Mascot search and information of the polypeptide hits for all the protein spots are provided in Additional file 4. The results showed that both the TCA- and TCA-B-extracted proteins from cotton leaves applied to the 2-DE analysis and that the TCA-B-extracted protein supported a high success rate in terms of protein identification.

### LC-MS/MS of proteins extracted by the three different methods

**Fig. 2:**
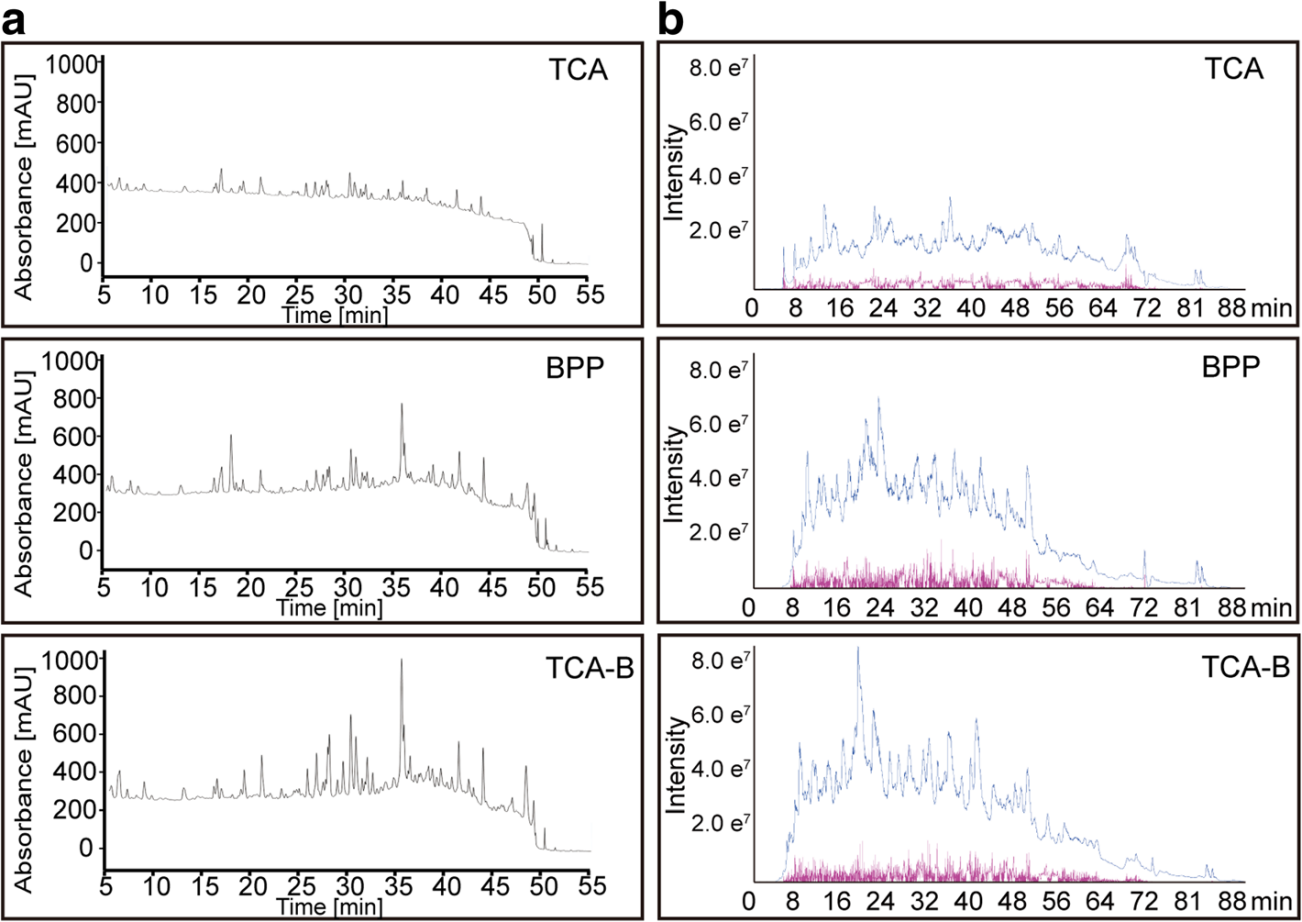
Chromatograms of the signals from HPLC fractionation (**a**) and the total ionic chromatograms from MS/MS (**b**). The digestion products of the TCA, BPP and TCA-B methods were used for HPLC fractionation (left) using a C18 reversed-phase column with a 65-min gradient. The UV absorbance from 5 to 55 min was recorded at a wavelength of 214 nm. The total ionic chromatograms (right) were acquired using an AB Sciex TripleTOF 5600 plus system, and the samples were injected and eluted using a 95-min gradient

The HPLC chromatogram of the trypsin digestion products showed the digestion performances for protein extractions from cotton leaves by the three methods (Fig. 2a). The HPLC analysis of the digestion products showed that for the TCA method, the absorption peak at 214 nm was very low throughout the HPLC fractionation process, indicating low polypeptide content in the TCA-extracted proteins after digestion and fractionation. The digestion products of the proteins extracted by the BPP and TCA-B methods showed significantly higher signals than those of TCA method (Fig. 2a).

After the HPLC fractionation, eluents were combined into 15 fractions for LC-MS/MS. The total ion chromatogram (TIC) of mass spectrometry reflects the total number of charged particles that have been sprayed into the mass spectrometer after ionization. The representative TICs from three extraction methods showed that both total ionic signal intensity of the mass spectra (blue line) and the number of MS/MS spectra acquired (red line) were all significantly lower for TCA method compared with that of BPP and TCA-B methods (Fig. 2b). The statistical information for the mass spectrometric identification showed that the total numbers of MS/MS spectra acquired for the TCA method (421,471 and 421,359 for each replicate) were significantly lower than that for the BPP (604,751 and 551,044 for each replicate) and TCA-B (655,588 and 543,553 for each replicate) methods (Table 1). Furthermore, we used ProteinPilot™ 5.0 to search against the latest protein database predicted by the upland cotton genome released by Phytozome (v12.1), with 95% confident peptide number ≥ 2 as the identification criteria. Similarly, the numbers of proteins identified for the TCA method (4621 and 4132 for each replicate) were also significantly lower than that for the BPP (6490 and 7241 for each replicate) and TCA-B (7,963 and 6747 for each replicate) methods (Table 1).

**Table 1:** Statistics of the LC-MS data from the three different protein extraction methods

Method	Total spectra	Proteins Detected	Proteins before Grouping	Distinct Peptides	Spectra Identified
BPP	604,751	7630	25,689	74,379	344,640
	551,044	8037	27,942	71,023	310,424
TCA	421,471	5115	19,100	72,728	186,080
	421,359	5018	18,395	73,657	183,002
TCA-B	655,588	8548	27,465	85,443	418,463
	543,553	7413	25,211	69,851	316,184

**Fig. 3:**
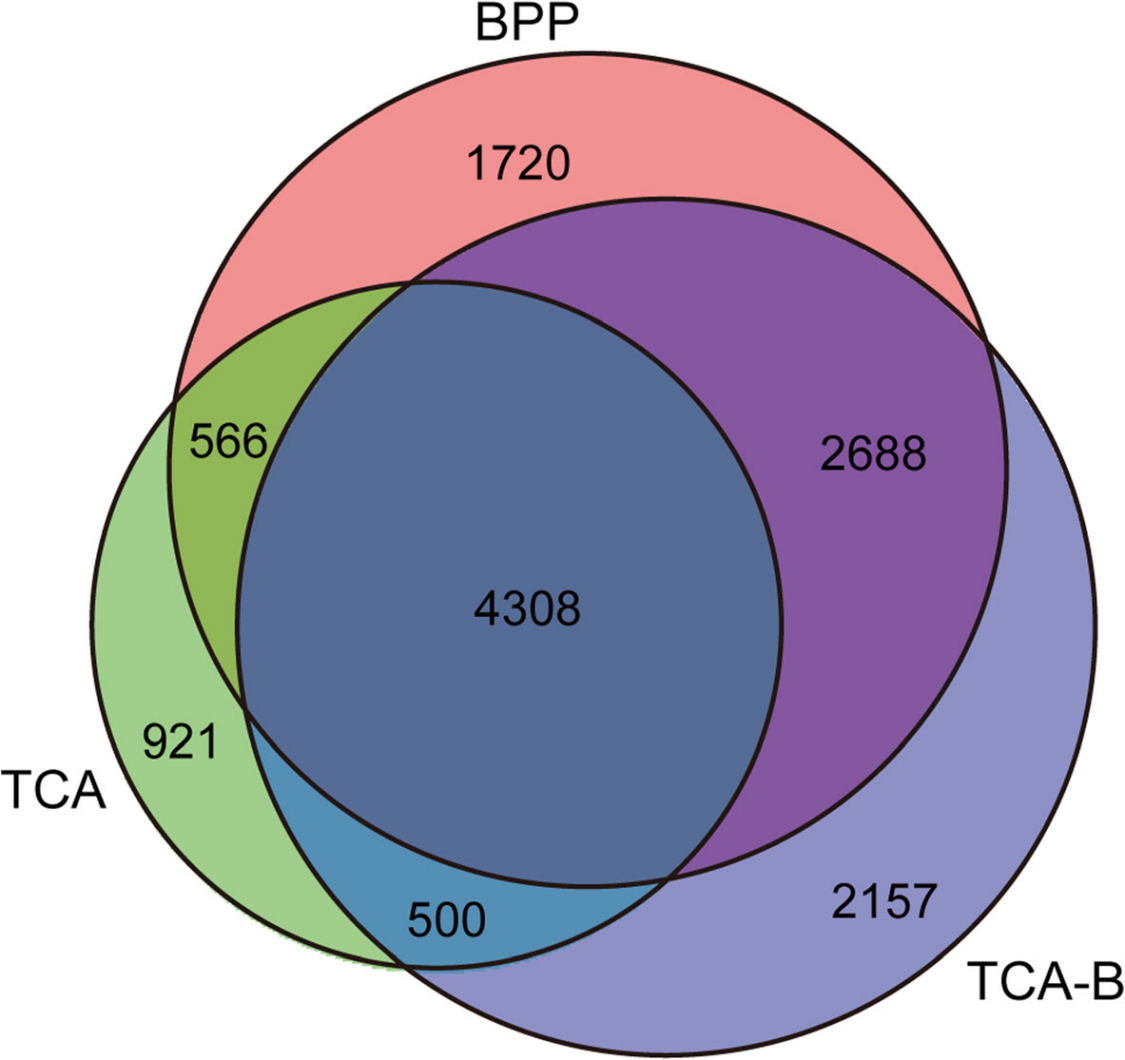
Venn diagram of the proteins identified by high-throughput LC-MS/MS. The diagram shows the distribution of the identified proteins that were extracted by the BPP, TCA and TCA-B methods

**Fig. 4:**
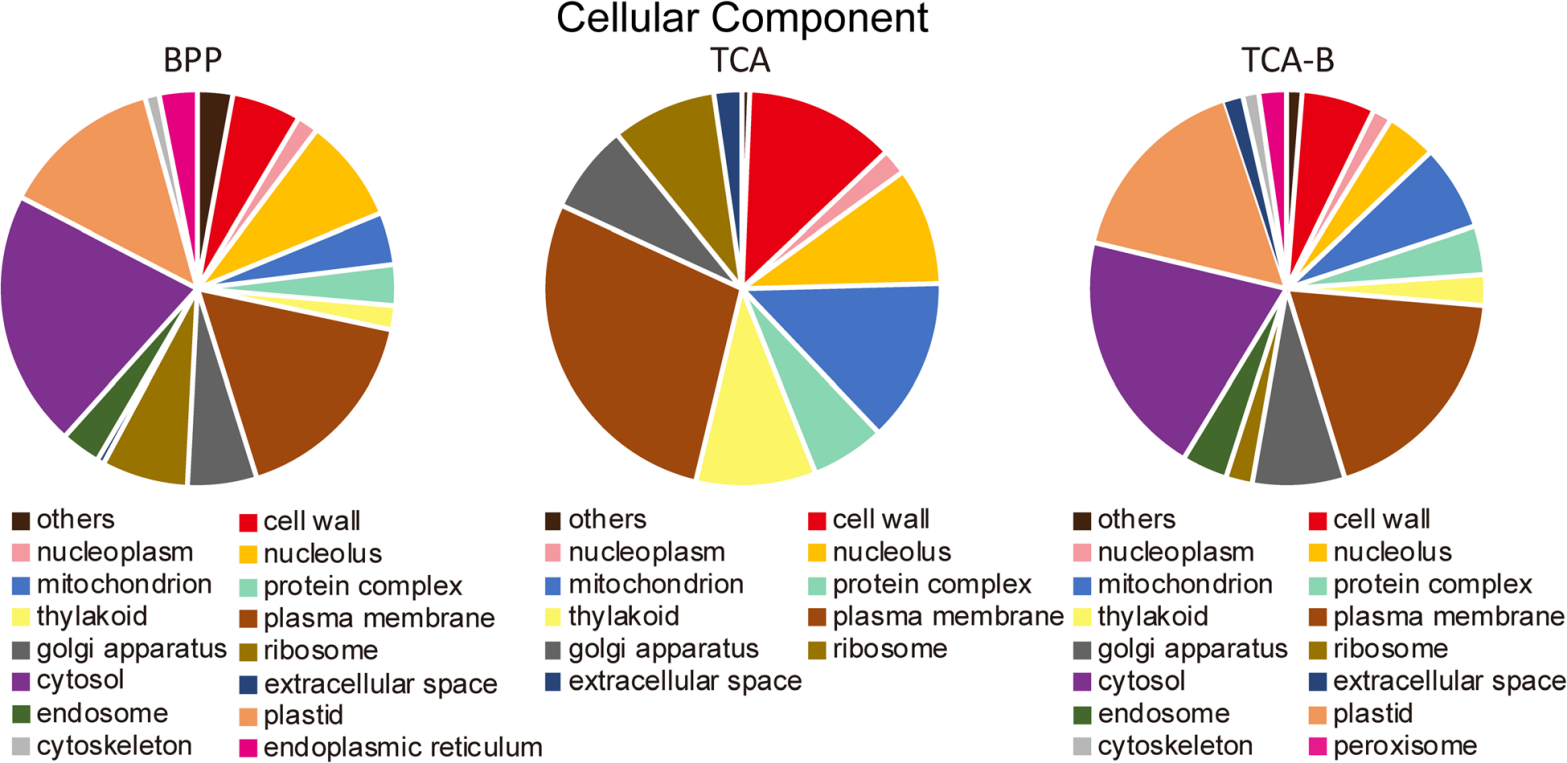
Cellular Component category of the methods-specific proteins identified by the three different extraction methods. Different colours represent the different categories of GO terms (bottom), and the area size represents the protein content in the Cellular Component category

Venn diagram analysis of the proteins identified by LC-MS/MS for the three methods revealed that 4308 proteins were simultaneously identified by all of the three methods. The numbers of specifically identified proteins for the individual methods were 1720 for BPP (red), 921 for TCA (green), and 2157 for TCA-B (blue) (Fig. 3). Detailed information including accession numbers and names of all identified proteins using the three methods can be found in the Additional file 5. To understand the characteristics of the specifically identified proteins from the three different methods, we performed GO classification. It is notable that in the Cellular Component category, a significantly lower number of GO terms were found for the proteins identified by TCA than the other two methods. Additionally, the specifically identified proteins for TCA were most frequently distributed in the plasma membrane (GO: 0005886), while the specifically identified proteins for the BPP and TCA-B methods were most frequently distributed in the cytosol (GO: 0005829) (Fig. 4). This finding suggests that different methods might have varying capabilities for enriching proteins in different sub-cellular locations. However, in the Molecular Function and Biological Progress categories, there were no significant differences in the terms harbouring the specifically identified proteins for the three methods (Additional files 6 and 7). These data indicate that TCA could not directly apply to LC-MS/MS, whereas BPP and TCA-B methods markedly improved the data quality of LC-MS/MS, which might be related to the improved protein purity after phenol extraction.

### Association analysis of 2-DE and LC-MS/MS

**Fig. 5:**
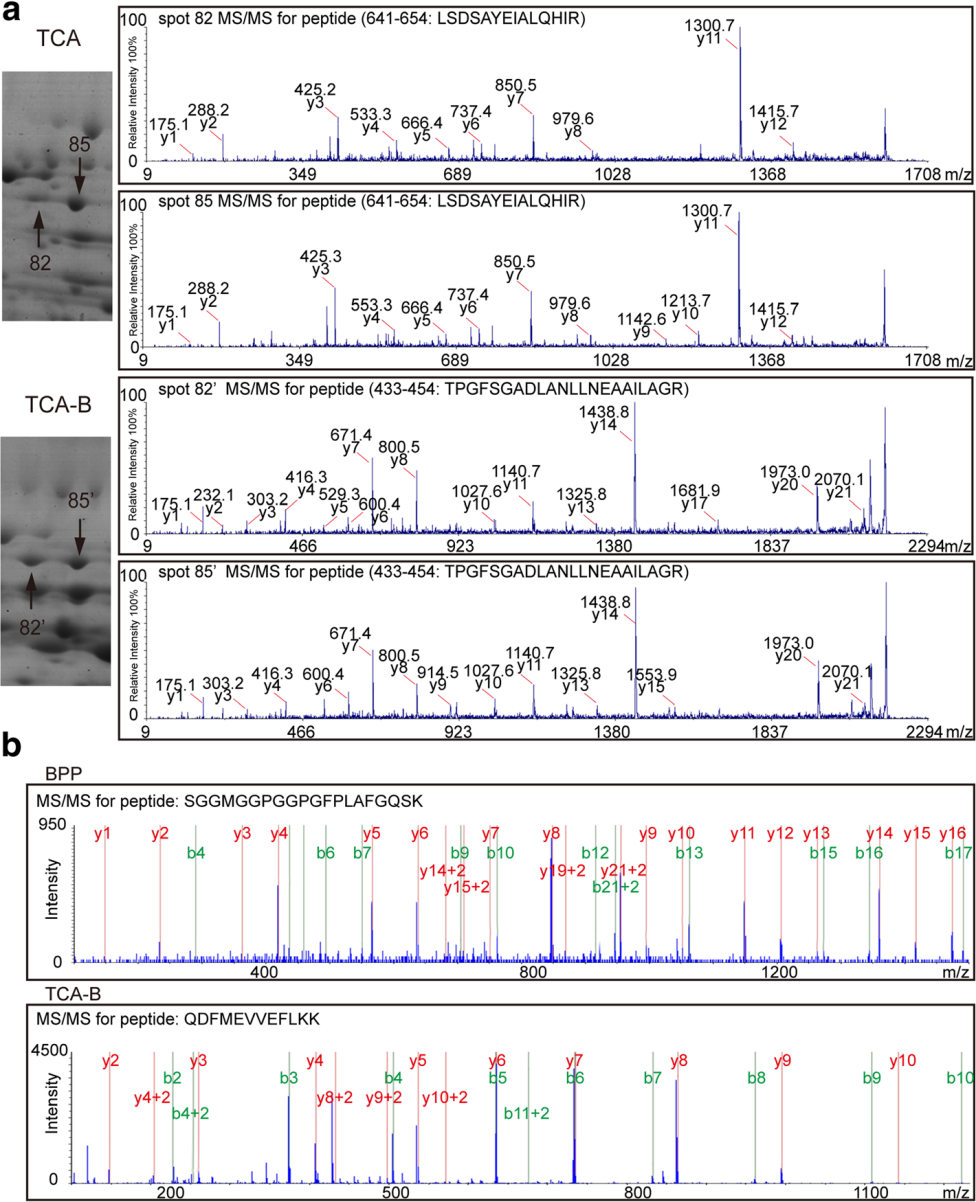
Mass spectrometric identification of ATP-dependent zinc metalloprotease FTSH 8 in 2-DE gels and by high-throughput LC-MS/MS. **a** Enlarged images showing the protein spots (spots 82, 85 and spots 82′, 85′) identified as ATP-dependent zinc metalloprotease FTSH 8 in the 2-DE gels of TCA and TCA-B (left), and the representative MALDI-TOF/TOF spectra of spots 82 and 85 (top right) and spots 82′ and 85′ (bottom right). **b** The representative MS/MS spectra of FTSH 8 identified from high-throughput LC-MS/MS of the BPP (top panel) and TCA-B (bottom panel) methods

Although LC-MS/MS greatly increases the number of identified proteins, gel-based proteomic techniques play an irreplaceable role in identifying protein isoforms [11]. Therefore, an association analysis of 2-DE and LC-MS/MS data from the same sample contributes to the identification of proteins in different isoforms. One representative example in this study was the ATP-dependent zinc metalloprotease FTSH 8, of which two isoforms (spots 82/82′ and 85/85′) were identified on the 2-DE gels of proteins extracted by the TCA and TCA-B methods. The enlarged regions of the protein isoforms on the 2-DE gels and their representative MS/MS spectra are shown (Fig. 5a). The peptide sequence mapped from spots 82 and 85 was “LSDSAYEIALQHIR”, and “TPGFSGADLANLLNEAAILAGR” for spots 82′ and 85′. However, in the LC-MS/MS results, this specific protein was not detected from the TCA method. The representative MS/MS spectra of this protein from the BPP and TCA-B methods are shown (Fig. 5b). The peptide hits were “SGGMGGPGGPGFPLAFGQSK” and “QDFMEVVEFLKK”, respectively. These results suggested that only TCA-B supported the simultaneous identification of this enzyme by both 2-DE and LC-MS/MS techniques.

## Discussion

Extensive studies have reported that phenol-extracted proteins work better for 2-DE than the TCA-extracted proteins [22, 25, 26]. The phenol-based extraction method performs well in removing the salt ions from tissues and yielding a high-purity protein extracts, and it is widely applied to the 2-DE study of high-salt plants [27]. However, in the present study, the BPP method obtained a low protein yield (Additional file 2) and produced unclear 2-DE maps, which did not apply to 2-DE analysis of proteins from cotton leaves (Fig. 1). In a recent study, we also found that BPP is not suitable for 2-DE analysis of cotton leaves, and in which TCA was used [24]. This finding may be due to the fact that cotton is a plant rich in polysaccharides and polyphenols [28]. Endogenous phenols have been shown to bind reversibly to proteins that results into charge heterogeneity and streaks in the 2-DE gels; additionally, phenol and benzoquinone, a derivative of phenol formed by oxidation, can also modify the protein and interfere with the phenol extraction process [23, 29, 30]. These may explain the reason why the cotton leaves proteins extracted using BPP method was not suitable for 2-DE analysis. In high-throughput proteomics, the performance of HPLC fractionation (Fig. 2a), total ion chromatograms of mass spectra (Fig. 2b) and the general situation of mass spectrometric identification (Table 1) showed that TCA extraction method was not suitable for the LC-MS/MS study of cotton leaves. This result may be attributable to the fact that it is difficult to re-dissolve TCA-extracted proteins [20], which contain many impurities and that it is difficult to completely digest the TCA-extracted proteins by filter-aided sample preparation method. These findings may explain the reason why the cotton leaves proteins extracted by TCA method did not apply to the LC-MS/MS study.

The improved TCA-B method retained the advantages of the other two protein extraction methods which could obtain a higher protein yield than BPP method (Additional file 2) and got clear spots in 2-DE map (Fig. 1) from cotton leaves proteins. The 84% proteins identified information of selected spots were consistent between TCA and TCA-B methods and the two pairs of protein spots (spots 611/611′ and 658/658′) were only successfully identified from TCA-B (Additional file 3), which means the improved TCA-B method have the similar effect compared with the TCA method in 2-DE gel analysis of cotton leaves. In high-throughput proteomics, the TCA-B and BPP methods were superior to TCA with regards to the performance of HPLC fractionation (Fig. 2a), the total ion signal strength of the mass spectra (Fig. 2b) and the number of total peptides identified by mass spectrometry (Table 1). Additionally, TCA-B obtained the highest total number of identified proteins as well as the highest number of individually identified proteins (Fig. 3). All these findings indicate that the TCA-B protein extraction method can satisfy the needs of both 2-DE and LC-MS/MS studies of cotton leaves.

The function enrichment of method-specifically extracted proteins after LC-MS/MS from three protein extraction methods was performed by GO analysis. We found that these proteins were enriched according to their different functions (Fig. 4, Additional files 6 and 7). The six GO terms could be identified for specific proteins in the BPP- and TCA-B-extracted proteins (cytosol, endosome, plastid, cytoskeleton, peroxisome and endoplasmic reticulum). The TCA-extracted proteins specifically identified by LC-MS/MS were poorly enriched in these GO terms. However, it does not necessarily mean that TCA cannot extract proteins with these functions because these functional proteins may be present in the proteins co-extracted by the two or three methods. At present, many protein extraction method studies are only focus on one proteomic technique, the 2-DE or high-throughput proteomic techniques [31–33]. The use of proteins extracted by the same method for both 2-DE and LC-MS/MS studies can avoid false differential abundant proteins associated with different extraction methods, allowing identification of a higher number of proteins with various isoforms [10, 34]. Additionally, the differentially expressed proteins identified from the LC-MS/MS and 2-DE techniques could be different [35, 36]. Herein, we found that for the ATP-dependent zinc metalloprotease FTSH 8, both TCA-B and TCA could reflect the visualized information for protein isoforms in the 2-DE gels. By contrast, FTSH 8 could be only identified by BPP and TCA-B in the LC-MS/MS study (Fig. 5). This finding demonstrates that applying TCA-B simultaneously for both 2-DE and LC-MS/MS studies allows the identification of more proteins over a broader range and with high sensitivity. Additionally, TCA-B supports the identification of proteins in different variable cleavage forms by 2-DE, and thus it can be better used to analyse the proteome studies of different physiological processes in polysaccharide and polyphenol-rich plants.

## Conclusions

In summary, the BPP-extracted proteins from cotton leaves, the plant tissue rich in polysaccharides and polyphenols, did not work well with 2-DE analysis, whereas the TCA-extracted proteins were not well compatible with LC-MS/MS. The improved TCA-B method not only supports the acquisition of high-quality 2-DE maps but also ensures a significantly increased number of proteins identified by LC-MS/MS. In addition, the association analysis of the 2-DE and LC-MS/MS data from TCA-B contributes to the identification for protein isoforms in cotton leaves, and it provides a new alternative for further study of the different protein isoforms in plant tissues rich in polysaccharides and polyphenols.

## Methods

### Plant materials

Upland cotton variety Xuzhou 142 were planted in an artificial climate chamber under the growing conditions of 60% humidity, 34 °C, and a 14 h/10 h light/dark cycle. Leaves from two-week-old seedlings were harvested and frozen in liquid nitrogen immediately and then stored at − 80 °C until use.

### Procedures of protein extraction methods

#### Protocol 1: TCA/acetone method

The TCA method was performed as previously described with slight modification [37]. Briefly, 1 g of cotton leaves were ground thoroughly to powder in liquid nitrogen with 7% polyvinylpyrrolidone (*w*/w) and then the powders were placed in a 10 ml tube with 5 ml of pre-chilled acetone, 10% trichloroacetic acid and 0.07% β-mercaptoethanol, then vortexed for 1 min and incubated overnight at − 20 °C for precipitation. The tube was centrifuged for 15 min at 4 °C and 13,000 rpm. The supernatant was discarded and the pellets were then washed twice using pre-chilled acetone and centrifuged in the same condition after each washing then discarded the supernatant. Protein pellets were air dried at room temperature then dissolved by 200 μl lysis buffer (7 M urea, 2 M thiourea, 2% CHAPS, 1.7% PMSF, 50 mM DTT) and stored at − 80 °C until use.

#### Protocol 2: borax/PVPP/phenol (BPP) method

The BPP method was performed according to our previous work [27]. Briefly, 1 g of cotton leaves were ground thoroughly to powder in liquid nitrogen with 7% polyvinylpyrrolidone (w/w). The powders were placed into a new tube with 5 ml BPP buffer and vortexed thoroughly for 10 min at room temperature. Then, 10 ml of Tris saturated phenol (pH 8.0) was added, and the samples were subsequently centrifuged at 4 °C and 13,000 rpm for 15 min (the centrifuge conditions were coincident in all steps). The supernatant was placed into a new tube, and added equal volume BPP buffer then vortexed thoroughly for 10 min at room temperature and centrifuged in the same condition. The supernatant was then placed into a new tube, and 5 volumes of ammonium sulphate super-saturated buffer were added to precipitate the proteins at − 20 °C for at least 6 h. Afterward, the tubes were centrifuged and discarded the supernatant. The pellets were washed twice using pre-chilled methanol and acetone and then centrifuged, discarding the supernatant after each wash. The pellets were then air dried, subsequently dissolved using 200 μl of lysis buffer and then stored at − 80 °C until use.

#### Protocol 3: TCA-B method

We constructed an improved protein extraction method based on the above two methods, which was named as the TCA-B method. Briefly, the total proteins from 1 g of cotton leaves were firstly extracted using the TCA method. Then, the dissolved proteins were placed into a new tube, 3 volumes of BPP buffer were added. The samples were vortexed thoroughly for 10 min at room temperature, and then, 2 volumes of Tris saturated phenol (pH 8.0) were added and centrifuged at 4 °C and 15,000 rpm for 20 min to better remove the fragment. The supernatant was placed into a new tube, and 5 volumes of ammonium sulphate super-saturated buffer were added to precipitate the proteins at − 20 °C overnight. Afterward, the samples were centrifuged in the same condition, and the pellets were washed twice using pre-chilled methanol and acetone, the supernatant was discarded after each wash. Finally, the pellets were air dried and then recovered using 100 μl of the lysis buffer before storage at − 80 °C until use.

### Two-dimensional polyacrylamide gel electrophoresis

Protein quantification was performed following the Bradford method [38] using a UV-160 spectrophotometer (Shimadzu, Kyoto, Japan). The 2-DE of the cotton leaf proteins were conducted using 24 cm, pH 4–7 gradient-immobilized pH gradient strips (GE Healthcare, Uppsala, Sweden) with 800 μg of proteins. To analyse the quality of proteins obtained by the three extraction methods, the 2-DE experiments were performed as described [25, 39]. Each extraction method had three replicates. The gel spots were visualized by the GAP staining [40] and then analysed using Image Master 2D Platinum software (Version 5.0, GE Healthcare Life Sciences).

### Protein identification by MALDI TOF/TOF MS

Fifty pairs of corresponding protein spots were randomly selected from the 2-DE gels of the TCA and TCA-B methods. The protein spots were excised and in-gel digested with modified bovine trypsin (cat. no. 11418025001, Roche, Basel, Switzerland) as described [25]. The digested peptide fragments were identified by MALDI TOF/TOF MS [25, 39]. The deduced protein sequences of upland cotton were downloaded from the online database Phytozome (https://phytozome.jgi.doe.gov/pz/portal.html) [41]. A local Mascot search was performed as follows: 30 ppm maximum mass error, MH^+^ monoisotopic mass values, oxidation of methionine allowed, one missed cleavage, permitted fixed modification of carbamidomethylation, trypsin as the enzyme. Matches were classified as good if they had a threshold score higher than 33 (*p* < 0.05).

### Trypsin digestion of total protein samples

The 400 μg of total protein from each extraction method was used for trypsin digestion, and the digestion was performed as previously described [42].

### LC-MS/MS analysis

The LC-MS/MS analysis was performed as previously described [15]. Each extraction method had two replicates. The digested peptides were vacuum freeze-dried and dissolved into 100 μl of mobile phase A (20 mM ammonium formate, pH 10). A gemini-NX C18 column (4.6 × 250 mm, 5 μm 110 Å, Phenomenex, Shanghai, China) and a UPLC system (Ultimate™ 3000, Dionex, Thermo, Shanghai, China) were used to fractionate each sample into 15 fractions using a 65-min gradient of increasing mobile phase B (20 mM ammonium formate in acetonitrile) from 5 to 38%. The subsequent LC-MS/MS experiment was carried out on a Triple TOF 5600 plus system (AB Sciex, Shanghai, China) coupled with an UltiMate™ 3000 RSLCnano (Dionex, Thermo, Shanghai, China). The resulting MS spectra were acquired across the mass range of 350–1500 m/z in high resolution mode (> 30,000) and the accumulation time is 250 ms per spectrum. A maximum of 40 precursors per cycle with a minimum accumulation time of 100 ms for each precursor and dynamic exclusion for 20 s were chosen for fragmentation from each MS spectrum. Tandem mass spectra were recorded in high sensitivity mode (resolution > 15,000) using rolling collision energy [43]. Eight microlitres of each fraction was loaded and desalted by a 5 mm pre-column. Then, the sample was eluted using a 95-min gradient of mobile phase B (0.1% formic acid in 98% acetonitrile). The acquired MS data were analysed against the downloaded cotton protein database from Phytozome using ProteinPilot™ 5.0 software (AB Sciex, Shanghai, China). Proteins with 95% confidence intervals, > 2 peptides and an unused score > 1.3 were considered successfully identified.

### Gene ontology analysis

Sequences of all identified proteins (JGI available accession numbers are available in Additional file 5) were submitted to the online AgBase database (http://www.agbase.msstate.edu/cgi-bin/tools/GOanna.cgi) to obtain their functional annotation. GO distribution analysis was performed using GO slim (http://agbase.arizona.edu/cgi-bin/tools/goslimviewer_select.pl).

## Additional files

Additional file 1: A flowchart showing the experimental protocols of the three different protein extraction methods (**a**). The detailed extraction information of the BPP, TCA and TCA-B methods, respectively (**b**). (PDF 601 kb)

Additional file 2: Protein concentrations and yields from the three different protein extraction methods. (XLS 65 kb)

Additional file 3: MS/MS identification of the 50 pairs of protein spots from the TCA and TCA-B gels. (XLS 373 kb)

Additional file 4: Detailed information for the 50 pairs of protein spots identified by MALDI TOF/TOF. (PDF 3258 kb)

Additional file 5: List of all proteins identified by the high-throughput LC-MS/MS. (XLS 5478 kb)

Additional file 6: Molecular Function category of the specific proteins identified by different methods. Different colours represent different functional categories (bottom) and the area size represents the protein content in the molecular function category. (PDF 209 kb)

Additional file 7: Biological Process category of the specific proteins identified by different methods. Different colours represent different functional categories (bottom) and the area size represents the protein content in the biological process category. (PDF 238 kb)

## Abbreviations

2-DE: Two-dimensional electrophoresis
BPP: Borax/PVPP/phenol
DIGE: Differential in-gel electrophoresis
GO: Gene Ontology
iTRAQ: Isobaric tags for relative and absolute quantitation
LC-MS: Liquid chromatography-tandem mass spectrometry
MALDI-TOF/TOF MS: Matrix-assisted laser desorption/ionization time-of-flight/time-of-flight mass spectrometry
PMF: Peptide mass fingerprinting
TCA: Trichloroacetic acid/acetone
TCA-B: TCA-combined BPP
TIC: Total ion chromatogram
TM-1: Texas Marker-1
