# Supplementary figures and images for: An improved protein extraction method applied to cotton leaves is compatible with 2-DE and LC-MS
Source: BMC Genomics. 2019 Apr 11;20:285. doi: 10.1186/s12864-019-5658-5 (PMC6458646; doi:10.1186/s12864-019-5658-5)

## Additional file 1

a

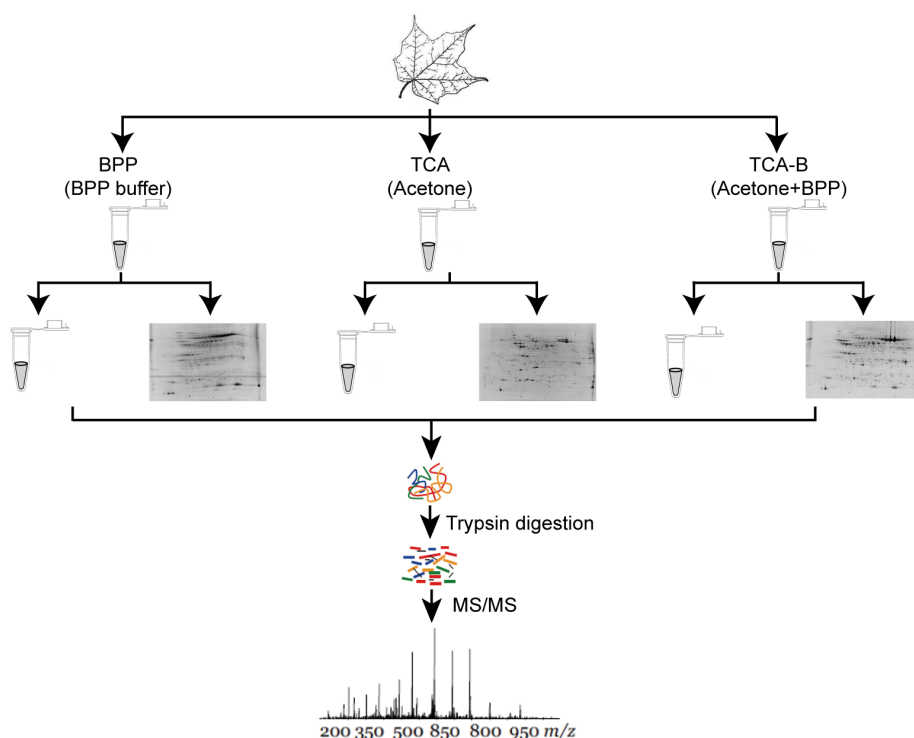

b

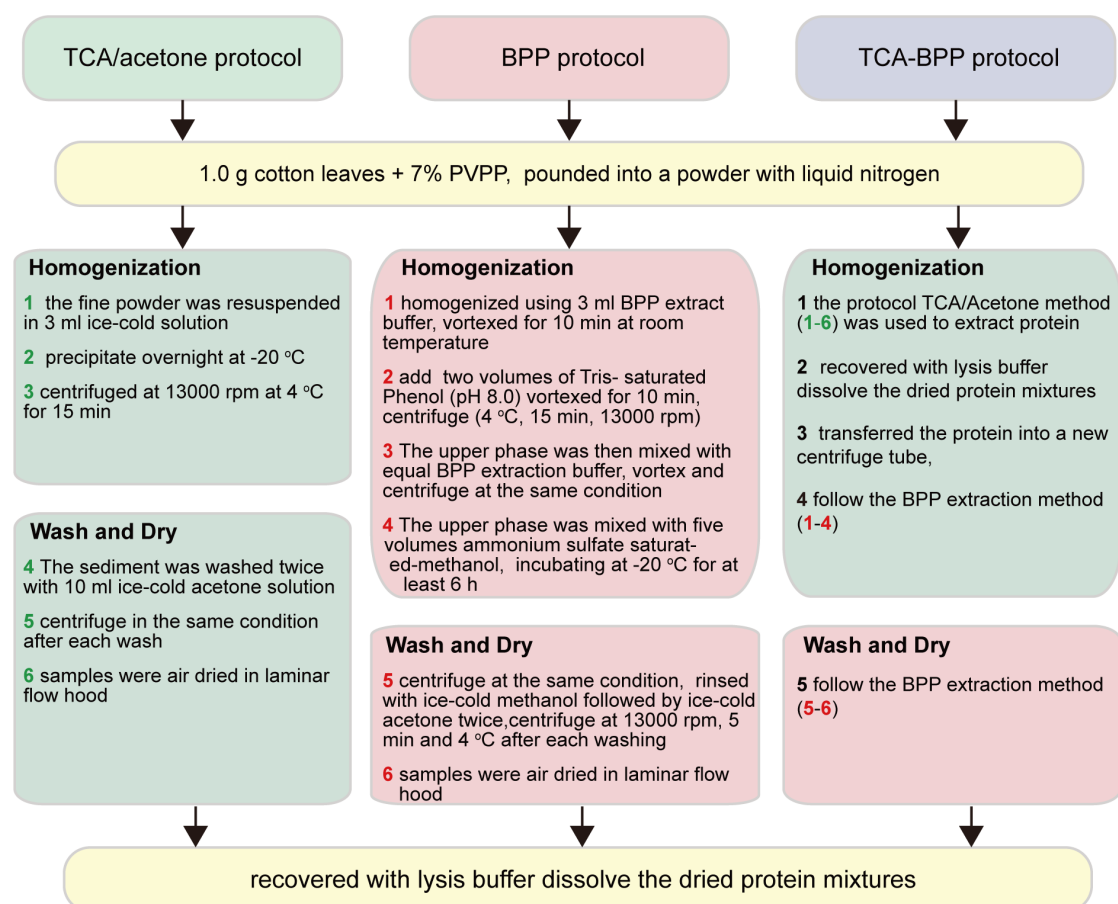

Supplement: Supplementary file 1 — A flowchart showing the experimental protocols of the three different protein extraction methods (a). The detailed extraction information of the BPP, TCA and TCA-B methods, respectively (b). (PDF 601 kb) [file 12864_2019_5658_MOESM1_ESM.pdf]

Additional file 6

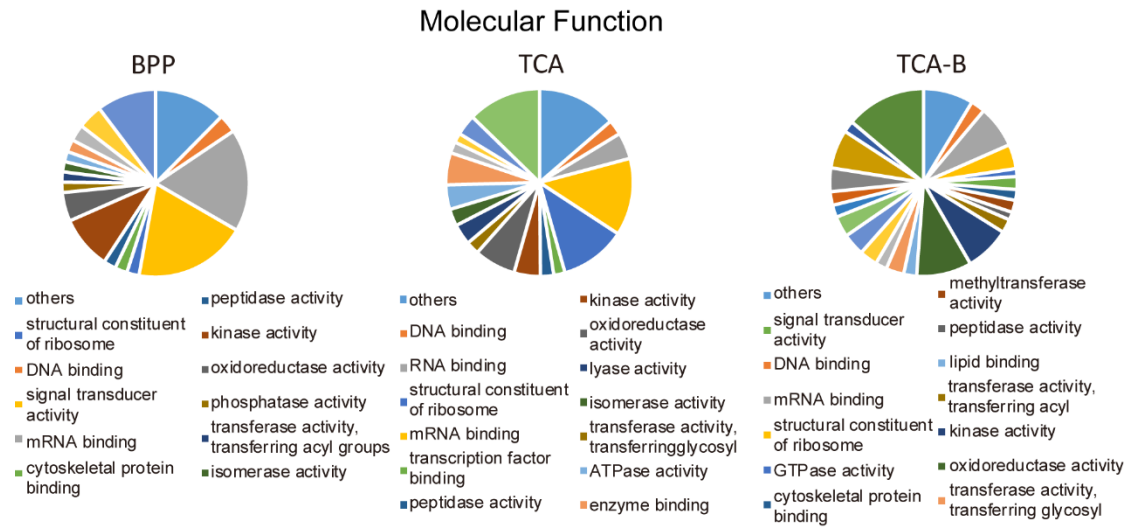

Supplement: Supplementary file 6 — Molecular Function category of the specific proteins identified by different methods. Different colours represent different functional categories (bottom) and the area size represents the protein content in the molecular function category. (PDF 209 kb) [file 12864_2019_5658_MOESM6_ESM.pdf]

Additional file 7

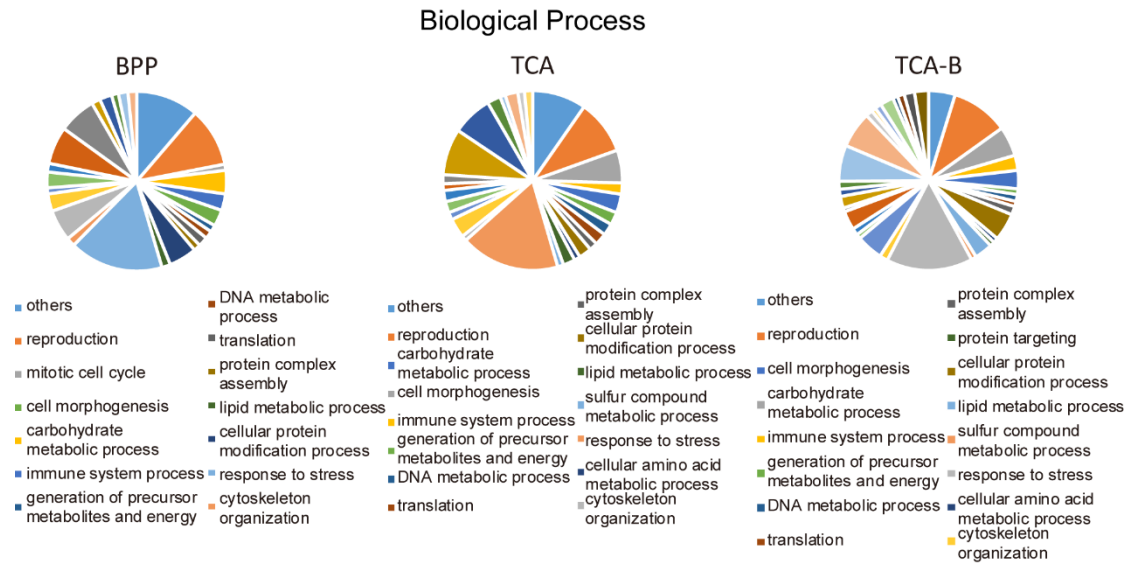

Supplement: Supplementary file 7 — Biological Process category of the specific proteins identified by different methods. Different colours represent different functional categories (bottom) and the area size represents the protein content in the biological process category. (PDF 238 kb) [file 12864_2019_5658_MOESM7_ESM.pdf]
