# Supplementary material for: An improved protein extraction method applied to cotton leaves is compatible with 2-DE and LC-MS
Source: BMC Genomics. 2019 Apr 11;20:285. doi: 10.1186/s12864-019-5658-5 (PMC6458646; doi:10.1186/s12864-019-5658-5)
Supplement: Supplementary file 4 — Detailed information for the 50 pairs of protein spots identified by MALDI TOF/TOF. (PDF 3258 kb) [file 12864_2019_5658_MOESM4_ESM.pdf]

Additional file 4

Spot No.: A1

Protein name: Translation elongation factor EFG/EF2 protein

Peptide sequences:

R.INIIDTPGHVDFTLEVER.A;K.FEYADIPADLQELAEEYR.S;K.IMTDPFVGSLTFVR.V;R.AVLVDGSYHDVDSSVLAFQLAAR.G

Accession No.: Gohir.A11G019900.1.p

Mascot score: 180 **Sequence coverage %:** 9

Calculated Mr: 86600 **Calculated *p*I:** 5.54

PFF Searched Score:

Ions score is -10*Log(P), where P is the probability that the observed match is a random event.
Individual ions scores > 33 indicate identity or extensive homology (p<0.05).
Protein scores are derived from ions scores as a non-probabilistic basis for ranking protein hits.

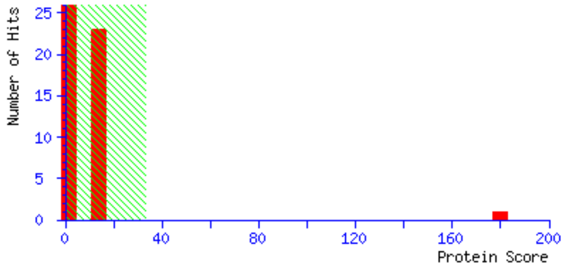

Matched peptide sequences: shown in Bold Red:

```
  1 MAAETALRVS SSSSTVCNLN GFQRRPTPLS SSTRFLGLRP RASSSSISSS
 51 LSQFMGSVRI GSRLPISLQQ KGKRRNFSLF AMAADESKRA VPLKDYRNIG
101 IMAHIDAGKT TTTERILYYT GRNYKIGEVH EGTATMDWME QEQERGITIT
151 SAATTTFWKD HRINIIDTPG HVDFTLEVER ALRVLDGAIC LFDSVAGVEP
201 QSETVWRQAD KYGVPRICFV NKMDRLGANF FRTRDMIVTN LGAKPLVIQL
251 PVGAEDNFKG VIDLVKMKAV LWSGEELGAK FEYADIPADL QELAEEYRSQ
301 MIETIVELDD QAMENYLEGV EPDEETIKKL IRKGTIGISF VPVLCGSAFK
351 NKGVQPLLDA VMDYLPSPLE LPAMKGTDPE NPEVIIERTA SDEEPFSGLA
401 FKIMTDPFVG SLTFVRVYSG KLAAGSYVLN ANKGKKERIG RLLEMHANSR
451 EDVKVALAGD IVALAGLKDT ITGETLSDPD HPIVLERMDF PDPVIKVAIE
501 PKTKADVDKM ANGLIKLAQE DPSFHFSRDE EINQTVIEGM GELHLEIIVD
551 RLKREFKVEA NVGAPQVNYR ESISKVSEVK YVHKKQSGGQ GQFADITVRF
601 EPMDAGSGYE FKSEIKGGAV PKEYIPGVMK GLEECMCNGV LAGFPVVDVR
651 AVLVDGSYHD VDSSVLAFQL AARGAFREGI RKAGPRMLEP IMKVEVVTPE
701 EHLGDVIGDL NSRRGQINSF GDKPGGLKVV DALVPLAEMF QYVSTLRGMT
751 KGRASYTMQL AKFDVVPQHI QNELATKQQE VVA
```

Spot No.: A2

Protein name: Chloroplast heat shock protein 70-2

Peptide sequences:

K.AVVTVPAYFNDSQR.T;R.VKFEELCSDLLDR.L;K.DIDEVILVGGSTR.I;R.IPAVQELVR.K;K.SEVFSTAADGQTSVEINVLQGER.E

Accession No.: Gohir.D09G212600.1.p

Mascot score: 239 **Sequence coverage %:**10

Calculated Mr: 75693 **Calculated *p*I:** 5.17

PFF Searched Score:

Ions score is -10*Log(P), where P is the probability that the observed match is a random event.
Individual ions scores > 34 indicate identity or extensive homology (p<0.05).
Protein scores are derived from ions scores as a non-probabilistic basis for ranking protein hits.

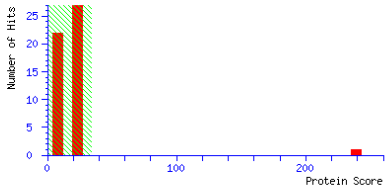

Matched peptide sequences: shown in Bold Red:

1 MASSTAQIHV LGGMGFDSSR KPNQHSSRTV FFGQRLGKTS PLNATFLRLA
51 KTKNSNGKGY NVGPVRVVNE KVVGIDLGTT NSAVAAMEGG KPTIVTNAEG
101 QRTTPSVVAY TKSGDRLVGQ IAKRQAVVNP ENTFFSVKRF IGRKMSEVDE
151 EAKQVSYKVI RDENGNVKLE CPAISKQFAA EEISAQVLRK LVDDASKFLS
201 ESVTK**AVVTV PAYFNDSQR**T ATKDAGRIAG LEVLRIINEP TAASLAYGFE
251 KKNNETILVF DLGGGTFDVS VLEVGDGVFE VLSTSGDTHL GGDDFDKRIV
301 DWLAENFKRD EGIDLLKDKQ ALQRLTEAAE KAKMELSTLT QTNISLPFIT
351 ATADGPKHIE TTLTR**VKFEE LCSDLLDR**LK KPVENALGDA KLSFK**DIDEV**
401 **ILVGGSTRIP AVQELVR**KMT GKEPNVTVNP DEVVALGAAV QAGVLAGDVS
451 DIVLLDVTPL SLGLETLGGV MTKIIPRNTT LPTSK**SEVFS TAADGQTSVE**
501 **INVLQGER**EF VRDNKSLGSF RLDGIPPAPR GVPQIEVKFD IDANGILSVT
551 AVDKGTGKKQ DITITGASTL PGDEVDRMVK EAERFAQEDK EKRDAIDTKN
601 QADSAVYQTE KQLKELGDKV PGPVKEKVDA KLQELKDAIS GGSTQGMKDA
651 MAALNQEVMQ LGQSLYNQPS AGSAAGPAPG GETGRSDSSN KGSDEDVIDA
701 DFTDSK

Spot No.: A4

Protein name: ATP-dependent zinc metalloprotease FTSH 8

Peptide sequences:

R.FLEYLDKDR.V;R.ADILDSALLRPGR.F;K.KFETDVSFDVIAMR.T;R.TPGFSGADLANLLNEAAILAGR.R;R.ISDSAYEIALQHIR.N

Accession No.: Gohir.A01G022900.1.p

Mascot score: 213 **Sequence coverage %:**10

Calculated Mr: 74627 **Calculated *p*I:** 6.24

PFF Searched Score:

Ions score is -10*Log(P), where P is the probability that the observed match is a random event.
Individual ions scores > 34 indicate identity or extensive homology (p<0.05).
Protein scores are derived from ions scores as a non-probabilistic basis for ranking protein hits.

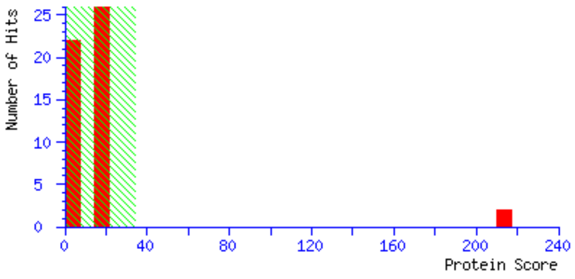

Matched peptide sequences: shown in Bold Red:

```
  1 MAASSACLLG NGPSTHRTKS KLSKEFYGRN IVFTPAISSL GRKSNAVLVK
 51 ASLKHKQHEG RRGFLKSLLG TAGIGVPALL GNGKAYADEQ GVSSSRMSYS
101 RFLEYLDKDR VKKVDLFENG TIAIVEAVSP ELGNRVQRVR VQLPGLSQEL
151 LQKFREKNID FAAHNAQEDS GSLLFNLIGN LAFPLILIGG LFLLSRRSSG
201 GMGGPGGPGF PLAFGQSKAK FQMEPNTGVT FDDVAGVDEA KQDFMEVVEF
251 LKKPERFTAV GARIPKGVLL IGPPGTGKTL LAKAIAGEAG VPFFSISGSE
301 FVEMFVGVGA SRVRDLFKKA KENAPCIVFV DEIDAVGRQR GTGIGGGNDE
351 REQTLNQLLT EMDGFEGNTG IIVIAATNRA DILDSALLRP GRFDRQVTVD
401 VPDIRGRTEI LKVHGSNKKF ETDVSFDVIA MRTPGFSGAD LANLLNEAAI
451 LAGRRGKTAI SSKEIDDSID RIVAGMEGTV MTDGKSKSLV AYHEVGHAIC
501 GTLTPGHDPV QKVTLIPRGQ ARGLTWFIPS DDPTLISKQQ LFARIVGGLG
551 GRAAEEIIFG EPEVTTGAAG DLQQITGLAK QMVVTFGMSE IGPWSLMDSS
601 AQSADVIMRM MARNSMSEKL AEDIDTAVKR ISDSAYEIAL QHIRNNREAI
651 DKIVEVLLEK ETMSGDEFRA ILSEFVEIPA ENQVPPSVPT PVSV
```

Spot No.: A6

Protein name: ATP-dependent zinc metalloprotease FTSH 8

Peptide sequences:

K.KVDLFENGTIAIVEAVSPELGNR.V;K.AKENAPCIVFVDEIDAVGR.Q;K.ENAPCIVFVDEIDAVGR.Q;R.ADILDSALLRPGR.F;K.KFETDVSFDVIAMR.T;R.TPGFSGADLANLLNEAAILAGR.R;R.ISDSAYEIALQHIR.N

Accession No.: Gohir.A01G022900.1.p

Mascot score: 488 **Sequence coverage %:** 15

Calculated Mr: 74627 **Calculated *p*I:** 6.24

PFF Searched Score:

Ions score is -10*Log(P), where P is the probability that the observed match is a random event.
Individual ions scores > 33 indicate identity or extensive homology (p<0.05).
Protein scores are derived from ions scores as a non-probabilistic basis for ranking protein hits.

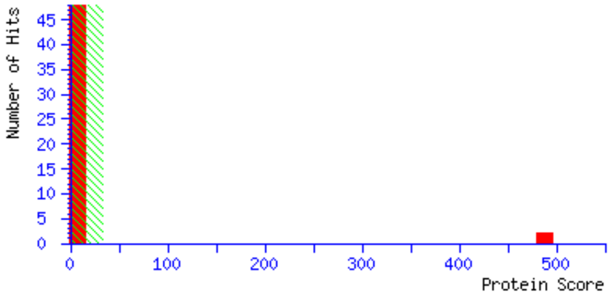

Matched peptide sequences: shown in Bold Red:

```
  1 MAASSACLLG NGPSTHRTKS KLSKEFYGRN IVFTPAISSL GRKSNAVLVK
 51 ASLKHKQHEG RRGFLKSLLG TAGIGVPALL GNGKAYADEQ GVSSSRMSYS
101 RFLEYLDKDR VKKVDLFENG TIAIVEAVSP ELGNRVQRVR VQLPGLSQEL
151 LQKFREKNID FAAHNAQEDS GSLLFNLIGN LAFPLILIGG LFLLSRRSSG
201 GMGGPGGPGF PLAFGQSKAK FQMEPNTGVT FDDVAGVDEA KQDFMEVVEF
251 LKKPERFTAV GARIPKGVLL IGPPGTGKTL LAKAIAGEAG VPFFSISGSE
301 FVEMFVGVGA SRVRDLFKKA KENAPCIVFV DEIDAVGRQR GTGIGGGNDE
351 REQTLNQLLT EMDGFEGNTG IIVIAATNRA DILDSALLRP GRFDRQVTVD
401 VPDIRGRTEI LKVHGSNKKF ETDVSFDVIA MRTPGFSGAD LANLLNEAAI
451 LAGRRGKTAI SSKEIDDSID RIVAGMEGTV MTDGKSKSLV AYHEVGHAIC
501 GTLTPGHDPV QKVTLIPRGQ ARGLTWFIPS DDPTLISKQQ LFARIVGGLG
551 GRAAEEIIFG EPEVTTGAAG DLQQITGLAK QMVVTFGMSE IGPWSLMDSS
601 AQSADVIMRM MARNSMSEKL AEDIDTAVKR ISDSAYEIAL QHIRNNREAI
651 DKIVEVLLEK ETMSGDEFRA ILSEFVEIPA ENQVPPSVPT PVSV
```

Spot No.: A8

Protein name: Chaperonin-60alpha

Peptide sequences:

K.EIAFDQSSR.A;K.LADAVGLTLGPR.G;R.AIELPNAMENAGAALIR.E;R.GYISPQFVTNPEK.L;K.LICEFENAR.V;K.AVLQDIAILTGAEFQANDLGLLIENTSVEQLGIAR.K;K.DSTQIIADAASKDEIQAR.V

Accession No.: Gohir.A13G232000.1.p

Mascot score: 623 **Sequence coverage %:** 19

Calculated Mr: 62143 **Calculated *p*I:** 5.15

PFF Searched Score:

Ions score is -10*Log(P), where P is the probability that the observed match is a random event.
Individual ions scores > 34 indicate identity or extensive homology (p<0.05).
Protein scores are derived from ions scores as a non-probabilistic basis for ranking protein hits.

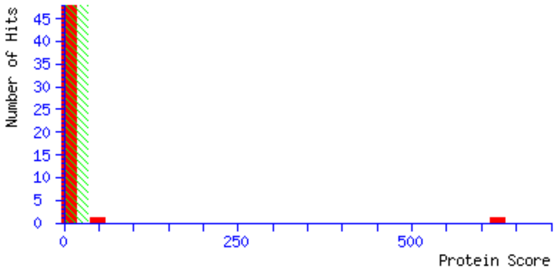

Matched peptide sequences: shown in Bold Red:

```
  1 MASANALSSA SILCSPNKGS LRRKGNQRQN QRVNYRQGNN RFGVKACAKE
 51 IAFDQSSRAA MQAGIDKLAD AVGLTLGPRG RNVVLDEFGS PKVVNDGVTI
101 ARAIELPNAM ENAGAALIRE VASKTNDSAG DGTTTASVLA REIIKLGLLT
151 VTSGANPVSV KRGIDKTVQS LIEELEKKAR PVKGRDDIKA VASISAGNDD
201 LIGTMVADAI DKVGPDGVLS IESSSSFETT VDVEEGMEID RGYISPQFVT
251 NPEKLICEFE NARVLVTDQK ITAIKDIIPL LEKTTQLRAP LLIIAEDVTG
301 EALATLVVNK LRGILNVAAI KAPSFGERRK AVLQDIAILT GAEFQANDLG
351 LLIENTSVEQ LGIARKVIIT KDSTQIIADA ASKDEIQARV QQLKKELAET
401 DSVYDTEKLA ERIAKLSGGV AVIKVGAATE TELEDRKLRI EDAKNATFAA
451 IEEGIVPGGG AALVHLSTCV PAIKDKLEDP EERIGADIVQ KALVAPASLI
501 AQNAGMEGEV VVEKVKNSEW EIGYNAMTDT YENLLAAGVI DPAKVTRCAL
551 QNAASVAGMV LTTQAIVVEK AKPKAPAAAA PEGLTI
```

Spot No.: A9

Protein name: Chaperonin-60alpha

Peptide sequences:

K.EIAFDQSSR.A;K.LADAVGLTLGPR.G;R.AIELPNAMENAGAALIR.E;R.GYISPQFVTNPEK.L;K.LICEFENAR.V;K.AVLQDIAILTGAEFQANDLGLLIENTSVEQLGIAR.;K.DSTQIIADAASKDEIQAR.V

Accession No.: Gohir.A13G232000.1.p

Mascot score: 590 **Sequence coverage %:** 19

Calculated Mr: 62143 **Calculated *p*I:** 5.15

PFF Searched Score:

Ions score is -10*Log(P), where P is the probability that the observed match is a random event.
Individual ions scores > 34 indicate identity or extensive homology (p<0.05).
Protein scores are derived from ions scores as a non-probabilistic basis for ranking protein hits.

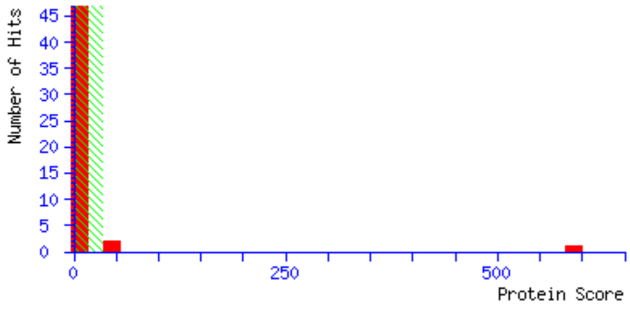

Matched peptide sequences: shown in Bold Red:

1 MASANALSSA SILCSPNKGS LRRKGNQRQN QRVNYRQGNN RFGVKACAK**E**
51 **IAFDQSSR**AA MQAGIDK**LAD** **AVGLTLGPR**G RNVVLDEFGS PKVVNDGVTI
101 AR**AIELPNAM** **ENAGAALIR**E VASKTNDSAG DGTTTASVLA REIIKLGLLT
151 VTSGANPVSV KRGIDKTVQS LIEELEKKAR PVKGRDDIKA VASISAGNDD
201 LIGTMVADAI DKVGPDGVLS IESSSSFETT VDVEEGMEID R**GYISPQFVT**
251 **NPEKLICEFE** **NAR**VLVTDQK ITAIKDIIPL LEKTTQLRAP LLIIAEDVTG
301 EALATLVVNK LRGILNVAAI KAPSFGERRK **AVLQDIAILT** **GAEFQANDLG**
351 **LLIENTSVEQ** **LGIAR**KVIIT K**DSTQIIADA** **ASKDEIQAR**V QQLKKELAET
401 DSVYDTEKLA ERIAKLSGGV AVIKVGAATE TELEDRKLRI EDAKNATFAA
451 IEEGIVPGGG AALVHLSTCV PAIKDKLEDP EERIGADIVQ KALVAPASLI
501 AQNAGMEGEV VVEKVKNSEW EIGYNAMTDT YENLLAAGVI DPAKVTRCAL
551 QNAASVAGMV LTTQAIVVEK AKPKAPAAAA PEGLTI

Spot No.: A12

Protein name: Protein disulfide-isomerase

Peptide sequences:

K.VDANEESNKDLANEYEVR.G;K.LDATANDIMDPNFDVR.G;R.GYPTVYFR.S;R.SADGNISAYEGER.T

Accession No.: Gohir.D05G270900.1.p

Mascot score: 162 **Sequence coverage %:** 11

Calculated Mr: 55800 **Calculated *p*I:** 4.89

PFF Searched Score:

Ions score is -10*Log(P), where P is the probability that the observed match is a random event.
Individual ions scores > 34 indicate identity or extensive homology (p<0.05).
Protein scores are derived from ions scores as a non-probabilistic basis for ranking protein hits.

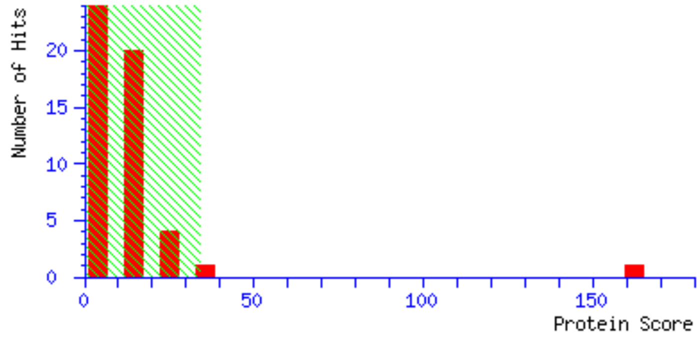

Matched peptide sequences: shown in Bold Red:

```
  1 MGRIVSVWLA FAVIACSLTA ISAEESGESK EFVLTLDQSN FTDTVSKHDF
 51 IVVEFYAPWC GHCKNLSPEY EKAASMLSKH DPPILLAKVD ANEESNKDLA
101 NEYEVRGFPT LKILRNGGKN VQEYKGPREA DGIVEYLKKQ SGPASAEIKS
151 AEDASSFIDE KKIVIVGVFP KFSGQEFDNY MALAEKLRSD YEFGHSLDAK
201 YLPRGGSSVT GPVVRLFKPF DELFVDFKDF NVEALEKFVE ESSIPLVTLF
251 NNDPSNHPFV IKFYNSPLVK AMLFANLSNE GVDSLKYKFR EVAEQYKGQG
301 IGFLLGDLEA SQAAFQYFGV QESQVPLIII LENDGKKYLK PNLEADHIAP
351 WVKDYKEGKV PPYVKSEPIP VENNEPVKVV VADTFDDMVF KSGKNVLLEF
401 YAPWCGHCQK LAPILEEVAV HYENDAKVLI AKLDATANDI MDPNFDVRGY
451 PTVYFRSADG NISAYEGERT KEDIIDFIEK NREKTAHQEA LKDEL
```

Spot No.: A13

Protein name: Chaperonin Cpn60/TCP-1 family

Peptide sequences:

K.SAENSLYVVEGMQFDR.G;R.GYISPYFVTDSEK.M;R.DLINILEDAIR.S;K.AAVEEGIVVGGGCTLLR.L

Accession No.: Gohir.D10G200300.1.p

Mascot score: 278 **Sequence coverage %:** 9

Calculated Mr: 64709 **Calculated *p*I:** 5.72

PFF Searched Score:

Ions score is -10*Log(P), where P is the probability that the observed match is a random event.
Individual ions scores > 34 indicate identity or extensive homology (p<0.05).
Protein scores are derived from ions scores as a non-probabilistic basis for ranking protein hits.

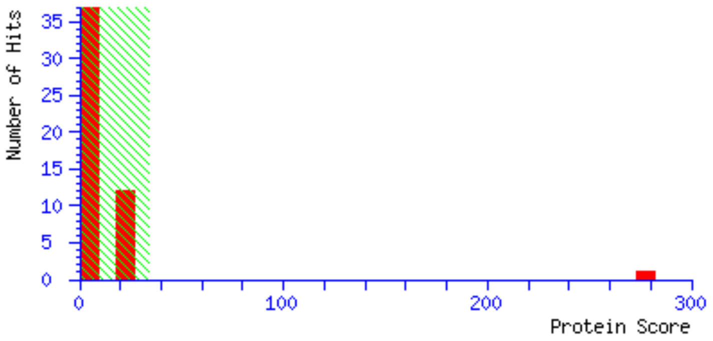

Matched peptide sequences: shown in Bold Red:

1 MASTFTAMSS VGSFVAPNGL VMDKKLSSSS NRLSSLASIS TSSFVSRRNV
51 VLRRSRLPKI SAAKELHFNK DGSAIKRLQT GVNKLADLVG VTLGPKGRNV
101 VLESKYGSPK IVNDGVTVAK EVELEDPVEN IGAKLVRQAA AKTNDLAGDG
151 TTTSVVLAQG LIAEGVKVVA AGANPVLITR GIEKTSRALV SELKAISKEV
201 EDSELADVAA VSAGNNNEVG NMIAEAMSKV GRKGVVTLEE GK**SAENSLYV**
251 **VEGMQFDRGY** **ISPYFVTDSE** **K**MAVEYENCK LLLVDKKITN AR**DLINILED**
301 **AIR**SGYPILI IAEDIEQEAL ATLVVNKLRG ALKIAALKAP GFGERKSQYL
351 DDIAILTGGT VIRDEVGLSL DKASKEVLGH ASKVVLTKDT TTIVGDGSTQ
401 EAVNKRVVQI KNLIEAAEQD YEKEKLNERI AKLSGGVAVI QVGAQTETEL
451 KEKKLRVEDA LNATK**AAVEE** **GIVVGGGCTL** **LR**LASKVDAI KDSLDNDEEK
501 VGADIVKRAL SYPLKLIAKN AGVNGSVVSE KVLSNDNPRY GFNAATGNYE
551 DLMSAGIIDP TKVVRCCLEH AASVAKTFLM SDCVVVEIKE PEPVPAGNPM
601 DNSGYGY

Spot No.: A15

Protein name: Chaperonin Cpn60/TCP-1 family

Peptide sequences:

K.SAENSLYVVEGMQFDR.G;R.GYISPYFVTDSEK.M;R.DLINILEDAIR.S;R.KSQYLDDIAILTGGTVIR.D;K.SQYLDDIAILTGGTVIRDEVGLSLDK.A;K.AAVEEGIVVGGGCTLLR.L

Accession No.: Gohir.D10G200300.1.p

Mascot score: 469 **Sequence coverage %:** 13

Calculated Mr: 64709 **Calculated *p*I:** 5.72

PFF Searched Score:

Ions score is -10*Log(P), where P is the probability that the observed match is a random event.
Individual ions scores > 34 indicate identity or extensive homology (p<0.05).
Protein scores are derived from ions scores as a non-probabilistic basis for ranking protein hits.

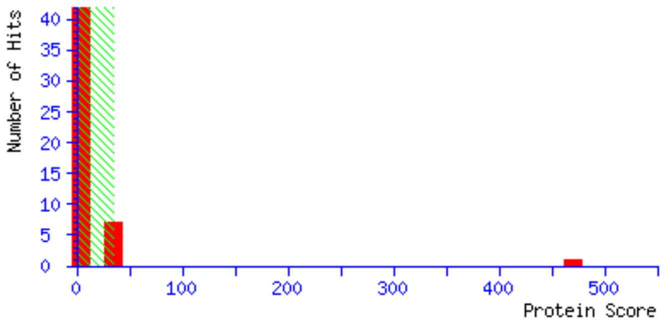

Matched peptide sequences: shown in Bold Red:

```
  1 MASTFTAMSS VGSFVAPNGL VMDKKLSSSS NRLSSLASIS TSSFVSRRNV
 51 VLRRSRLPKI SAAKELHFNK DGSAIKRLQT GVNKLADLVG VTLGPKGRNV
101 VLESKYGSPK IVNDGVTVAK EVELEDPVEN IGAKLVRQAA AKTNDLAGDG
151 TTTSVVLAQG LIAEGVKVVA AGANPVLITR GIEKTSRALV SELKAISKEV
201 EDSELADVAA VSAGNNNEVG NMIAEAMSKV GRKGVVTLEE GKSAENSLYV
251 VEGMQFDRGY ISPYFVTDSE KMAVEYENCK LLLVDKKITN ARDLINILED
301 AIRSGYPILI IAEDIEQEAL ATLVVNKLRG ALKIAALKAP GFGERKSQYL
351 DDIAILTGGT VIRDEVGLSL DKASKEVLGH ASKVVLTKDT TTIVGDGSTQ
401 EAVNKRVVQI KNLIEAAEQD YEKEKLNERI AKLSGGVAVI QVGAQTETEL
451 KEKKLRVEDA LNATKAAVEE GIVVGGGCTL LRLASKVDAI KDSLDNDEEK
501 VGADIVKRAL SYPLKLIAKN AGVNGSVVSE KVLSNDNPRY GFNAATGNYE
551 DLMSAGIIDP TKVVRCCLEH AASVAKTFLM SDCVVVEIKE PEPVPAGNPM
601 DNSGYGY
```

Spot No.: A17

Protein name: ATP synthase subunit beta

Peptide sequences:

K.GQDTAGQQINVTCEVQQLLGNNR.V;K.VVDLLAPYR.R;K.AHGGVSVFGGVGER.T;K.VALVYGQMNEPPGAR.M;R.VGLTALTMAEYFR.D;R.DVNEQDVLLFIDNIFR.F;R.FVQAGSEVSALLGR.M

Accession No.: Gohir.A03G084900.1.p

Mascot score: 653 **Sequence coverage %:** 20%

Calculated Mr: 53611 **Calculated *p*I:** 5.22

PFF Searched Score:

Ions score is -10*Log(P), where P is the probability that the observed match is a random event.
Individual ions scores > 34 indicate identity or extensive homology (p<0.05).
Protein scores are derived from ions scores as a non-probabilistic basis for ranking protein hits.

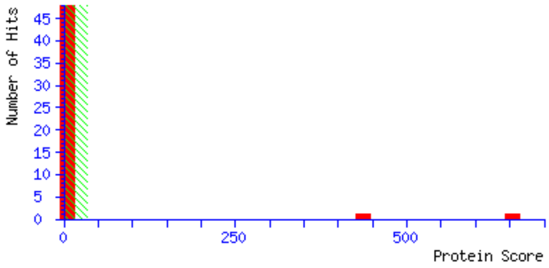

Matched peptide sequences: shown in Bold Red:

1 MKINPTTSVP GVSTLEKENL GRISQIIGPV LDVAFPPGKM PNIYNALVVK
51 **GQDTAGQQIN VTCEVQQLLG NNR**VRAVAMS ATDGLTRGME VIDTGAALSV
101 PVGGATLGRI FNVLGEPVDN LGPVDTRTTS PIHKSAPAFI QLDTKLSIFE
151 TGIK**VVDLLA PYR**RGGKIGL FGGAGVGKTV LIMELINNIA K**AHGGVSVFG**
201 **GVGER**TREGN DLYMEMKESG VINEQNLAES K**VALVYGQMN EPPGAR**MR**VG**
251 **LTALTMAEYF RDVNEQDVLL FIDNIFRFVQ AGSEVSALLG R**MPSAVGYQP
301 TLSTEMGTLQ ERITSTKEGS ITSIQAVYVP ADDLTDPAPA TTFAHLDATT
351 VLSRGLAAKG IYPAVDPLDS TSTMLQPRIV GEEHYETAQR VKQTLQRYKE
401 LQDIIAILGL DELSEEDRLT VARARKIERF LSQPFFVAEV FTGSPGKYVG
451 LAETIRGFKL ILSGELDGLP EQAFYLVGNI DEATAKATNL EMESKLKK

Spot No.: A20

Protein name: Ribulose bisphosphate carboxylase

Peptide sequences:

K.GLAYDISDDQQDITR.G;K.MCALFINDLDAGAGR.M;R.VPIIVTGNDFSTLYAPLIR.D;K.FYWAPTR.D;K.LVDTFPGQSIDFFGALR.A;K.LLEYGNMLVAEQENVKR.V;K.AAQQVGVPVPEGCTDPNADNFDPTAR.S

Accession No.: Gohir.A06G166200.1.p

Mascot score: 707 **Sequence coverage %:** 24

Calculated Mr: 51953 **Calculated *p*I:** 5.37

PFF Searched Score:

Ions score is -10*Log(P), where P is the probability that the observed match is a random event.
Individual ions scores > 34 indicate identity or extensive homology (p<0.05).
Protein scores are derived from ions scores as a non-probabilistic basis for ranking protein hits.

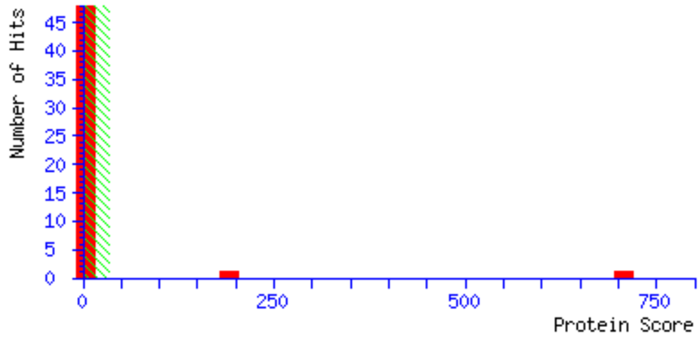

Matched peptide sequences: shown in Bold Red:

```
  1 MAAAISTVAA AAAANRALGV SVPTSAFMGK KVSSRFNNTR QVPSGSFKVM
 51 AAEKEIDEET QTEKDRWKGL AYDISDDQQD ITRGKGMVDS LFQAPMNDGT
101 HYAVMSSYEY ISQGLKTYNL DNNMDGFYIA PAFMDKLVVH ISKNFMSLPN
151 IKVPLILGIW GGKGQGKSFQ CELVFAKMGI NPIMMSAGEL ESGNAGEPAK
201 LIRQRYREAA DIIKKGKMCA LFINDLDAGA GRMGGTTQYT VNNQMVNATL
251 MNIADNPTNV QLPGMYNKEE NPRVPIIVTG NDFSTLYAPL IRDGRMEKFY
301 WAPTRDDRIG VCKGIFRTDG VRDEDIVKLV DTFPGQSIDF FGALRARVYD
351 DEVRKWISEV GVASVGKKLV NSREGPPTFE QPKMTIEKLL EYGNMLVAEQ
401 ENVKRVQLAD KYLSEAALGE ANEDSINRGT FYGKAAQQVG VPVPEGCTDP
451 NADNFDPTAR SDDGTCTYKF
```

Spot No.: A21

Protein name: ATP synthase subunit alpha

Peptide sequences:

K.IVNTGTVLQVGDGIAR.I;K.IAQIPVSEAYLGR.V;R.LIESPAP GIISR.R;K.ASSVAQVVTTFQER.G

Accession No.: Gohir.D01G031800.1.p

Mascot score: 323 **Sequence coverage %:** 10

Calculated Mr: 55437 **Calculated *p*I:** 5.25

PFF Searched Score:

Ions score is -10*Log(P), where P is the probability that the observed match is a random event.
Individual ions scores > 34 indicate identity or extensive homology (p<0.05).
Protein scores are derived from ions scores as a non-probabilistic basis for ranking protein hits.

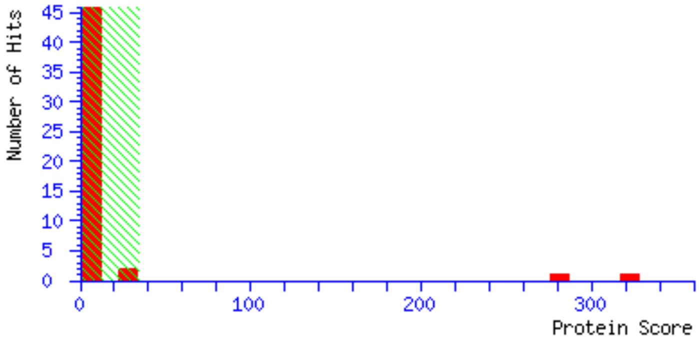

Matched peptide sequences: shown in Bold Red:

```
  1 MVTIRADEIS NIIRERIEQY NREVKIVNTG TVLQVGDGIA RIHGLDEVMA
 51 GELVEFEEGT IGIALNLESN NVGVVLMGDG LMIQEGSSVK ATGKIAQIPV
101 SEAYLGRVIN ALAKPIDGRG EISASESRLI ESPAPGIISR RSVYEPLQTG
151 LIAIDSMIPI GRGQRELIIG DRQTGKTAVA TDTILNQQGQ NVICVYVAIG
201 QKASSVAQVV TTFQERGAME YTIVVAETAD SPATLQYLAP YTGAALAEYF
251 MYRERHTLII YDDLSKQAQA YRQMSLLLRR PPGREAYPGD VFYLHSRLLE
301 RAAKSSSQLG EGSMTALPIV ETQSGDVSAY IPTNVISITD GQIFLSADLF
351 NAGIRPAINV GISVSRVGSA AQIKAMKQVA GKSKLELAQF AELEAFAQFA
401 SDLDKATQNQ LARGQRLREL LKQSQSAPLT VAEQISTIYT GTNGYLDSLE
451 IGQVRKFLVE LRTYLKTNKP QFQEIISSTK TFTEEAETLL KDAIQDQMER
501 FRLQEQL
```

Spot No.: A22

Protein name: ATP synthase subunit alpha

Peptide sequences:

R.ADEISNIIR.E;K.IVNTGTVLQVGDGIAR.I;K.IAQIPVSEAYLGR.V;R.LIESPAPGIISR.R;R.SVYEPLQTGLIAIDSMIPIGR.G;K.ASSVAQVVTTFQER.G;K.QSQSAPLTVAEQISTIYTGTNGYLDSLEIGQVR.K

Accession No.: Gohir.D01G031800.1.p

Mascot score: 696 **Sequence coverage %:** 23

Calculated Mr: 55437 **Calculated *p*I:** 5.25

PFF Searched Score:

Ions score is -10*Log(P), where P is the probability that the observed match is a random event.
Individual ions scores > 34 indicate identity or extensive homology (p<0.05).
Protein scores are derived from ions scores as a non-probabilistic basis for ranking protein hits.

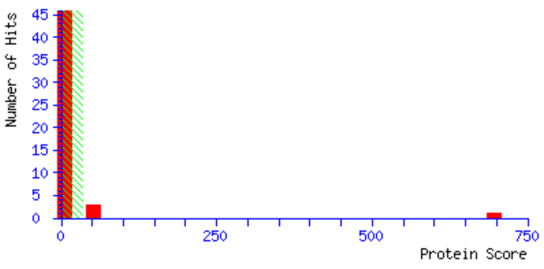

Matched peptide sequences: shown in Bold Red:

1 MVTIRADEIS NIIRERIEQY NREVKIVNTG TVLQVGDGIA RIHGLDEVMA
51 GELVEFEEGT IGIALNLESN NVGVVLMGDG LMIQEGSSVK ATGKIAQIPV
101 SEAYLGRVIN ALAKPIDGRG EISASESRLI ESPAPGIISR RSVYEPLQTG
151 LIAIDSMIPI GRGQRELIIG DRQTGKTAVA TDTILNQQGQ NVICVYVAIG
201 QKASSVAQVV TTFQERGAME YTIVVAETAD SPATLQYLAP YTGAALAEYF
251 MYRERHTLII YDDLSKQAQA YRQMSLLLRR PPGREAYPGD VFYLHSRLLE
301 RAAKSSSQLG EGSMTALPIV ETQSGDVSAY IPTNVISITD GQIFLSADLF
351 NAGIRPAINV GISVSRVGSA AQIKAMKQVA GKSKLELAQF AELEAFAQFA
401 SDLDKATQNQ LARGQRLREL LKQSQSAPLT VAEQISTIYT GTNGYLDSLE
451 IGQVRKFLVE LRTYLKTNKP QFQEIISSTK TFTEEAETLL KDAIQDQMER
501 FRLQEQL

Spot No.: A23

Protein name: ATP synthase subunit alpha

Peptide sequences:

R.ADEISNIIR.E;K.IVNTGTVLQVGDGIAR.I;K.IAQIPVSEAYLGR.V;R.LIESPAPGIISR.R;R.SVYEPLQTGLIAIDSMIPIGR.G;K.ASSVAQVVTTFQER.G

Accession No.: Gohir.D01G031800.1.p

Mascot score: 432 **Sequence coverage %:** 16

Calculated Mr: 55437 **Calculated *p*I:** 5.25

PFF Searched Score:

Ions score is -10*Log(P), where P is the probability that the observed match is a random event.
Individual ions scores > 34 indicate identity or extensive homology (p<0.05).
Protein scores are derived from ions scores as a non-probabilistic basis for ranking protein hits.

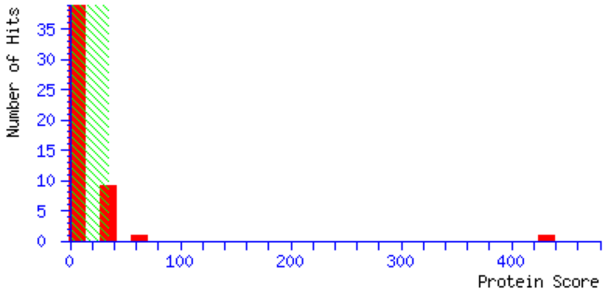

Matched peptide sequences: shown in Bold Red:

```
  1 MVTIRADEIS NIIRERIEQY NREVKIVNTG TVLQVGDGIA RIHGLDEVMA
 51 GELVEFEEGT IGIALNLESN NVGVVLMGDG LMIQEGSSVK ATGKIAQIPV
101 SEAYLGRVIN ALAKPIDGRG EISASESRLI ESPAPGIISR RSVYEPLQTG
151 LIAIDSMIPI GRGQRELIIG DRQTGKTAVA TDTILNQQGQ NVICVYVAIG
201 QKASSVAQVV TTFQERGAME YTIVVAETAD SPATLQYLAP YTGAALAEYF
251 MYRERHTLII YDDLSKQAQA YRQMSLLLRR PPGREAYPGD VFYLHSRLLE
301 RAAKSSSQLG EGSMTALPIV ETQSGDVSAY IPTNVISITD GQIFLSADLF
351 NAGIRPAINV GISVSRVGSA AQIKAMKQVA GKSKLELAQF AELEAFAQFA
401 SDLDKATQNQ LARGQRLREL LKQSQSAPLT VAEQISTIYT GTNGYLDSLE
451 IGQVRKFLVE LRTYLKTNKP QFQEIISSTK TFTEEAETLL KDAIQDQMER
501 FRLQEQL
```

Spot No.: A24

Protein name: ATP synthase subunit beta

Peptide sequences:

K.GQDTAGQQINVTCEVQQLLGNNR.V;R.GMEVIDTGAALSVPVGGATLGR.I;K.AHGGVSVFGGVGER.T;K.VALVYGQMNEPPGAR.M;R.VGLTALTMAEYFR.D;R.DVNEQDVLLFIDNIFR.F;R.FVQAGSEVSALLGR.M

Accession No.: Gohir.A03G084900.1.p

Mascot score: 857 **Sequence coverage %:** 23

Calculated Mr: 53611 **Calculated *p*I:** 5.22

PFF Searched Score:

Ions score is -10*Log(P), where P is the probability that the observed match is a random event.
Individual ions scores > 34 indicate identity or extensive homology (p<0.05).
Protein scores are derived from ions scores as a non-probabilistic basis for ranking protein hits.

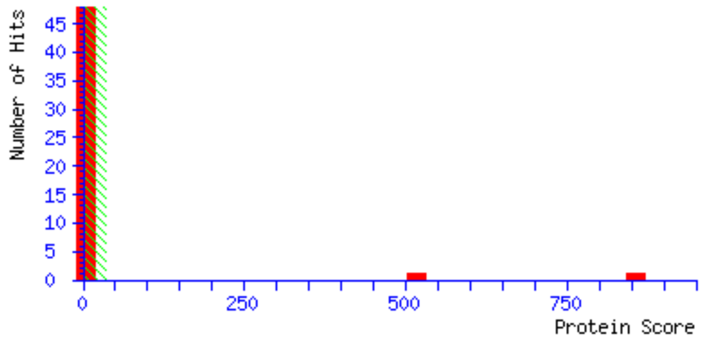

Matched peptide sequences: shown in Bold Red:

1 MKINPTTSVP GVSTLEKENL GRISQIIGPV LDVAFPPGKM PNIYNALVVK

51 GQDTAGQQIN VTCEVQQLLG NNRVRAVAMS ATDGLTRGME VIDTGAALSV

101 PVGGATLGRI FNVLGEPVDN LGPVDTRTTS PIHKSAPAFI QLDTKLSIFE

151 TGIKVVDLLA PYRRGGKIGL FGGAGVGKTV LIMELINNIA KAHGGVSVFG

201 GVGERTREGN DLYMEMKESG VINEQNLAES KVALVYGQMN EPPGARMRVG

251 LTALTMAEYF RDVNEQDVLL FIDNIFRFVQ AGSEVSALLG RMPSAVGYQP

301 TLSTEMGTLQ ERITSTKEGS ITSIQAVYVP ADDLTDPAPA TTFAHLDATT

351 VLSRGLAAKG IYPAVDPLDS TSTMLQPRIV GEEHYETAQR VKQTLQRYKE

401 LQDIIAILGL DELSEEDRLT VARARKIERF LSQPFFVAEV FTGSPGKYVG

451 LAETIRGFKL ILSGELDGLP EQAFYLVGNI DEATAKATNL EMESKLKK

Spot No.: B2

Protein name: ATP synthase subunit beta

Peptide sequences:

R.GMEVIDTGAALSVPVGGATLGR.I;K.VVDLLAPYR.R;K.AHGGVSVFGGVGER.T;K.VALVYGQMNEPPGAR.M;R.VGLTALTMAEYFR.D;R.DVNEQDVLLFIDNIFR.F;R.FVQAGSEVSALLGR.M

Accession No.: Gohir.A03G084900.1.p

Mascot score: 547 **Sequence coverage %:** 20

Calculated Mr: 53611 **Calculated *p*I:** 5.22

PFF Searched Score:

Ions score is -10*Log(P), where P is the probability that the observed match is a random event.
Individual ions scores > 34 indicate identity or extensive homology (p<0.05).
Protein scores are derived from ions scores as a non-probabilistic basis for ranking protein hits.

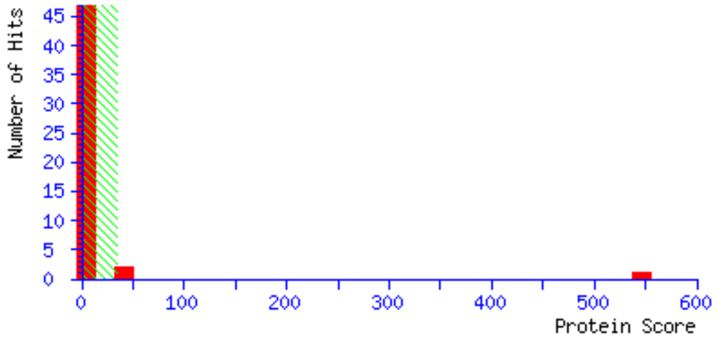

Matched peptide sequences: shown in Bold Red:

```
  1 MKINPTTSVP GVSTLEKENL GRISQIIGPV LDVAFPPGKM PNIYNALVVK
 51 GQDTAGQQIN VTCEVQQLLG NNRVRAVAMS ATDGLTRGME VIDTGAALSV
101 PVGGATLGRI FNVLGEPVDN LGPVDTRTTS PIHKSAPAFI QLDTKLSIFE
151 TGIKVVDLLA PYRRGGKIGL FGGAGVGKTV LIMELINNIA KAHGGVSVFG
201 GVGERTREGN DLYMEMKESG VINEQNLAES KVALVYGQMN EPPGARMRVG
251 LTALTMAEYF RDVNEQDVLL FIDNIFRFVQ AGSEVSALLG RMPSAVGYQP
301 TLSTEMGTLQ ERITSTKEGS ITSIQAVYVP ADDLTDPAPA TTFAHLDATT
351 VLSRGLAAKG IYPAVDPLDS TSTMLQPRIV GEEHYETAQR VKQTLQRYKE
401 LQDIIAILGL DELSEEDRLT VARARKIERF LSQPFFVAEV FTGSPGKYVG
451 LAETIRGFKL ILSGELDGLP EQAFYLVGNI DEATAKATNL EMESKLKK
```

Spot No.: B3

Protein name: tubulin alpha-3

Peptide sequences:

R.AVFVDLEPTVIDEVR.T;R.LISQTISSLTTSLR.F;R.FDGAINVDITEFQTNLVPYPR.I;R.AVCMISNNTAVAEVFAR.I;R.AFVHWYVGEGMEEGEFSEAR.E

Accession No.: Gohir.A11G076100.1

Mascot score: 369 **Sequence coverage %:** 19

Calculated Mr: 50264 **Calculated *p*I:** 5.01

PFF Searched Score:

Ions score is -10*Log(P), where P is the probability that the observed match is a random event.
Individual ions scores > 33 indicate identity or extensive homology (p<0.05).
Protein scores are derived from ions scores as a non-probabilistic basis for ranking protein hits.

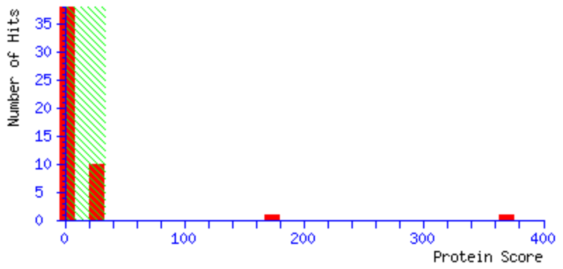

Matched peptide sequences: shown in Bold Red:

1 MREIISVHIG QAGIQVGNSC WELYCLEHVI HPDGTMPSDT SMGVAHDAFN
51 TFFSETGSGK HVPR**AVFVDL EPTVIDEVR**T GPYRQLFHPE QLISGKEDAA
101 NNFARGHYTI GKEIVDLCLD RVRKLADNCT GLQGFMVFNA VGGGTGSGLG
151 SLLLERLSVD YGKKSKLGFT IYPSPQVSTA VVEPYNSVLS THSLLEHTDV
201 AVLLDNEAIY DICRRSLDIE RPTYTNLNR**L ISQTISSLTT SLRFDGAINV**
251 **DITEFQTNLV PYPR**IHFMLS SYAPVISAEK AYHEQISVPE ITNAVFEPSS
301 MMAKCDPRHG KYMACCLMYR GDVVPKDVNA AVATIKTKRT VQFVDWCPTG
351 FKCGINYQPP AVVPGGDLAK VQR**AVCMISN NTAVAEVFAR** IDHKFDLMYS
401 KR**AFVHWYVG EGMEEGEFSE AR**EDLAALEK DYEEVGAEGG DDEEEGEEY

Spot No.: B4

Protein name: Ribulose bisphosphate carboxylase

Peptide sequences:

K.GLAYDISDDQQDITR.G;K.MCALFINDLDAGAGR.M;R.VPIIVTGNDFSTLYAPLIR.D;K.FYWAPTR.D;K.LVDTFPGQSIDFFGALR.A;K.LLEYGNMLVAEQENVKR.V

Accession No.: Gohir.A06G166200.1.p

Mascot score: 604 **Sequence coverage %:** 19

Calculated Mr: 51953 **Calculated *p*I:** 5.37

PFF Searched Score:

Ions score is -10*Log(P), where P is the probability that the observed match is a random event.
Individual ions scores > 34 indicate identity or extensive homology (p<0.05).
Protein scores are derived from ions scores as a non-probabilistic basis for ranking protein hits.

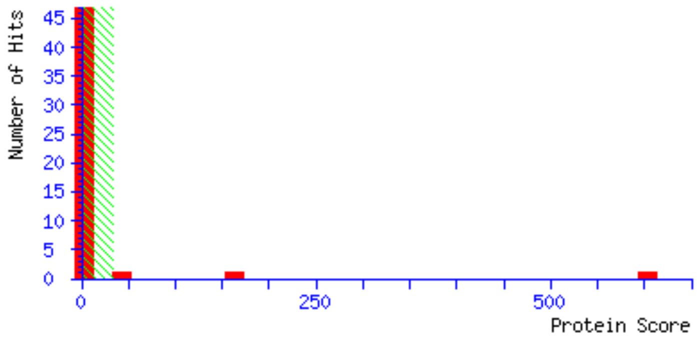

Matched peptide sequences: shown in Bold Red:

1 MAAAISTVAA AAAANRALGV SVPTSAFMGK KVSSRFNNTR QVPSGSFKVM
51 AAEKEIDEET QTEKDRWK**GL** **AYDISDDQQD** **ITR**GKGMVDS LFQAPMNDGT
101 HYAVMSSYEY ISQGLKTYNL DNNMDGFYIA PAFMDKLVVH ISKNFMSLPN
151 IKVPLILGIW GGKGQGKSFQ CELVFAKMGI NPIMMSAGEL ESGNAGEPAK
201 LIRQRYREAA DIIKKGK**MCA** **LFINDLDAGA** **GR**MGGTTQYT VNNQMVNATL
251 MNIADNPTNV QLPGMYNKEE NPR**VPIIVTG** **NDFSTLYAPL** **IR**DGRMEK**FY**
301 **WAPTR**DDRIG VCKGIFRTDG VRDEDIVK**LV** **DTFPGQSIDF** **FGALR**ARVYD
351 DEVRKWISEV GVASVGKKLV NSREGPPTFE QPKMTIEK**LL** **EYGNMLVAEQ**
401 **ENVKR**VQLAD KYLSEAALGE ANEDSINRGT FYGKAAQQVG VPVPEGCTDP
451 NADNFDPTAR SDDGTCTYKF

Spot No.: B5

Protein name: Ribulose bisphosphate carboxylase

Peptide sequences:

K.GLAYDISDDQQDITR.G;K.MCALFINDLDAGAGR.M;R.VPIIVTGNDFSTLYAPLIR.D;K.FYWAPTR.D;K.LVDTFPGQSIDFFGALR.A;K.LLEYGNMLVAEQENVKR.V

Accession No.: Gohir.D06G171700.1

Mascot score: 804 **Sequence coverage %:**24

Calculated Mr: 51953 **Calculated *p*I:** 5.37

PFF Searched Score:

Ions score is -10*Log(P), where P is the probability that the observed match is a random event.
Individual ions scores > 34 indicate identity or extensive homology (p<0.05).
Protein scores are derived from ions scores as a non-probabilistic basis for ranking protein hits.

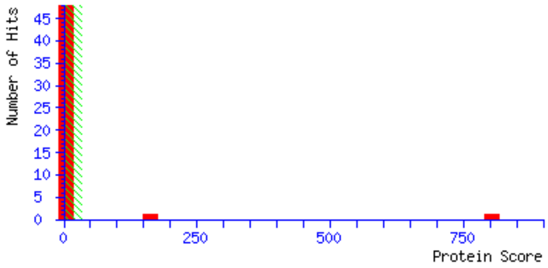

Matched peptide sequences: shown in Bold Red:

```
  1 MAAAISTVAA AAAANRALGV SVPTSAFMGK KVSSRFNNTR QVPSGSFKVM
 51 AAEKEIDEET QTEKDRWKGL AYDISDDQQD ITRGKGMVDS LFQAPMNDGT
101 HYAVMSSYEY ISQGLKTYNL DNNMDGFYIA PAFMDKLVVH ISKNFMSLPN
151 IKVPLILGIW GGKGQGKSFQ CELVFAKMGI NPIMMSAGEL ESGNAGEPAK
201 LIRQRYREAA DIIKKGKMCA LFINDLDAGA GRMGGTTQYT VNNQMVNATL
251 MNIADNPTNV QLPGMYNKEE NPRVPIIVTG NDFSTLYAPL IRDGRMEKFY
301 WAPTRDDRIG VCKGIFRTDG VRDEDIVKLV DTFPGQSIDF FGALRARVYD
351 DEVRKWISEV GVASVGKKLV NSREGPPTFE QPKMTIEKLL EYGNMLVAEQ
401 ENVKRVQLAD KYLSEAALGE ANEDSINRGT FYGKAAQQVG VPVPEGCTDP
451 NADNFDPTAR SDDGTCTYKF
```

Spot No.: B6

Protein name: Rubisco activase

Peptide sequences:

K.GLAYDISDDQQDITR.G;R.VPIIVTGNDFSTLYAPLIR.D;K.FYWAPTR.E;R.IGVCTGIFR.T;K.LVDTFPGQSIDFFGALR.A;K.YLSEAALGNANDDAIKR.G

Accession No.: Gohir.A10G221700.1.p

Mascot score: 476 **Sequence coverage %:**19

Calculated Mr: 48189 **Calculated *p*I:** 5.40

PFF Searched Score:

Ions score is -10*Log(P), where P is the probability that the observed match is a random event.
Individual ions scores > 34 indicate identity or extensive homology (p<0.05).
Protein scores are derived from ions scores as a non-probabilistic basis for ranking protein hits.

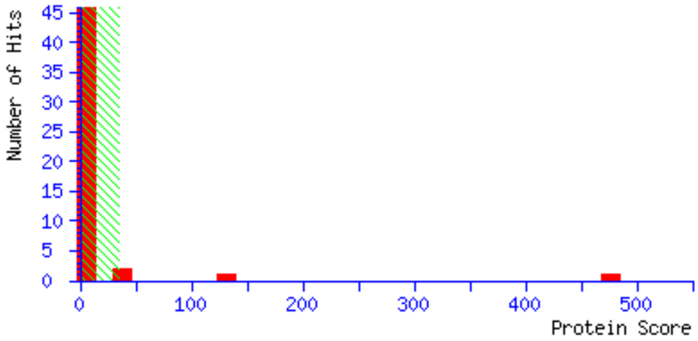

Matched peptide sequences: shown in Bold Red:

```
  1 MAAAVSTIGA VNRAPLSLNG SGAGASAPSS AFMGNSLKKV SARFNNNGKA
 51 PVGSFMIVAA KEIDEDTQTD QDRWKGLAYD ISDDQQDITR GKGMVDSLFQ
101 APMNDGTHYA VMSSYEYISQ GLRTYDLDNN MDGFYIAPAF MDKLVVHITK
151 NYMTLPNIKV PLILGIWGGK GQGKSFQCEL VFAKMGINPI MMSAGELESG
201 NAGEPAKLIR QRYREAADII KKGKMCCLFI NDLDAGAGRM GGTTQYTVNN
251 QMVNATLMNI ADNPTNVQLP GMYNKEENPR VPIIVTGNDF STLYAPLIRD
301 GRMEKFYWAP TREDRIGVCT GIFRTDNVPV DDIVKLVDTF PGQSIDFFGA
351 LRARVYDDEV RKWIGEVGVN SVGKKLVNSR EGPPSFEQPT MTIEKLLEYG
401 NMLVAEQENV KRVQLADKYL SEAALGNAND DAIKRGAF
```

Spot No.: B7

Protein name: Ribulose bisphosphate carboxylase

Peptide sequences:

K.GLAYDISDDQQDITR.G;K.MCALFINDLDAGAGR.M;R.VPIIVTGNDFSTLYAPLIR.D;K.FYWAPTR.D;K.LVDTFPGQSIDFFGALR.A;K.LLEYGNMLVAEQENVKR.V;K.AAQQVGVPVPEGCTDPNADNFDPTAR.S

Accession No.: Gohir.A06G166200.1.p

Mascot score: 849 **Sequence coverage %:**24

Calculated Mr: 51953 **Calculated** ***p*I:** 5.37

PFF Searched Score:

Ions score is -10*Log(P), where P is the probability that the observed match is a random event.
Individual ions scores > 34 indicate identity or extensive homology (p<0.05).
Protein scores are derived from ions scores as a non-probabilistic basis for ranking protein hits.

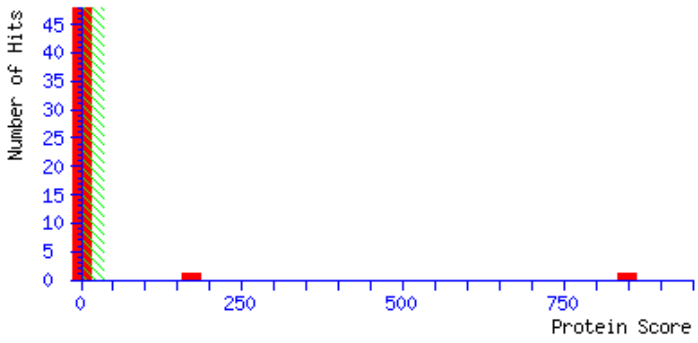

Matched peptide sequences: shown in Bold Red:

```
  1 MAAAISTVAA AAAANRALGV SVPTSAFMGK KVSSRFNNTR QVPSGSFKVM
 51 AAEKEIDEET QTEKDRWKGL AYDISDDQQD ITRGKGMVDS LFQAPMNDGT
101 HYAVMSSYEY ISQGLKTYNL DNNMDGFYIA PAFMDKLVVH ISKNFMSLPN
151 IKVPLILGIW GGKGQGKSFQ CELVFAKMGI NPIMMSAGEL ESGNAGEPAK
201 LIRQRYREAA DIIKKGKMCA LFINDLDAGA GRMGGTTQYT VNNQMVNATL
251 MNIADNPTNV QLPGMYNKEE NPRVPIIVTG NDFSTLYAPL IRDGRMEKFY
301 WAPTRDDRIG VCKGIFRTDG VRDEDIVKLV DTFPGQSIDF FGALRARVYD
351 DEVRKWISEV GVASVGKKLV NSREGPPTFE QPKMTIEKLL EYGNMLVAEQ
401 ENVKRVQLAD KYLSEAALGE ANEDSINRGT FYGKAAQQVG VPVPEGCTDP
451 NADNFDPTAR SDDGTCTYKF
```

Spot No.: B8

Protein name: **Ribulose bisphosphate carboxylase**

Peptide sequences:

K.GLAYDISDDQQDITR.G;K.MCALFINDLDAGAGR.M;R.VPIIVTGNDFSTLYAPLIR.D;K.FYWAPTR.D;K.LVDTFPGQSIDFFGALR.A;K.LLEYGNMLVAEQENVKR.V;K.AAQQVGVPVPEGCTDPNADNFDPTAR.S

Accession No.: Gohir.A06G166200.1.p

Mascot score: 819 **Sequence coverage %:**24

Calculated Mr: 51953 **Calculated *p*I:** 5.37

PFF Searched Score:

Ions score is -10*Log(P), where P is the probability that the observed match is a random event.
Individual ions scores > 34 indicate identity or extensive homology (p<0.05).
Protein scores are derived from ions scores as a non-probabilistic basis for ranking protein hits.

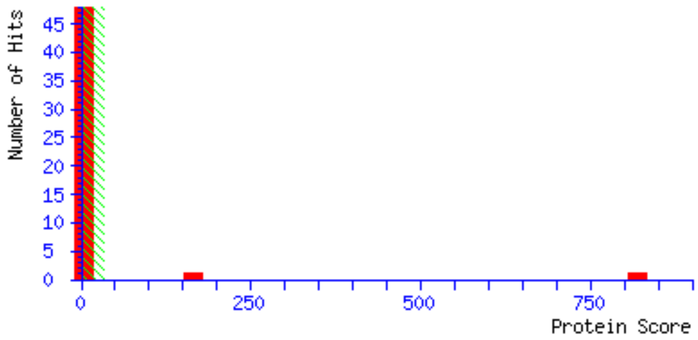

Matched peptide sequences: shown in Bold Red:

1 MAAAISTVAA AAAANRALGV SVPTSAFMGK KVSSRFNNTR QVPSGSFKVM
51 AAEKEIDEET QTEKDRWK**GL** **AYDISDDQQD** **ITR**GKGMVDS LFQAPMNDGT
101 HYAVMSSYEY ISQGLKTYNL DNNMDGFYIA PAFMDKLVVH ISKNFMSLPN
151 IKVPLILGIW GGKGQGKSFQ CELVFAKMGI NPIMMSAGEL ESGNAGEPAK
201 LIRQRYREAA DIIKKGK**MCA** **LFINDLDAGA** **GR**MGGTTQYT VNNQMVNATL
251 MNIADNPTNV QLPGMYNKEE NPR**VPIIVTG** **NDFSTLYAPL** **IR**DGRMEK**FY**
301 **WAPTR**DDRIG VCKGIFRTDG VRDEDIVK**LV** **DTFPGQSIDF** **FGALR**ARVYD
351 DEVRKWISEV GVASVGKKLV NSREGPPTFE QPKMTIEK**LL** **EYGNMLVAEQ**
401 **ENVKR**VQLAD KYLSEAALGE ANEDSINRGT FYGK**AAQQVG** **VPVPEGCTDP**
451 **NADNFDPTAR** SDDGTCTYKF

Spot No.: B10

Protein name: S-adenosylmethionine synthetase 2

Peptide sequences:

K.VLVNIEQQSPDIAQGVHGHFTK.R;K.TIFHLNPSGR.F;R.FVIG GPHGDAGLTGR.K

Accession No.: Gohir.D07G135100.1.p

Mascot score: 144 **Sequence coverage %:**11

Calculated Mr: 43468 **Calculated *p*I:** 5.49

PFF Searched Score:

Ions score is -10*Log(P), where P is the probability that the observed match is a random event.
Individual ions scores > 34 indicate identity or extensive homology (p<0.05).
Protein scores are derived from ions scores as a non-probabilistic basis for ranking protein hits.

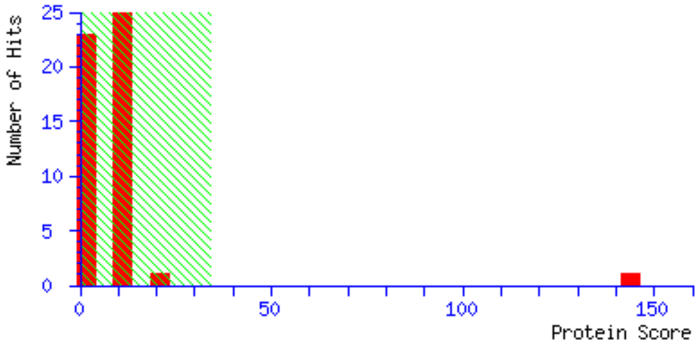

Matched peptide sequences: shown in Bold Red:

```
  1 METFLFTSES VNEGHPDKLC DQISDAVLDA CLAQDPDSKV ACETCSKTNM
 51 VMVFGEITTK ANVDYEKIVR DTCRNIGFVS DDVGLDADNC KVLVNIEQQS
101 PDIAQGVHGH FTKRPEDIGA GDQGHMFGYA TDETPEFMPL SHVLATKLGA
151 RLTEVRKNGT CPWLRPDGKT QVTVEYYDDN GAMVPVRVHT VLISTQHDET
201 VTNDEIAADL KEHVIKPVIP EKYLDGKTIF HLNPSGRFVI GGPHGDAGLT
251 GRKIIIDTYG GWGAHGGGAF SGKDPTKVDR SGAYIVRQAA KSIVANGLAR
301 RCLVQVSYAI GVPEPLSVFV NSYGTGKIPD KEILQIVKEN FDFRPGMITI
351 DLDLKRGGNG RFLKTAAYGH FGRDDPDFTW EVVKPLKWEK PQS
```

Spot No.: B17

Protein name: Actin 7

Peptide sequences:

R.AVFPSIVGR.P;R.AVFPSIVGRPR.H;K.IWHHTFYNELR.V;R.VAPEEHPVLLTEAPLNPK.A;K.NYELPDGQVITIGAER.F

Accession No.: Gohir.A03G105800.1.p

Mascot score: 389 **Sequence coverage %:**14

Calculated Mr: 41913 **Calculated *p*I:** 5.31

PFF Searched Score:

Ions score is -10*Log(P), where P is the probability that the observed match is a random event.
Individual ions scores > 35 indicate identity or extensive homology (p<0.05).
Protein scores are derived from ions scores as a non-probabilistic basis for ranking protein hits.

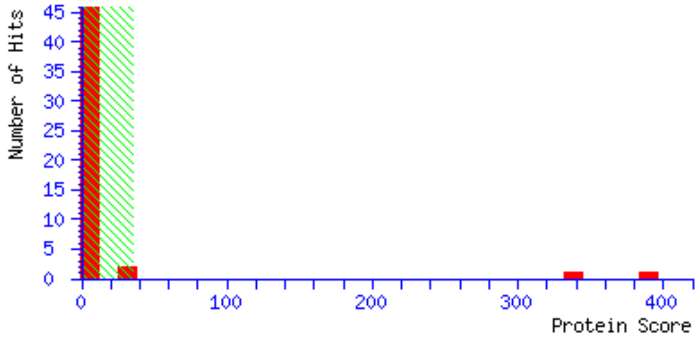

Matched peptide sequences: shown in Bold Red:

1 MADAEDIQPL VCDNGTGMVK AGFAGDDAPR **AVFPSIVGRP R**HTGVMVGMG
51 QKDAYVGDEA QSKRGILTLK YPIEHGIVSN WDDMEK**IWHH TFYNELRVAP**
101 **EEHPVLLTEA PLNPK**ANREK MTQIMFETFN VPAMYVAIQA VLSLYASGRT
151 TGIVLDSGDG VSHTVPIYEG YALPHAILRL DLAGRDLTDS LMKILTERGY
201 MFTTTAEREI VRDMKEKLAY VALDYEQELE TAKSSSSVEK **NYELPDGQVI**
251 **TIGAER**FRCP EVLFQPSLIG MEAAGIHETT YNSIMKCDVD IRKDLYGNIV
301 LSGGSTMFPG IADRMSKEIT ALAPSSMKIK VVAPPERKYS VWIGGSILAS
351 LSTFQQMWIS KGEYDESGPS IVHRKCF

Spot No.: B18

Protein name: RAB GTPase homolog E1B

Peptide sequences:

K.KYDEIDAAPEER.A;R.GITINTATVEYETENR.H;R.QTDLPFLLAVEDVFSITGR.G

Accession No.: Gohir.A03G057300.1.p

Mascot score: 158 **Sequence coverage %:** 9

Calculated Mr: 52793 **Calculated *p*I:** 6.21

PFF Searched Score:

Ions score is -10*Log(P), where P is the probability that the observed match is a random event.
Individual ions scores > 34 indicate identity or extensive homology (p<0.05).
Protein scores are derived from ions scores as a non-probabilistic basis for ranking protein hits.

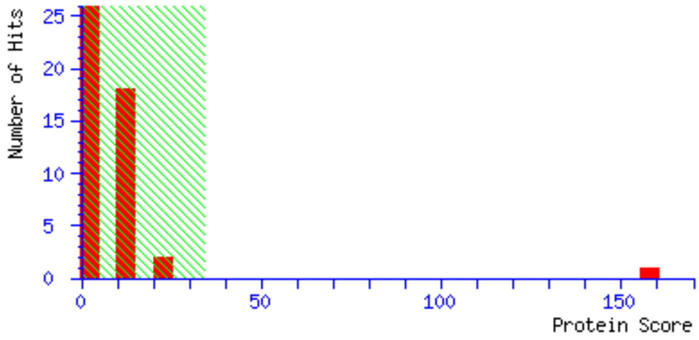

Matched peptide sequences: shown in Bold Red:

```
  1 MAISSTAAAT AASSKLRYPH ASPFPTHSST TTSAFFSSIP SKLTPTHLSS
 51 SFLPPFLTTV ATTYVFPRRR DSFTVKAARG KFERKKPHVN IGTIGHVDHG
101 KTTLTAALTM ALASMGNSAP KKYDEIDAAP EERARGITIN TATVEYETEN
151 RHYAHVDCPG HADYVKNMIT GAAQMDGAIL VVSGADGPMP QTKEHILLAK
201 QVGVPNVVVF LNKQDQVDDE ELLQLVELEV RELLSSYEFP GDDVPIISGS
251 ALLALEALMA KPSIPRGENQ WVDKIYELMD AVDSYIPVPQ RQTDLPFLLA
301 VEDVFSITGR GTVATGRIER GTVKVGETVD IVGLKDTRNV TVTGVEMFQK
351 TLDDAMAGDN VGLLLRGVQK ADIQRGMVLA KPGTITPHTK FSAIVYVLKK
401 EEGGRHSPFF AGYRPQFYMR TTDVTGRVAS IMNDKDEESK MVMPGDRVKM
451 VVELIMPVAC EQGMRFAIRE GGKTVGAGVI QSIIE
```

Spot No.: B19

Protein name: Glutamine synthetase

Peptide sequences:

K.WNYDGSSTGQAPGEDSEVILYPQAIFKDPFR.G;R.GGNNILVICDAYTPAGEPIPTNKR.H;R.HKEHISAYGEGNER.R;K.HETASINTFSWGVANR.G;R.RPASNMDPYVVTALLAETTLLYEPTLEAEALAAQK.I

Accession No.: Gohir.D02G032300.1.p

Mascot score: 426 **Sequence coverage %:** 27

Calculated Mr: 47707 **Calculated *p*I:** 7.08

PFF Searched Score:

Ions score is -10*Log(P), where P is the probability that the observed match is a random event.
Individual ions scores > 34 indicate identity or extensive homology (p<0.05).
Protein scores are derived from ions scores as a non-probabilistic basis for ranking protein hits.

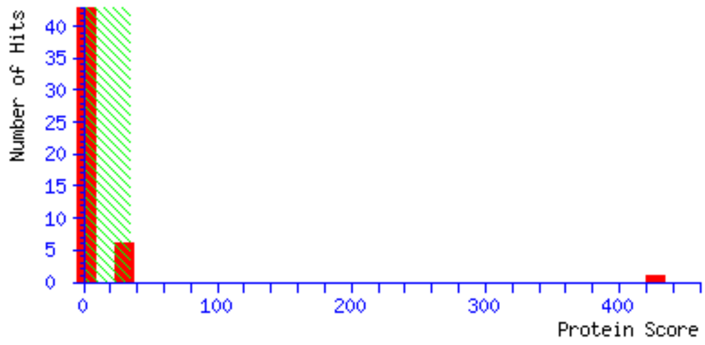

Matched peptide sequences: shown in Bold Red:

```
  1 MAQLLAPSTQ WQMTLPKTST YGSPIATKMW SSLVLKQNKK GAAKSSGKFK
 51 VVALSENSTV NRLENLLNMD VTPYTDKIIA EYVWIGGSGL DMRSKSRTIS
101 KPVKHPSELP KWNYDGSSTG QAPGEDSEVI LYPQAIFKDP FRGGNNILVI
151 CDAYTPAGEP IPTNKRHRAA EIFSNKKVVD EVPWFGIEQE YTLLQQNVKW
201 PLGWPVGGYP GPQGPYYCAA GADKSFGRDI SDAHYKACLY AGINISGTNG
251 EVMPGQWEYQ VGPSVGIEAG DHIWCSRYIL ERITEQAGVV LSLDPKPIEG
301 DWNGAGCHTN YSTKSMREDG GFEVIKKAIL NLSLRHKEHI SAYGEGNERR
351 LTGKHETASI NTFSWGVANR GCSIRVGRDT EKNGKGYLED RRPASNMDPY
401 VVTALLAETT LLYEPTLEAE ALAAQKIALN V
```

Spot No.: B20

Protein name: Rubisco activase

Peptide sequences:

K.GLAYDISDDQQDITR.G;R.VPIIVTGNDFSTLYAPLIR.D;K.FYWAPTR.E;R.IGVCTGIFR.T;K.LVDTFPGQSIDFFGALR.A;K.YLSEAALGNANDDAIKR.G

Accession No.: Gohir.A10G221700.1.p

Mascot score: 523 **Sequence coverage %:** 19

Calculated Mr: 48189 **Calculated *p*I:** 5.40

PFF Searched Score:

Ions score is -10*Log(P), where P is the probability that the observed match is a random event.
Individual ions scores > 34 indicate identity or extensive homology (p<0.05).
Protein scores are derived from ions scores as a non-probabilistic basis for ranking protein hits.

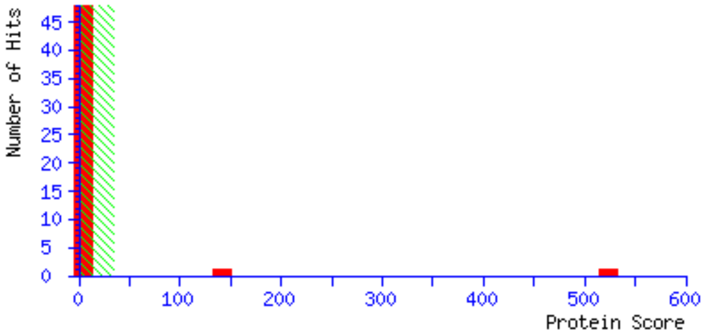

Matched peptide sequences: shown in Bold Red:

1 MAAAVSTIGA VNRAPLSLNG SGAGASAPSS AFMGNSLKKV SARFNNNGKA
51 PVGSFMIVAA KEIDEDTQTD QDRWK**GLAYD ISDDQQDITR** GKGMVDSLFQ
101 APMNDGTHYA VMSSYEYISQ GLRTYDLDNN MDGFYIAPAF MDKLVVHITK
151 NYMTLPNIKV PLILGIWGGK GQGKSFQCEL VFAKMGINPI MMSAGELESG
201 NAGEPAKLIR QRYREAADII KKGKMCCLFI NDLDAGAGRM GGTTQYTVNN
251 QMVNATLMNI ADNPTNVQLP GMYNKEENPR **VPIIVTGNDF STLYAPLIR**D
301 GRMEK**FYWAP TR**EDR**IGVCT GIFR**TDNVPV DDIVK**LVDTF PGQSIDFFGA**
351 **LR**ARVYDDEV RKWIGEVGVN SVGKKLVNSR EGPPSFEQPT MTIEKLLEYG
401 NMLVAEQENV KRVQLADK**YL SEAALGNAND DAIKR**GAF

Spot No.: B22

Protein name: Rubisco activase

Peptide sequences:

K.MCCLFINDLDAGAGR.M;R.VPIIVTGNDFSTLYAPLIR.D;K.FYWAPTR.E;R.IGVCTGIFR.T;K.LVDTFPGQSIDFFGALR.A

Accession No.: Gohir.A10G221700.1.p

Mascot score: 491 **Sequence coverage %:**15

Calculated Mr: 48189 **Calculated *p*I:** 5.40

PFF Searched Score:

Ions score is -10*Log(P), where P is the probability that the observed match is a random event.
Individual ions scores > 35 indicate identity or extensive homology (p<0.05).
Protein scores are derived from ions scores as a non-probabilistic basis for ranking protein hits.

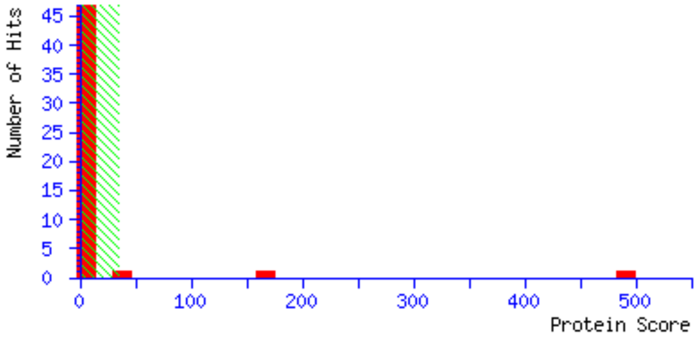

Matched peptide sequences: shown in Bold Red:

```
  1 MAAAVSTIGA VNRAPLSLNG SGAGASAPSS AFMGNSLKKV SARFNNNGKA
 51 PVGSFMIVAA KEIDEDTQTD QDRWKGLAYD ISDDQQDITR GKGMVDSLFQ
101 APMNDGTHYA VMSSYEYISQ GLRTYDLDNN MDGFYIAPAF MDKLVVHITK
151 NYMTLPNIKV PLILGIWGGK GQGKSFQCEL VFAKMGINPI MMSAGELESG
201 NAGEPAKLIR QRYREAADII KKGKMCCLFI NDLDAGAGRM GGTTQYTVNN
251 QMVNATLMNI ADNPTNVQLP GMYNKEENPR VPIIVTGNDF STLYAPLIRD
301 GRMEKFYWAP TREDRIGVCT GIFRTDNVPV DDIVKLVDTF PGQSIDFFGA
351 LRARVYDDEV RKWIGEVGVN SVGKKLVNSR EGPPSFEQPT MTIEKLLEYG
401 NMLVAEQENV KRVQLADKYL SEAALGNAND DAIKRGAF
```

Spot No.: C2

Protein name: Phosphoribulokinase

Peptide sequences:

K.ILVIEGLHPMFDER.V;R.VRDLLDFSIYLDISNEVK.F;K.HFSPVYLFDEGSTISWIPCGR.K;R.LDELIYVESHLSNISTR.F;K.IRDLYEQITSSK.T

Accession No.: Gohir.A12G204600.1.p

Mascot score: 532 **Sequence coverage %:**20

Calculated Mr: 45621 **Calculated *p*I:** 6.14

PFF Searched Score:

Ions score is -10*Log(P), where P is the probability that the observed match is a random event.
Individual ions scores > 34 indicate identity or extensive homology (p<0.05).
Protein scores are derived from ions scores as a non-probabilistic basis for ranking protein hits.

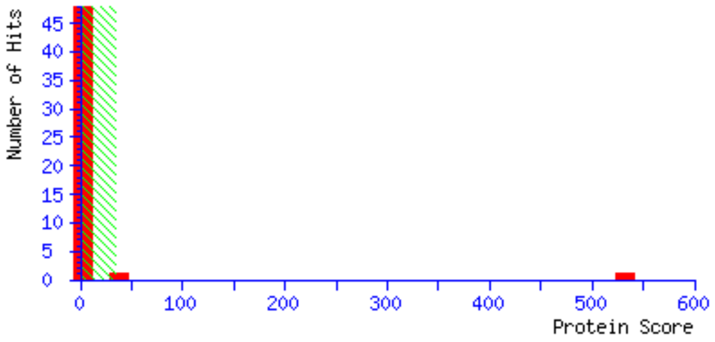

Matched peptide sequences: shown in Bold Red:

```
  1 MAVCSAYTTQ SLHSTCSIST PAKTHLGFHQ KQVVFYRRSS KRGGSGSSSG
 51 PCVITCSAGD SQTVVIGLAA DSGCGKSTFM RRLTSVFGGA AEPPKGGNPD
101 SNTLISDMTT VICLDDYHSL DRTGRKEKGV TALDPRANDF DLMYEQVKAI
151 KSGVAVDKPI YNHVTGLLDP PELIKPPKIL VIEGLHPMFD ERVRDLLDFS
201 IYLDISNEVK FAWKIQRDMA ERGHSLESIR ASIEARKPDF DAYIDPQKKY
251 ADAVIEVLPT QLIPDDNEGK VLRVRLIMKE GVKHFSPVYL FDEGSTISWI
301 PCGRKLTCSY PGIKFHYGPD TYFGNEVSIL EMDGQFDRLD ELIYVESHLS
351 NISTRFYGEV TQQMLKHADF PGSNNGTGLF QTIVGLKIRD LYEQITSSKT
401 AAPLEATKA
```

Spot No.: C3

Protein name: Phosphoribulokinase

Peptide sequences:

R.ANDFDLMYEQVK.A;K.ILVIEGLHPMFDER.V;R.VRDLLDFSIYLDISNEVK.F;R.DLLDFSIYLDISNEVK.F;K.EGVEHFSPVYLFDEGSTISWIPCGR.K;K.IRDLYEQITASK.T

Accession No.: Gohir.D03G177400.1.p

Mascot score: 331 **Sequence coverage %:** 19

Calculated Mr: 45591 **Calculated** ***p*****I:** 5.96

PFF Searched Score:

Ions score is -10*Log(P), where P is the probability that the observed match is a random event.
Individual ions scores > 34 indicate identity or extensive homology (p<0.05).
Protein scores are derived from ions scores as a non-probabilistic basis for ranking protein hits.

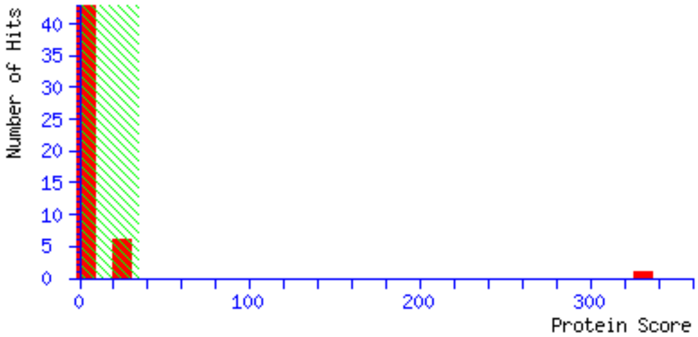

Matched peptide sequences: shown in Bold Red:

```
  1 MAVCPVYTTQ SLNSTCSIST PSKTHFSSHH NHLVFYRTSK RTSKRGGSSS
 51 CVITCSAGDS QTVVIGLAAD SGCGKSTFMR RLTSVFGGAA EPPKGGNPDS
101 NTLISDMTTV ICLDDYHSLD RTGRKEKGVT ALDPRANDFD LMYEQVKALK
151 SGIAVDKPIY NHVTGLLDPP ELIKPPKILV IEGLHPMFDE RVRDLLDFSI
201 YLDISNEVKF AWKIQRDMAE RGHSLESIKA SIEARKPDFD AFIDPQKQYA
251 DAVIEVLPTQ LIPDDNEGKV LRVRLIMKEG VEHFSPVYLF DEGSTISWIP
301 CGRKLTCSYP GIKFSYGPDT YFGHEVSVLE MDGQFDRLDE LIYVESHLSN
351 LSTKFYGEVT QQMLKHADFP GSNNGTGLFQ TIVGLKIRDL YEQITASKTA
401 APLQATKA
```

Spot No.: C4

Protein name: Phosphoribulokinase

Peptide sequences:

K.GGNPDSNTLISDMTTVICLDDYHSLDR.T;K.ILVIEGLHPMFDER.V;R.VRDLLDFSIYLDISNEVK.F;R.KPDFDAFIDPQK.Q;K.EGVEHFSPVYLFDEGSTISWIPCGR.K

Accession No.: Gohir.D03G177400.1.p

Mascot score: 357 **Sequence coverage %:**23

Calculated Mr: 45591 **Calculated** ***p*I:** 5.96

PFF Searched Score:

Ions score is -10*Log(P), where P is the probability that the observed match is a random event.
Individual ions scores > 34 indicate identity or extensive homology (p<0.05).
Protein scores are derived from ions scores as a non-probabilistic basis for ranking protein hits.

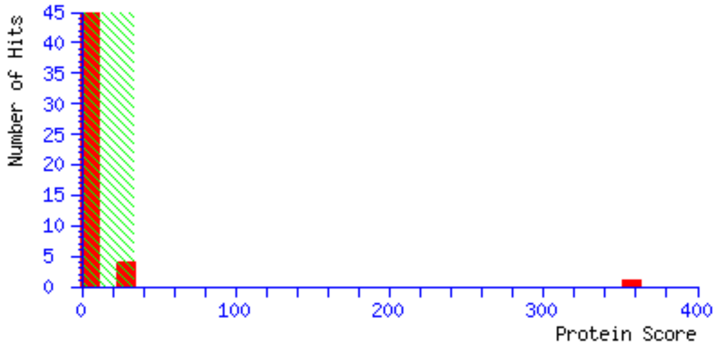

Matched peptide sequences: shown in Bold Red:

```
  1 MAVCPVYTTQ SLNSTCSIST PSKTHFSSHH NHLVFYRTSK RTSKRGGSSS
 51 CVITCSAGDS QTVVIGLAAD SGCGKSTFMR RLTSVFGGAA EPPKGGNPDS
101 NTLISDMTTV ICLDDYHSLD RTGRKEKGVT ALDPRANDFD LMYEQVKALK
151 SGIAVDKPIY NHVTGLLDPP ELIKPPKILV IEGLHPMFDE RVRDLLDFSI
201 YLDISNEVKF AWKIQRDMAE RGHSLESIKA SIEARKPDFD AFIDPQKQYA
251 DAVIEVLPTQ LIPDDNEGKV LRVRLIMKEG VEHFSPVYLF DEGSTISWIP
301 CGRKLTCSYP GIKFSYGPDT YFGHEVSVLE MDGQFDRLDE LIYVESHLSN
351 LSTKFYGEVT QQMLKHADFP GSNNGTGLFQ TIVGLKIRDL YEQITASKTA
401 APLQATKA
```

Spot No.: C6

Protein name: Malate dehydrogenase 1

Peptide sequences:

R.VLVTGAAGQIGYALVPMIAR.G;K.MELVDAAFPLLK.G;K.GVVATTDAVEACTGVNVAVMVGGFPR.K;R.ELVKDDAWLNGEFITTVQQR.G;R.KLSSALSAASAACDHIR.D

Accession No.: Gohir.D02G045500.1.p

Mascot score: 255 **Sequence coverage %:**28

Calculated Mr: 35830 **Calculated *p*I:** 6.10

PFF Searched Score:

Ions score is -10*Log(P), where P is the probability that the observed match is a random event.
Individual ions scores > 33 indicate identity or extensive homology (p<0.05).
Protein scores are derived from ions scores as a non-probabilistic basis for ranking protein hits.

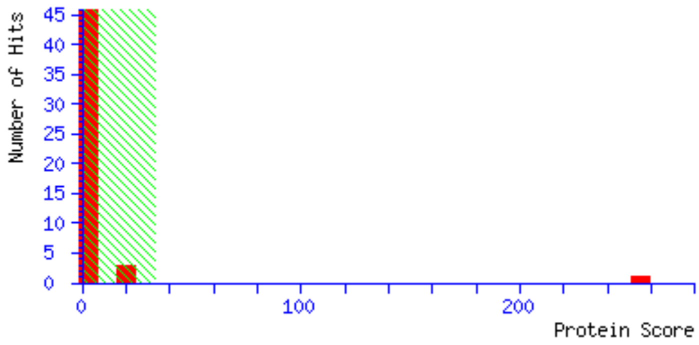

Matched peptide sequences: shown in Bold Red:

```
  1 MAKEPVRVLV TGAAGQIGYA LVPMIARGVM LGADQPVILH MLDIPPAAEA
 51 LNGVKMELVD AAFPLLKGVV ATTDAVEACT GVNVAVMVGG FPRKEGMERK
101 DVMSKNVSIY KSQASALEQH AAPNCKVLVV ANPANTNALI LKEFAPSIPA
151 KNITCLTRLD HNRALGQISE KLNVQVSDVK NVIIWGNHSS TQYPDVNHAT
201 VMTPSGEKPV RELVKDDAWL NGEFITTVQQ RGAAIIKARK LSSALSAASA
251 ACDHIRDWVL GTPEGTWVSM GVYSDGSYNA PAGVIYSFPV TCKNGEWTIV
301 QGLAIDEFSR KKLDLTGVEL TEEKELAYSC LS
```

Spot No.: C7

Protein name: Coproporphyrinogen-III oxidase 1

Peptide sequences:

K.ETLISERPHTFLR.E;K.NPFAPTLHFNYR.Y;K.FDPSFYPR.F;R.YVEFNLVYDR.G;R.IESILVSLPLSAR.W

Accession No.: Gohir.A08G050600.1.p

Mascot score: 281 **Sequence coverage %:**14

Calculated Mr: 44571 **Calculated *p*I:** 6.28

PFF Searched Score:

Ions score is -10*Log(P), where P is the probability that the observed match is a random event.
Individual ions scores > 35 indicate identity or extensive homology (p<0.05).
Protein scores are derived from ions scores as a non-probabilistic basis for ranking protein hits.

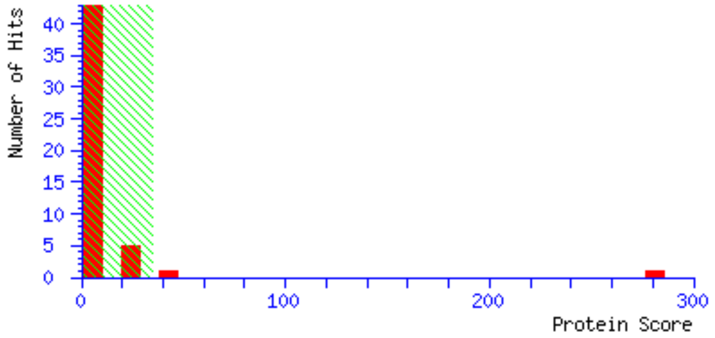

Matched peptide sequences: shown in Bold Red:

1 MPPPTAALSV PSSSSPSLFP LSSFSSSNNP HVSSFCPAPT ALPKFPSLSK
51 PKTPLRNLTP LSAVSIEK**ET** **LISERPHTFL** **R**EADGGDDGS VRSRFQRMIL
101 EAQESVCSAL EAVDGAGKFK EDAWTRPGGG GGISRVLQDG AVFEKAGVNI
151 SIVYGVMPPE AYRAAKAAAD DQKPGPIPFF AAGISSVLHP K**NPFAPTLHF**
201 **NYR**YFETDAP KDAPGAPRQW WFGGGTDLTP AYIFEEDVKH FHSIQKKACD
251 K**FDPSFYPR**F KKWCDDYFYI KHRGERRGLG GIFFDDLNDY DQEMLLSFAT
301 ECANSVVPAY IPIIEKRKDT PFNESQKAWQ QLRRGR**YVEF** **NLVYDR**GTTF
351 GLKTGGR**IES** **ILVSLPLSAR** WEYDHKPEEG SEEWKLLDAC INPKEWI

Spot No.: C8

Protein name: Ribulose-bisphosphate carboxylases

Peptide sequences:

K.DTDILAAFR.V;K.TFQGPPHGIQVER.D;R.AVYECLR.G;R.GG
LDFTKDDENVNSQPFMR.W

Accession No.: Gohir.A03G084800.1.p

Mascot score: 250 **Sequence coverage %:**9

Calculated Mr: 54651 **Calculated *p*I:** 6.00

PFF Searched Score:

Ions score is -10*Log(P), where P is the probability that the observed match is a random event.
Individual ions scores > 35 indicate identity or extensive homology (p<0.05).
Protein scores are derived from ions scores as a non-probabilistic basis for ranking protein hits.

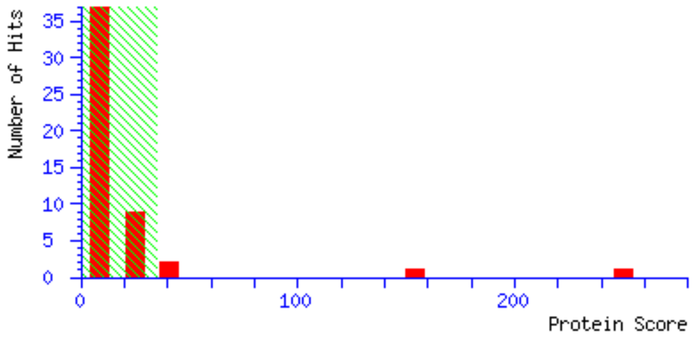

Matched peptide sequences: shown in Bold Red:

```
  1 MSCREGLMSP QTETKASVGF KAGVKEYKLT YYTPEYEVKD TDILAAFRVT
 51 PQPGVPPEEA GAAVAAESST GTWTTVWTDG LTSLDRYKGR CYDIEPVPGE
101 EDQYICYVAY PLDLFEEGSV TNMFTSIVGN VFGFKALRAL RLEDLRVPTA
151 YIKTFQGPPH GIQVERDKLN KYGRPLLGCT IKPKLGLSAK NYGRAVYECL
201 RGGLDFTKDD ENVNSQPFMR WRDRFLFCAE AIFKSQAETG EIKGHYLNAT
251 AGTCEEMIKR AMCARELGVP IVMHDYLTGG FTANTSLAHY CRDNGLLLHI
301 HRAMHAVIDR QKNHGMHFRV LAKALRMSGG DHIHAGTVVG KLEGERDITL
351 GFVDLLRDDF IEKDRSRGIY FTQDWVSMPG VLPVASGGIH VWHMPALTEI
401 FGDDSVLQFG GGTLGHPWGN APGAVANRVA LEACVQARNE GRDLAREGNE
451 IIREASKWSP ELAAACEVWK AIKFEFDAVD KLDKVEK
```

Spot No.: C11

Protein name: 30S ribosomal protein, putative

Peptide sequences: R.AEEDAETVYGSIDLVSSILQR.K

Accession No.: Gohir.1Z057400.1.p

Mascot score: 207 **Sequence coverage %:**7

Calculated Mr: 32072 **Calculated *p*I:** 5.53

PFF Searched Score:

Ions score is -10*Log(P), where P is the probability that the observed match is a random event.
Individual ions scores > 34 indicate identity or extensive homology (p<0.05).
Protein scores are derived from ions scores as a non-probabilistic basis for ranking protein hits.

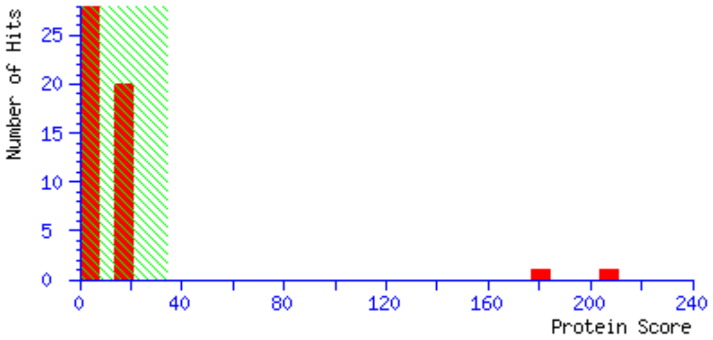

Matched peptide sequences: shown in Bold Red:

```
  1 MATTLLASSQ TSFHHPLSVS DSSFSSPSSS SSSVSMFTLT KPRIPSLPSY
 51 KSSFFNPIGN YFKCVEMEPR KIRNSSLTVR MSWDGPLASV KLIIQGKSLE
101 LTDTVKQHVE EKVTLFTKKH GVVRAEEDAE TVYGSIDLVS SILQRKLRKI
151 KEKESDRGRH MKGFSRSKVR EPVAVVVDDD AEAVPEQVVA DDDVAAVPEQ
201 EDDSFIDEIV RTKYFEMPPL TVSEAVEQLE NVDHDFYGFR NEETGEINII
251 YKRKAGGYGL IIPKGNGKAE KLEPLVVESA KEHSYVE
```

Spot No.: C12

Protein name:

Peptide sequences:

Accession No.:

Mascot score: **Sequence coverage %:**

Calculated Mr: **Calculated *p*I:**

PFF Searched Score:

Ions score is -10*Log(P), where P is the probability that the observed match is a random event.
Individual ions scores > 34 indicate identity or extensive homology (p<0.05).
Protein scores are derived from ions scores as a non-probabilistic basis for ranking protein hits.

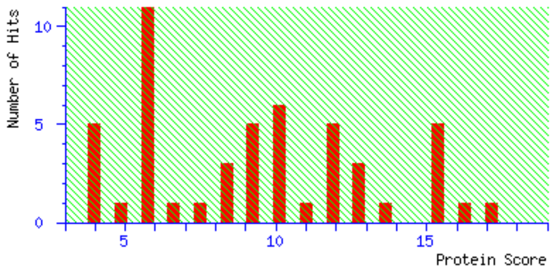

Matched peptide sequences: shown in Bold Red:

Spot No.: C14

Protein name: Photosystem II subunit O-2

Peptide sequences:

K.RLTYDEIQSK.T;K.GTGTANQCPTIDGGVDSFAFKPGK.Y;K.FEEKDGIDYAAVTVQLPGGER.V;K.DGIDYAAVTVQLPGGER.V;R.VPFLFTIK.Q;K.QLVASGKPDSFGGDFLVPSYR.G;R.GGSTGYDNAVALPAGGR.G

Accession No.: Gohir.D11G194600.1.p

Mascot score: 912 **Sequence coverage %:**30

Calculated Mr: 35293 **Calculated *p*I:** 5.37

PFF Searched Score:

Ions score is -10*Log(P), where P is the probability that the observed match is a random event.
Individual ions scores > 34 indicate identity or extensive homology (p<0.05).
Protein scores are derived from ions scores as a non-probabilistic basis for ranking protein hits.

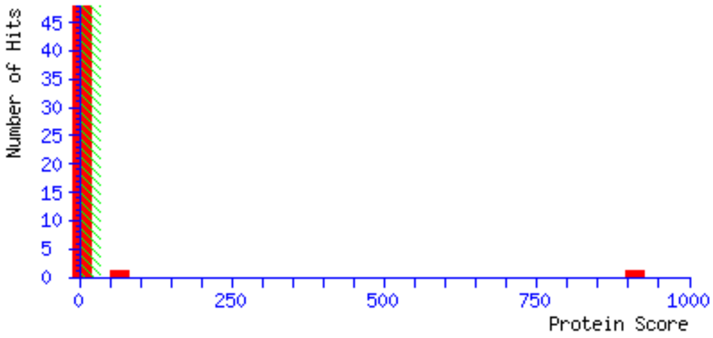

Matched peptide sequences: shown in Bold Red:

```
  1 MSVSLQAAAT LMQPIKVAAP SRTNVLLRSS QSVSKAFGLE PVGARLTCSL
 51 QTDLKDLAQT CVDATKLAGF ALATSALVVS GASAEGVPKR LTYDEIQSKT
101 YMEVKGTGTA NQCPTIDGGV DSFAFKPGKY YAKKFCLEPT SFTVKAEGVN
151 KNAPPEFQNT KLMTRLTYTL DEIEGPFEVS TDGTVKFEEK DGIDYAAVTV
201 QLPGGERVPF LFTIKQLVAS GKPDSFGGDF LVPSYRGSSF LDPKGRGGST
251 GYDNAVALPA GGRGDEEELA KENNKSAASS SGKITLSVTK SKPETGEVIG
301 VFESLQPSDT DLGAKTPKDV KITGVWYAQL DS
```

Spot No.: C15

Protein name: 2-phosphoglycolate phosphatase 1

Peptide sequences:

K.LIDGVPETLDMLR.S;K.VQYGTLCIR.E;R.ENPGCLFIATNR.D

Accession No.: Gohir.A02G108400.1.p

Mascot score: 79 **Sequence coverage %:** 9

Calculated Mr: 41067 **Calculated *p*I:** 8.45

PFF Searched Score:

Ions score is -10*Log(P), where P is the probability that the observed match is a random event.
Individual ions scores > 34 indicate identity or extensive homology (p<0.05).
Protein scores are derived from ions scores as a non-probabilistic basis for ranking protein hits.

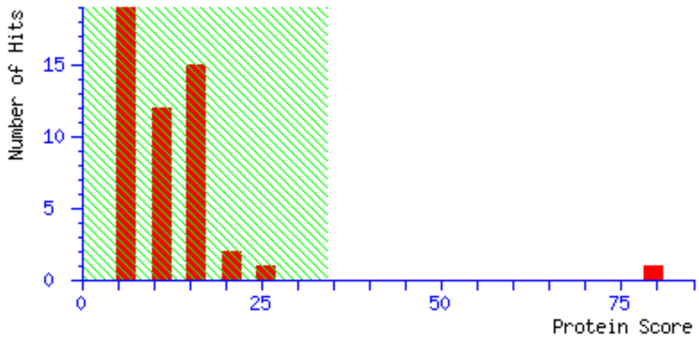

Matched peptide sequences: shown in Bold Red:

1 MATKAAVSVA ASVTAASSSS SKFVINTPHR FLCLKRLSSF SSFSASALAI
51 KCTSISNCSP NKSARSMEGF TTRTKASAQP LENADELIAS VETFIFDCDG
101 VIWKGDK**LID GVPETLDMLR** SKGKRLVFVT NNSTKSRKQY GKKFETLGLN
151 VNEEEIFASS FAAAYLKSI NFPKDKKVYV IGEDGILKEL ELAGFQYLGG
201 PEDGGKKIEL KPGFLMEHDK DVGAVVVGFD RYFNYYK**VQY GTLCIRENPG**
251 **CLFIATNR**DA VTHLTDAQEW AGGGSMVGAI CGSTQREPLV VGKPSTFMMD
301 YLANKFGILK SQICMVGDRL DTDILFGQNG GCKTLLVLSG VTNLSMLQSP
351 SNSIQPDFYT NKISDFLSIK TATV

Spot No.: C16

Protein name: Oxygen-evolving enhancer protein 1

Peptide sequences:

K.FEEKDGIDYAAVTVQLPGGER.V;K.DGIDYAAVTVQLPGGE R.V;R.VPFLFTIK.Q;K.QLVASGKPDSFGGDFLVPSYR.G;R.GGS TGYDNAVALPAGGR.G

Accession No.: Gohir.A11G188100.1.p

Mascot score: 343 **Sequence coverage %:** 20

Calculated Mr: 35233 **Calculated *p*I:** 5.84

PFF Searched Score:

Ions score is -10*Log(P), where P is the probability that the observed match is a random event.
Individual ions scores > 34 indicate identity or extensive homology (p<0.05).
Protein scores are derived from ions scores as a non-probabilistic basis for ranking protein hits.

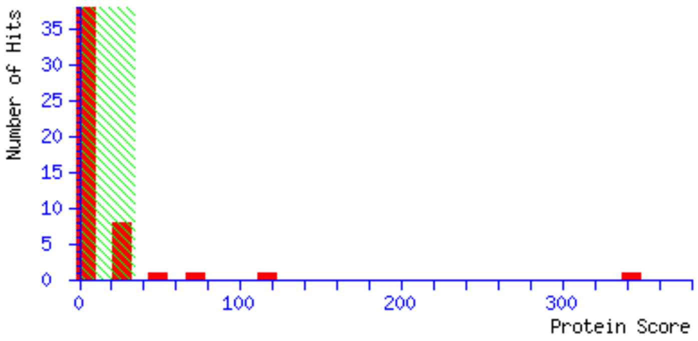

Matched peptide sequences: shown in Bold Red:

```
  1 MAVSLQAAAT LMQPIKVAAP SRTNVPLRSS QSVSKAFGLE PVGARLTCSL
 51 QTDLKDLALK CVDATKLAGF ALATSALVVS GASAEGVSKR LTYAEIQSKT
101 YMEVKGTGTA NQCPTIDGGV DSFAFKPGKY YAKKFCLEPT SFTVKAEGVN
151 KNAPPEFQNT KLMTRLTYTL DEIEGPFEVS TDGTLKFEEK DGIDYAAVTV
201 QLPGGERVPF LFTIKQLVAS GKPDSFGGDF LVPSYRGSSF LDPKGRGGST
251 GYDNAVALPA GGRGDEEELA KENNKSAASS SGKITLSVTK SKPETGEVIG
301 VFESLQPSDT DLGAKTPKDV KITGVWYAQL DS
```

Spot No.: C17

Protein name: Oxygen-evolving enhancer protein 1

Peptide sequences:

K.GTGTANQCPTIDGGVDSFAFKPGK.Y;K.FEEKDGIDYAAVTVQLPGGER.V;K.DGIDYAAVTVQLPGGER.V;R.VPFLFTIK.Q;K.QLVASGKPDSFGGDFLVPSYR.G;R.GGSTGYDNAVALPAGGR.G

Accession No.: Gohir.A11G188100.1.p

Mascot score: 853 **Sequence coverage %:** 27

Calculated Mr: 35233 **Calculated *p*I:** 5.84

PFF Searched Score:

Ions score is -10*Log(P), where P is the probability that the observed match is a random event.
Individual ions scores > 33 indicate identity or extensive homology (p<0.05).
Protein scores are derived from ions scores as a non-probabilistic basis for ranking protein hits.

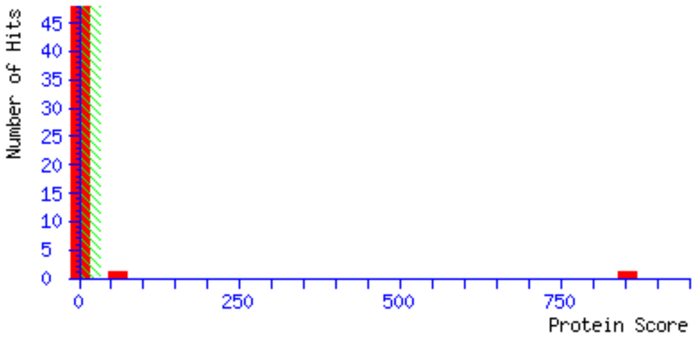

Matched peptide sequences: shown in Bold Red:

1 MAVSLQAAAT LMQPIKVAAP SRTNVPLRSS QSVSKAFGLE PVGARLTCSL
51 QTDLKDLALK CVDATKLAGF ALATSALVVS GASAEGVSKR LTYAEIQSKT
101 YMEVK**GTGTA** **NQCPTIDGGV** **DSFAFKPGK**Y YAKKFCLEPT SFTVKAEGVN
151 KNAPPEFQNT KLMTRLTYTL DEIEGPFEVS TDGTLK**FEEK** **DGIDYAAVTV**
201 **QLPGGERVPF** **LFTIKQLVAS** **GKPDSFGGDF** **LVPSYR**GSSF LDPKGR**GGST**
251 **GYDNAVALPA** **GGR**GDEEELA KENNKSAASS SGKITLSVTK SKPETGEVIG
301 VFESLQPSDT DLGAKTPKDV KITGVWYAQL DS

Spot No.: C18

Protein name:

Peptide sequences:

Accession No.:

Mascot score: **Sequence coverage %:**

Calculated Mr: **Calculated *p*I:**

PFF Searched Score:

Ions score is -10*Log(P), where P is the probability that the observed match is a random event.
Individual ions scores > 34 indicate identity or extensive homology (p<0.05).
Protein scores are derived from ions scores as a non-probabilistic basis for ranking protein hits.

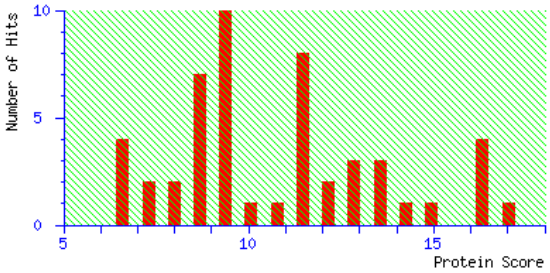

Matched peptide sequences: shown in Bold Red:

Spot No.: C20

Protein name: Ribose 5-phosphate isomerase, type A protein

Peptide sequences:

K.SGMILGLGTGSTAAFVVDK.I;K.RTQEQAASLNIPLSTLDLHPR.I;R.TQEQAASLNIPLSTLDLHPR.I;R.IDLAIDGADEVDPNLDLVK.G

Accession No.: Gohir.A11G253800.1.p

Mascot score: 288 **Sequence coverage %:** 20

Calculated Mr: 29906 **Calculated *p*I:** 6.44

PFF Searched Score:

Ions score is -10*Log(P), where P is the probability that the observed match is a random event.
Individual ions scores > 33 indicate identity or extensive homology (p<0.05).
Protein scores are derived from ions scores as a non-probabilistic basis for ranking protein hits.

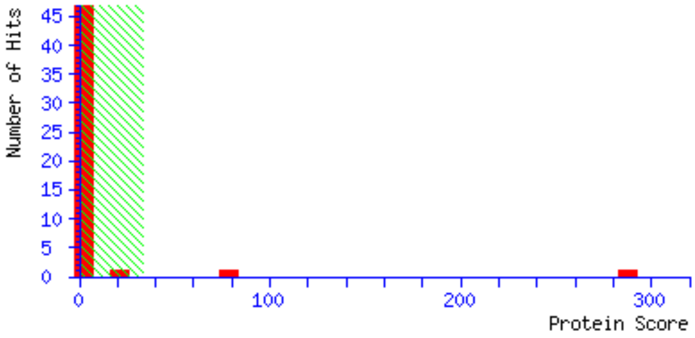

Matched peptide sequences: shown in Bold Red:

1 MASLSLNLSS LHHTSINPLI LRCTPLNLRT PSQKPFSIRS QAAPVLSQDD
51 LKKLAADKAV ESVK**SGMILG** **LGTGSTAAFV** **VDK**IGQLLST GQLSNIVGIP
101 TSK**RTQEQAA** **SLNIPLSTLD** **LHPRIDLAID** **GADEVDPNLD** **LVK**GRGGALL
151 REKIVEAASS SFIVVADESK LVSGLGGSGL AMPVEVVQFC WKYNLIRLQG
201 LFKELGCEAK LRLVGDGSEK PYVTDNGNYI VDLYFKNPIK DGFGAGKEIS
251 ALEGVVEHGL FLGMATSVII AGKTGIEVMT K

Spot No.: C24

Protein name: Photosystem I light harvesting complex gene 3

Peptide sequences:

K.AGLIPAETALPWFR.T;K.YLGGSGEPAYPGGPLFNPLGFGKDEK.S

Accession No.: Gohir.A11G146500.1.p

Mascot score: 198 **Sequence coverage %:**14

Calculated Mr: 29458 **Calculated *p*I:** 8.86

PFF Searched Score:

Protein score is -10*Log(P), where P is the probability that the observed match is a random event.
Protein scores greater than 62 are significant (p<0.05).
Protein scores are derived from ions scores as a non-probabilistic basis for ranking protein hits.

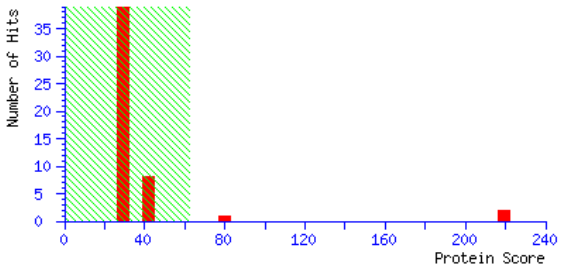

Matched peptide sequences: shown in Bold Red:

```
  1 MATQALVSSS LTYSVETARQ ILGAKRQIGS SRKGSFVVKA ASTPPVKQGA
 51 DRPLWFASKQ SLSYLDGSLP GDYGFDPLGL SDPEGPGGFI EPKWLAYGEI
101 INGRYAMLGA VGAIAPEILG KAGLIPAETA LPWFRTGVIP PAGTYNYWAD
151 PYTLFVFEMA LMGFAEHRRF QDWAKPGSMG KQYELGFEKY LGGSGEPAYP
201 GGPLFNPLGF GKDEKSLKDL KLKEVKNGRL AMLAILGYFI QGLVTGVGPY
251 QNLLDHLADP FNNNVLTNLK FH
```

Spot No.: D1

Protein name:

Peptide sequences:

Accession No.:

Mascot score: **Sequence coverage %:**

Calculated Mr: **Calculated *p*I:**

PFF Searched Score:

Ions score is -10*Log(P), where P is the probability that the observed match is a random event.
Individual ions scores > 34 indicate identity or extensive homology ($p<0.05$).
Protein scores are derived from ions scores as a non-probabilistic basis for ranking protein hits.

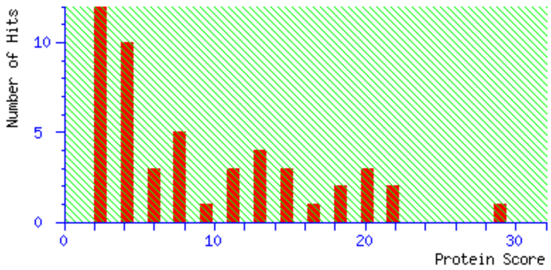

Matched peptide sequences: shown in Bold Red:

Spot No.: D3

Protein name: Oxygen-evolving enhancer protein 2-1

Peptide sequences:

K.TDTEFMPYNGDGFK.L;K.EVEFPGQVLR.Y;K.SITDYGSPEEFLSSVDYLLGK.Q;K.TDSEGGFDSGAVATANILESSSSTVGGKPYYFLSVLTR.T;K.PYYFLSVLTR.T

Accession No.: Gohir.D04G034900.1.p

Mascot score: 507 **Sequence coverage %:**31

Calculated Mr: 28189 **Calculated *p*I:** 7.67

PFF Searched Score:

Ions score is -10*Log(P), where P is the probability that the observed match is a random event.
Individual ions scores > 34 indicate identity or extensive homology (p<0.05).
Protein scores are derived from ions scores as a non-probabilistic basis for ranking protein hits.

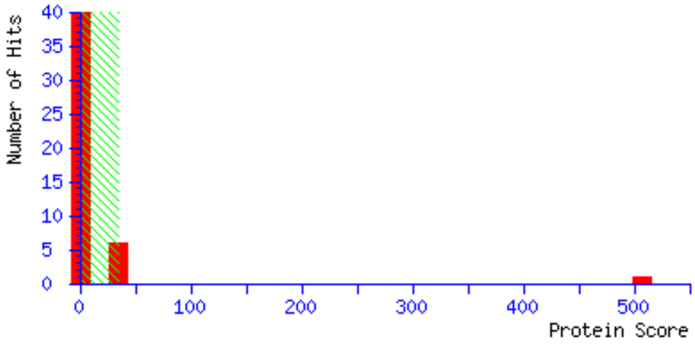

Matched peptide sequences: shown in Bold Red:

```
  1 MASAACFLHH HALTTNGRSS SLPPSLRQVS NMKPNQQVVC KAQKQEDDGS
 51 LVSRRLALTV LIGAAAVGSK VSPADAAYGE AANVFGKPKT DTEFMPYNGD
101 GFKLSIPSKW NPSKEVEFPG QVLRYEDNFD TTSNLSVMIT PTDKKSITDY
151 GSPEEFLSSV DYLLGKQAYF GKTDSEGGFD SGAVATANIL ESSSSTVGGK
201 PYYFLSVLTR TADGDEGGKH QLITATVNNG KLYICKAQAG DKRWFKGARK
251 FVESAASSFS VA
```

Spot No.: D4

Protein name: Photosystem II subunit P-1

Peptide sequences:

K.TDTDFMPYSGDGFK.L;K.EVEFPGQVLR.Y;K.SITDYGSPEEFLSSVDYLLGK.Q;K.TDAEGGFDSGAVATANILESSSSTVGGKPYYFLSVLTR.T;K.PYYFLSVLTR.T

Accession No.: Gohir.A05G380700.1.p

Mascot score: 444 **Sequence coverage %:**31

Calculated Mr: 28109 **Calculated *p*I:** 7.67

PFF Searched Score:

Ions score is -10*Log(P), where P is the probability that the observed match is a random event.
Individual ions scores > 34 indicate identity or extensive homology (p<0.05).
Protein scores are derived from ions scores as a non-probabilistic basis for ranking protein hits.

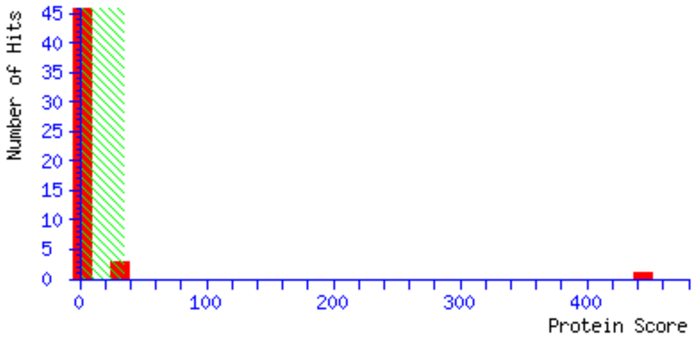

Matched peptide sequences: shown in Bold Red:

```
  1 MASAACFLHH HALTTNGRSS SLSPSLRQVS NMKPTQQVVC KAQKQEDDGS
 51 LVSRRLALTV LIGAAAVGSK VSPADAAYGE AANVFGKPKT DTDFMPYSGD
101 GFKLSIPSKW NPSKEVEFPG QVLRYEDNFD TTSNLSVMIT PTDKKSITDY
151 GSPEEFLSSV DYLLGKQAYF GKTDAEGGFD SGAVATANIL ESSSSTVGGK
201 PYYFLSVLTR TADGDEGGKH QLITATVNNG KLYICKAQAG DKRWFKGARK
251 FVESAASSFS VA
```

Spot No.: D5

Protein name: Ribulose-bisphosphate carboxylases

Peptide sequences:

K.LTYYTPEYEVK.D;K.DTDILAAFR.V;R.VTPQPGVPPEEAGAA VAAESSTGTWTTVWTDGLTSLDR.Y;K.TFQGPPHGIQVER.D

Accession No.: Gohir.A03G084800.1.p

Mascot score: 206 **Sequence coverage %:**14

Calculated Mr: 54651 **Calculated *p*I:** 6.00

PFF Searched Score:

Ions score is -10*Log(P), where P is the probability that the observed match is a random event.
Individual ions scores > 34 indicate identity or extensive homology (p<0.05).
Protein scores are derived from ions scores as a non-probabilistic basis for ranking protein hits.

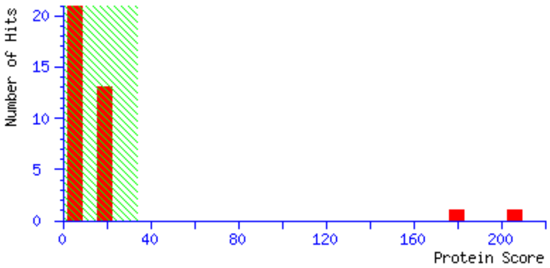

Matched peptide sequences: shown in Bold Red:

1 MSCREGLMSP QTETKASVGF KAGVKEYK**LT YYTPEYEVKD TDILAAFRVT**
51 **PQPGVPPEEA GAAVAAESST GTWTTVWTDG LTSLDR**YKGR CYDIEPVPGE
101 EDQYICYVAY PLDLFEEGSV TNMFTSIVGN VFGFKALRAL RLEDLRVPTA
151 YIK**TFQGPPH GIQVER**DKLN KYGRPLLGCT IKPKLGLSAK NYGRAVYECL
201 RGGLDFTKDD ENVNSQPFMR WRDRFLFCAE AIFKSQAETG EIKGHYLNAT
251 AGTCEEMIKR AMCARELGVP IVMHDYLTGG FTANTSLAHY CRDNGLLLHI
301 HRAMHAVIDR QKNHGMHFRV LAKALRMSGG DHIHAGTVVG KLEGERDITL
351 GFVDLLRDDF IEKDRSRGIY FTQDWVSMPG VLPVASGGIH VWHMPALTEI
401 FGDDSVLQFG GGTLGHPWGN APGAVANRVA LEACVQARNE GRDLAREGNE
451 IIREASKWSP ELAAACEVWK AIKFEFDAVD KLDKVEK

Spot No.: D10

Protein name: Photosynthetic electron transfer C

Peptide sequences:

K.FICPCHGSQYNDQGR.V;K.VVFVPWVETDFR.T

Accession No.: Gohir.D02G184300.1.p

Mascot score: 139 **Sequence coverage %:**11

Calculated Mr: 24700 **Calculated *p*I:** 8.48

PFF Searched Score:

Ions score is -10*Log(P), where P is the probability that the observed match is a random event.
Individual ions scores > 34 indicate identity or extensive homology (p<0.05).
Protein scores are derived from ions scores as a non-probabilistic basis for ranking protein hits.

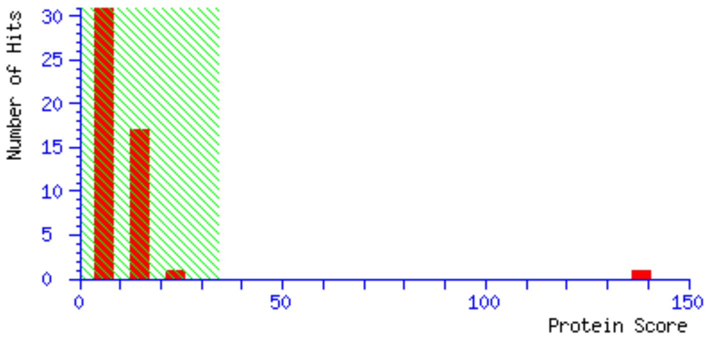

Matched peptide sequences: shown in Bold Red:

```
  1 MASSTLSPAA PWQLCSGKSG MFCPSQAFLV KPTRTHMVKN EKGMRITCQA
 51 TSIPADRVPD MGKRQLMNLL LLGAISLPSG FMLVPYAAFF VPSGGRGTGG
101 GTVAKDAIGN DVIAEEWLKT HGPGDRTLTQ GLKGDPTYLV VEKDRTLATY
151 GINAVCTHLG CVVPWNQAEN KFICPCHGSQ YNDQGRVVRG PAPLSLALAH
201 AGVEDGKVVF VPWVETDFRT GDGPWWS
```

Spot No.: D16

Protein name: Nucleoside diphosphate kinase 2

Peptide sequences:

R.GLVGEIISR.F;R.KLIGSTNPLQAEPGTIR.G;K.LIGSTNPLQAE PGTIR.G

Accession No.: Gohir.A09G245300.1

Mascot score: 120 **Sequence coverage %:**11

Calculated Mr: 26192 **Calculated *p*I:** 9.02

PFF Searched Score:

Ions score is -10*Log(P), where P is the probability that the observed match is a random event.
Individual ions scores > 34 indicate identity or extensive homology (p<0.05).
Protein scores are derived from ions scores as a non-probabilistic basis for ranking protein hits.

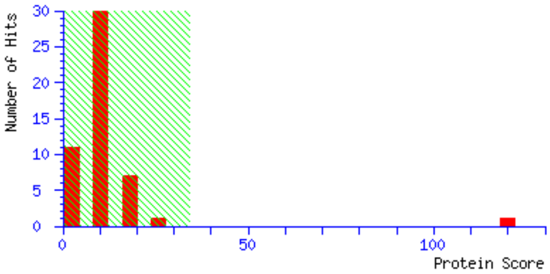

Matched peptide sequences: shown in Bold Red:

```
  1 METITLSPSL TTSSFGTSAA ATVKRSSATC LSYTSHANLN VNHLAAFHKQ
 51 SHLFTKSPTR PFVFTKTRAN KSAHGIFLPH LVASLEQVEQ TYIMVKPDGV
101 QRGLVGEIIS RFERKGFKLT GLKLFQCPKE LAEEHYKDLK TKSFYPTLID
151 YITSGPVVCM VWEGVGVVAS ARKLIGSTNP LQAEPGTIRG DLAVQTGRNV
201 VHGSDSPENG KRETALWFKE GELCEWTPAQ APWLME
```

Spot No.: D21

Protein name: Nucleoside diphosphate kinase family protein

Peptide sequences:

R.GLVGEIIGR.F;R.KIIGATNPAESAPGTIR.G;K.IIGATNPAESAPGTIR.G;R.NVIHGSDSVESAR.K

Accession No.: Gohir.D08G160400.1.p

Mascot score: 134 **Sequence coverage %:**26

Calculated Mr: 16414 **Calculated *p*I:** 6.30

PFF Searched Score:

Ions score is -10*Log(P), where P is the probability that the observed match is a random event.
Individual ions scores > 35 indicate identity or extensive homology (p<0.05).
Protein scores are derived from ions scores as a non-probabilistic basis for ranking protein hits.

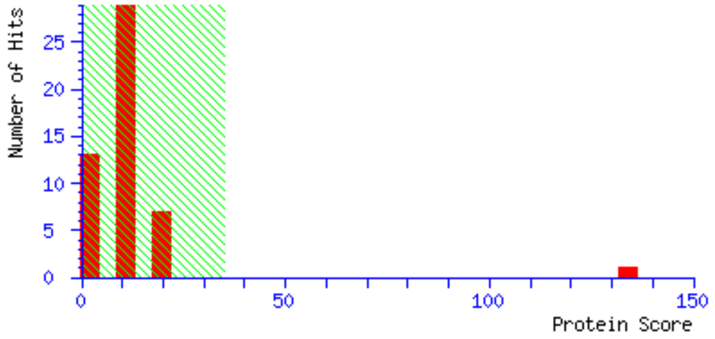

Matched peptide sequences: shown in Bold Red:

```
  1 MEQTFIMIKP DGVQRGLVGE IIGRFEKKGF YLKGLKLITV DQSFAEKHYA
 51 DLSAKPFFNG LVEYIISGPV VAMIWEGKNV VTTGRKIIGA TNPAESAPGT
101 IRGDFAIDIG RNVIHGSDSV ESARKEIALW FPESPVNWQS SVHPWIYE
```

Spot No.: E1

Protein name: Translation elongation factor EFG/EF2 protein

Peptide sequences:

R.INIIDTPGHVDFTLEVER.A;K.LAQEDPSFHFSR.D;R.AVLVDG SYHDVDSSVLAFQLAAR.G

Accession No.: Gohir.A11G019900.1.p

Mascot score: 101 **Sequence coverage %:**6

Calculated Mr: 86600 **Calculated *p*I:** 5.54

PFF Searched Score:

Ions score is -10*Log(P), where P is the probability that the observed match is a random event.
Individual ions scores > 34 indicate identity or extensive homology (p<0.05).
Protein scores are derived from ions scores as a non-probabilistic basis for ranking protein hits.

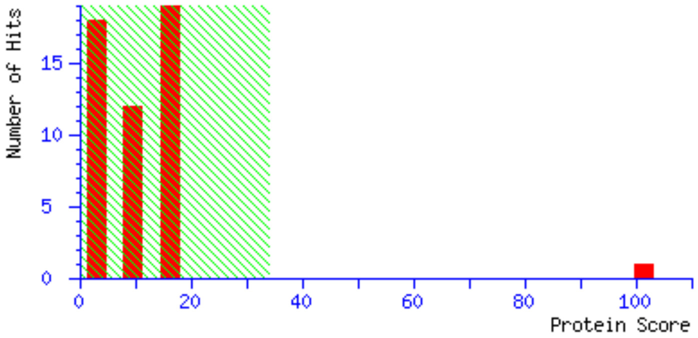

Matched peptide sequences: shown in Bold Red:

```
  1 MAAETALRVS SSSSTVCNLN GFQRRPTPLS SSTRFLGLRP RASSSSISSS
 51 LSQFMGSVRI GSRLPISLQQ KGKRRNFSLF AMAADESKRA VPLKDYRNIG
101 IMAHIDAGKT TTTERILYYT GRNYKIGEVH EGTATMDWME QEQERGITIT
151 SAATTTFWKD HRINIIDTPG HVDFTLEVER ALRVLDGAIC LFDSVAGVEP
201 QSETVWRQAD KYGVPRICFV NKMDRLGANF FRTRDMIVTN LGAKPLVIQL
251 PVGAEDNFKG VIDLVKMKAV LWSGEELGAK FEYADIPADL QELAEEYRSQ
301 MIETIVELDD QAMENYLEGV EPDEETIKKL IRKGTIGISF VPVLCGSAFK
351 NKGVQPLLDA VMDYLPSPLE LPAMKGTDPE NPEVIIERTA SDEEPFSGLA
401 FKIMTDPFVG SLTFVRVYSG KLAAGSYVLN ANKGKKERIG RLLEMHANSR
451 EDVKVALAGD IVALAGLKDT ITGETLSDPD HPIVLERMDF PDPVIKVAIE
501 PKTKADVDKM ANGLIKLAQE DPSFHFSRDE EINQTVIEGM GELHLEIIVD
551 RLKREFKVEA NVGAPQVNYR ESISKVSEVK YVHKKQSGGQ GQFADITVRF
601 EPMDAGSGYE FKSEIKGGAV PKEYIPGVMK GLEECMCNGV LAGFPVVDVR
651 AVLVDGSYHD VDSSVLAFQL AARGAFREGI RKAGPRMLEP IMKVEVVTPE
701 EHLGDVIGDL NSRRGQINSF GDKPGGLKVV DALVPLAEMF QYVSTLRGMT
751 KGRASYTMQL AKFDVVPQHI QNELATKQQE VVA
```

Spot No.: E3

Protein name: Chloroplast heat shock protein 70-2

Peptide sequences:

K.AVVTVPAYFNDSQR.T;R.VKFEELCSDLLDR.L;K.DIDEVILVGGSTR.I;R.IPAVQELVR.K;K.SEVFSTAADGQTSVEINVLQGER.E

Accession No.: Gohir.D09G212600.1.p

Mascot score: 464 **Sequence coverage %:**10

Calculated Mr: 75693 **Calculated *p*I:** 5.17

PFF Searched Score:

Ions score is -10*Log(P), where P is the probability that the observed match is a random event.
Individual ions scores > 34 indicate identity or extensive homology (p<0.05).
Protein scores are derived from ions scores as a non-probabilistic basis for ranking protein hits.

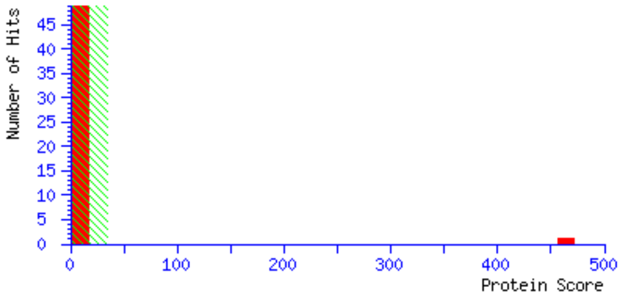

Matched peptide sequences: shown in Bold Red:

1 MASSTAQIHV LGGMGFDSSR KPNQHSSRTV FFGQRLGKTS PLNATFLRLA
51 KTKNSNGKGY NVGPVRVVNE KVVGIDLGTT NSAVAAMEGG KPTIVTNAEG
101 QRTTPSVVAY TKSGDRLVGQ IAKRQAVVNP ENTFFSVKRF IGRKMSEVDE
151 EAKQVSYKVI RDENGNVKLE CPAISKQFAA EEISAQVLRK LVDDASKFLS
201 ESVTK**AVVTV PAYFNDSQR**T ATKDAGRIAG LEVLRIINEP TAASLAYGFE
251 KKNNETILVF DLGGGTFDVS VLEVGDGVFE VLSTSGDTHL GGDDFDKRIV
301 DWLAENFKRD EGIDLLKDKQ ALQRLTEAAE KAKMELSTLT QTNISLPFIT
351 ATADGPKHIE TTLTR**VKFEE LCSDLLDR**LK KPVENALGDA KLSFK**DIDEV**
401 **ILVGGSTRIP AVQELVR**KMT GKEPNVTVNP DEVVALGAAV QAGVLAGDVS
451 DIVLLDVTPL SLGLETLGGV MTKIIPRNTT LPTSK**SEVFS TAADGQTSVE**
501 **INVLQGER**EF VRDNKSLGSF RLDGIPPAPR GVPQIEVKFD IDANGILSVT
551 AVDKGTGKKQ DITITGASTL PGDEVDRMVK EAERFAQEDK EKRDAIDTKN
601 QADSAVYQTE KQLKELGDKV PGPVKEKVDA KLQELKDAIS GGSTQGMKDA
651 MAALNQEVMQ LGQSLYNQPS AGSAAGPAPG GETGRSDSSN KGSDEDVIDA
701 DFTDSK

Spot No.: E5

Protein name: ATP-dependent zinc metalloprotease FTSH 8

Peptide sequences:

R.FLEYLDKDR.V;K.ENAPCIVFVDEIDAVGR.Q;R.ADILDSALLRPGR.F;K.KFETDVSFDVIAMR.T;R.TPGFSGADLANLLNEAAILAGR.R;R.ISDSAYEIALQHIR.N

Accession No.: Gohir.A01G022900.1.p

Mascot score: 377 **Sequence coverage %:**12

Calculated Mr: 74627 **Calculated *p*I:** 6.24

PFF Searched Score:

Ions score is -10*Log(P), where P is the probability that the observed match is a random event.
Individual ions scores > 34 indicate identity or extensive homology (p<0.05).
Protein scores are derived from ions scores as a non-probabilistic basis for ranking protein hits.

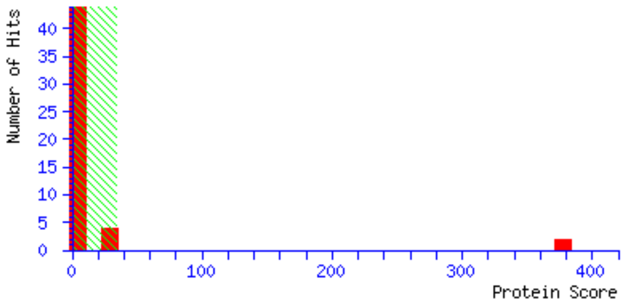

Matched peptide sequences: shown in Bold Red:

1 MAASSACLLG NGPSTHRTKS KLSKEFYGRN IVFTPAISSL GRKSNAVLVK
51 ASLKHKQHEG RRGFLKSLLG TAGIGVPALL GNGKAYADEQ GVSSSRMSYS
101 R**FLEYLDKDR** VKKVDLFENG TIAIVEAVSP ELGNRVQRVR VQLPGLSQEL
151 LQKFREKNID FAAHNAQEDS GSLLFNLIGN LAFPLILIGG LFLLSRRSSG
201 GMGGPGGPGF PLAFGQSKAK FQMEPNTGVT FDDVAGVDEA KQDFMEVVEF
251 LKKPERFTAV GARIPKGVLL IGPPGTGKTL LAKAIAGEAG VPFFSISGSE
301 FVEMFVGVGA SRVRDLFKKA K**ENAPCIVFV DEIDAVGR**QR GTGIGGGNDE
351 REQTLNQLLT EMDGFEGNTG IIVIAATNR**A DILDSALLRP GR**FDRQVTVD
401 VPDIRGRTEI LKVHGSNK**KF ETDVSFDVIA MRTPGFSGAD LANLLNEAAI**
451 **LAGR**RGKTAI SSKEIDDSID RIVAGMEGTV MTDGKSKSLV AYHEVGHAIC
501 GTLTPGHDPV QKVTLIPRGQ ARGLTWFIPS DDPTLISKQQ LFARIVGGLG
551 GRAAEEIIFG EPEVTTGAAG DLQQITGLAK QMVVTFGMSE IGPWSLMDSS
601 AQSADVIMRM MARNSMSEKL AEDIDTAVKR **ISDSAYEIAL QHIR**NNREAI
651 DKIVEVLLEK ETMSGDEFRA ILSEFVEIPA ENQVPPSVPT PVSV

Spot No.: E6

Protein name: Chaperonin-60alpha

Peptide sequences:

K.EIAFDQSSR.A;K.LADAVGLTLGPR.G;R.AIELPNAMENAGAALIR.E;K.LICEFENAR.V;K.DSTQIIADAASKDEIQAR.V

Accession No.: Gohir.A13G232000.1.p

Mascot score: 311   **Sequence coverage %:** 11

Calculated Mr: 62143   **Calculated *p*I:** 5.15

PFF Searched Score:

Ions score is -10*Log(P), where P is the probability that the observed match is a random event.
Individual ions scores > 34 indicate identity or extensive homology (p<0.05).
Protein scores are derived from ions scores as a non-probabilistic basis for ranking protein hits.

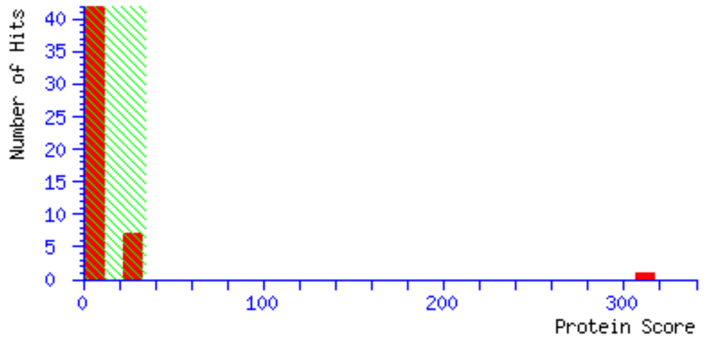

Matched peptide sequences: shown in Bold Red:

1 MASANALSSA SILCSPNKGS LRRKGNQRQN QRVNYRQGNN RFGVKACAK**E**
51 **IAFDQSSR**AA MQAGIDK**LAD AVGLTLGPR**G RNVVLDEFGS PKVVNDGVTI
101 AR**AIELPNAM ENAGAALIR**E VASKTNDSAG DGTTTASVLA REIIKLGLLT
151 VTSGANPVSV KRGIDKTVQS LIEELEKKAR PVKGRDDIKA VASISAGNDD
201 LIGTMVADAI DKVGPDGVLS IESSSSFETT VDVEEGMEID RGYISPQFVT
251 NPEK**LICEFE NAR**VLVTDQK ITAIKDIIPL LEKTTQLRAP LLIIAEDVTG
301 EALATLVVNK LRGILNVAAI KAPSFGERRK AVLQDIAILT GAEFQANDLG
351 LLIENTSVEQ LGIARKVIIT K**DSTQIIADA ASKDEIQAR**V QQLKKELAET
401 DSVYDTEKLA ERIAKLSGGV AVIKVGAATE TELEDRKLRI EDAKNATFAA
451 IEEGIVPGGG AALVHLSTCV PAIKDKLEDP EERIGADIVQ KALVAPASLI
501 AQNAGMEGEV VVEKVKNSEW EIGYNAMTDT YENLLAAGVI DPAKVTRCAL
551 QNAASVAGMV LTTQAIVVEK AKPKAPAAAA PEGLTI

Spot No.: E7

Protein name: Chaperonin-60alpha

Peptide sequences:

K.EIAFDQSSR.A;K.LADAVGLTLGPR.G;K.LICEFENAR.V;K.AVLQDIAILTGAEFQANDLGLLIENTSVEQLGIAR.K;K.DSTQIIADAASKDEIQAR.V

Accession No.: Gohir.A13G232000.1.p

Mascot score: 329 **Sequence coverage %:**14

Calculated Mr: 62143 **Calculated *p*I:** 5.15

PFF Searched Score:

Ions score is -10*Log(P), where P is the probability that the observed match is a random event.
Individual ions scores > 34 indicate identity or extensive homology (p<0.05).
Protein scores are derived from ions scores as a non-probabilistic basis for ranking protein hits.

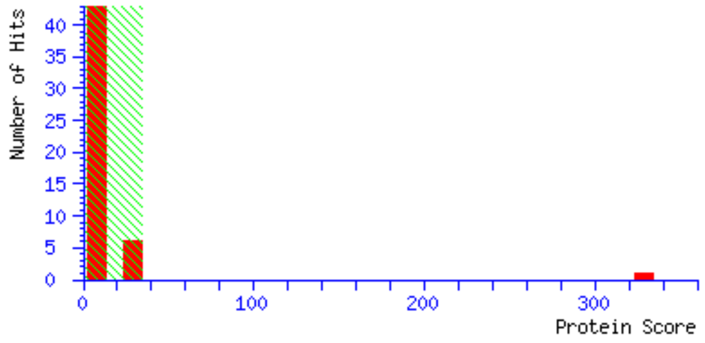

Matched peptide sequences: shown in Bold Red:

```
  1 MASANALSSA SILCSPNKGS LRRKGNQRQN QRVNYRQGNN RFGVKACAKE
 51 IAFDQSSRAA MQAGIDKLAD AVGLTLGPRG RNVVLDEFGS PKVVNDGVTI
101 ARAIELPNAM ENAGAALIRE VASKTNDSAG DGTTTASVLA REIIKLGLLT
151 VTSGANPVSV KRGIDKTVQS LIEELEKKAR PVKGRDDIKA VASISAGNDD
201 LIGTMVADAI DKVGPDGVLS IESSSSFETT VDVEEGMEID RGYISPQFVT
251 NPEKLICEFE NARVLVTDQK ITAIKDIIPL LEKTTQLRAP LLIIAEDVTG
301 EALATLVVNK LRGILNVAAI KAPSFGERRK AVLQDIAILT GAEFQANDLG
351 LLIENTSVEQ LGIARKVIIT KDSTQIIADA ASKDEIQARV QQLKKELAET
401 DSVYDTEKLA ERIAKLSGGV AVIKVGAATE TELEDRKLRI EDAKNATFAA
451 IEEGIVPGGG AALVHLSTCV PAIKDKLEDP EERIGADIVQ KALVAPASLI
501 AQNAGMEGEV VVEKVKNSEW EIGYNAMTDT YENLLAAGVI DPAKVTRCAL
551 QNAASVAGMV LTTQAIVVEK AKPKAPAAAA PEGLTI
```

Spot No.: E9

Protein name: ATP-dependent zinc metalloprotease FTSH 8

Peptide sequences:

R.FLEYLDKDR.V;K.AKENAPCIVFVDEIDAVGR.Q;K.ENAPCIVFVDEIDAVGR.Q;R.ADILDSALLRPGR.F;K.KFETDVSFDVIAMR.T;R.TPGFSGADLANLLNEAAILAGR.R;R.ISDSAYEIALQHIR.N

Accession No.: Gohir.A01G022900.1.p

Mascot score: 600 **Sequence coverage %:**13

Calculated Mr: 74627 **Calculated *p*I:** 6.24

PFF Searched Score:

Ions score is -10*Log(P), where P is the probability that the observed match is a random event.
Individual ions scores > 34 indicate identity or extensive homology (p<0.05).
Protein scores are derived from ions scores as a non-probabilistic basis for ranking protein hits.

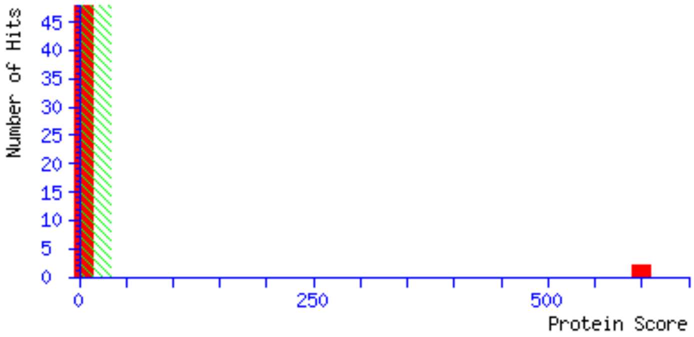

Matched peptide sequences: shown in Bold Red:

```
  1 MAASSACLLG NGPSTHRTKS KLSKEFYGRN IVFTPAISSL GRKSNAVLVK
 51 ASLKHKQHEG RRGFLKSLLG TAGIGVPALL GNGKAYADEQ GVSSSRMSYS
101 RFLEYLDKDR VKKVDLFENG TIAIVEAVSP ELGNRVQRVR VQLPGLSQEL
151 LQKFREKNID FAAHNAQEDS GSLLFNLIGN LAFPLILIGG LFLLSRRSSG
201 GMGGPGGPGF PLAFGQSKAK FQMEPNTGVT FDDVAGVDEA KQDFMEVVEF
251 LKKPERFTAV GARIPKGVLL IGPPGTGKTL LAKAIAGEAG VPFFSISGSE
301 FVEMFVGVGA SRVRDLFKKA KENAPCIVFV DEIDAVGRQR GTGIGGGNDE
351 REQTLNQLLT EMDGFEGNTG IIVIAATNRA DILDSALLRP GRFDRQVTVD
401 VPDIRGRTEI LKVHGSNKKF ETDVSFDVIA MRTPGFSGAD LANLLNEAAI
451 LAGRRGKTAI SSKEIDDSID RIVAGMEGTV MTDGKSKSLV AYHEVGHAIC
501 GTLTPGHDPV QKVTLIPRGQ ARGLTWFIPS DDPTLISKQQ LFARIVGGLG
551 GRAAEEIIFG EPEVTTGAAG DLQQITGLAK QMVVTFGMSE IGPWSLMDSS
601 AQSADVIMRM MARNSMSEKL AEDIDTAVKR ISDSAYEIAL QHIRNNREAI
651 DKIVEVLLEK ETMSGDEFRA ILSEFVEIPA ENQVPPSVPT PVSV
```

Spot No.: E12

Protein name: Chaperonin Cpn60/TCP-1 family

Peptide sequences:

K.SAENSLYVVEGMQFDR.G;R.GYISPYFVTDSEK.M;R.DLINILEDAIR.S;R.KSQYLDDIAILTGGTVIR.D;K.SQYLDDIAILTGGTVIRDEVGLSLDK.A;K.AAVEEGIVVGGGCTLLR.L

Accession No.: Gohir.D10G200300.1.p

Mascot score: 497 **Sequence coverage %:**13

Calculated Mr: 64709 **Calculated *p*I:** 5.72

PFF Searched Score:

Ions score is -10*Log(P), where P is the probability that the observed match is a random event.
Individual ions scores > 34 indicate identity or extensive homology (p<0.05).
Protein scores are derived from ions scores as a non-probabilistic basis for ranking protein hits.

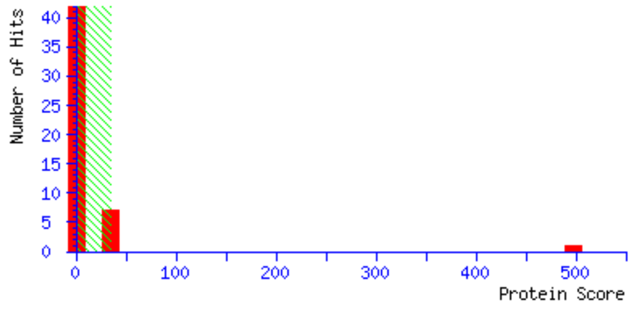

Matched peptide sequences: shown in Bold Red:

1 MASTFTAMSS VGSFVAPNGL VMDKKLSSSS NRLSSLASIS TSSFVSRRNV
51 VLRRSRLPKI SAAKELHFNK DGSAIKRLQT GVNKLADLVG VTLGPKGRNV
101 VLESKYGSPK IVNDGVTVAK EVELEDPVEN IGAKLVRQAA AKTNDLAGDG
151 TTTSVVLAQG LIAEGVKVVA AGANPVLITR GIEKTSRALV SELKAISKEV
201 EDSELADVAA VSAGNNNEVG NMIAEAMSKV GRKGVVTLEE GK**SAENSLYV**
251 **VEGMQFDRGY ISPYFVTDSE K**MAVEYENCK LLLVDKKITN AR**DLINILED**
301 **AIR**SGYPILI IAEDIEQEAL ATLVVNKLRG ALKIAALKAP GFGER**KSQYL**
351 **DDIAILTGGT VIRDEVGLSL DK**ASKEVLGH ASKVVLTKDT TTIVGDGSTQ
401 EAVNKRVVQI KNLIEAAEQD YEKEKLNERI AKLSGGVAVI QVGAQTETEL
451 KEKKLRVEDA LNATK**AAVEE GIVVGGGCTL LR**LASKVDAI KDSLDNDEEK
501 VGADIVKRAL SYPLKLIAKN AGVNGSVVSE KVLSNDNPRY GFNAATGNYE
551 DLMSAGIIDP TKVVRCCLEH AASVAKTFLM SDCVVVEIKE PEPVPAGNPM
601 DNSGYGY

Spot No.: E14

Protein name: Chaperonin Cpn60/TCP-1 family

Peptide sequences:

K.SAENSLYVVEGMQFDR.G;R.GYISPYFVTDSEK.M;R.DLINILEDAIR.S;R.KSQYLDDIAILTGGTVIR.D;K.AAVEEGIVVGGGCTLLR.L

Accession No.: Gohir.D10G200300.1.p

Mascot score: 340 **Sequence coverage %:** 12

Calculated Mr: 64709 **Calculated *p*I:** 5.72

PFF Searched Score:

Ions score is -10*Log(P), where P is the probability that the observed match is a random event.
Individual ions scores > 34 indicate identity or extensive homology (p<0.05).
Protein scores are derived from ions scores as a non-probabilistic basis for ranking protein hits.

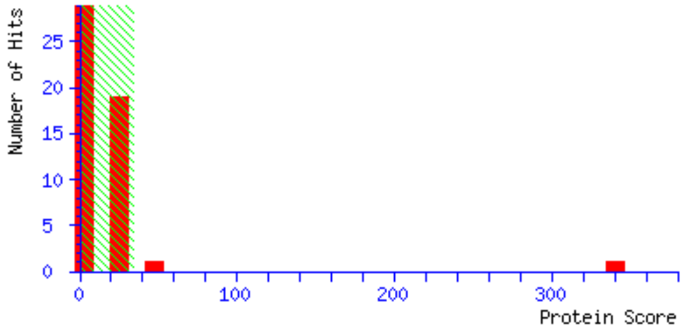

Matched peptide sequences: shown in Bold Red:

1 MASTFTAMSS VGSFVAPNGL VMDKKLSSSS NRLSSLASIS TSSFVSRRNV
51 VLRRSRLPKI SAAKELHFNK DGSAIKRLQT GVNKLADLVG VTLGPKGRNV
101 VLESKYGSPK IVNDGVTVAK EVELEDPVEN IGAKLVRQAA AKTNDLAGDG
151 TTTSVVLAQG LIAEGVKVVA AGANPVLITR GIEKTSRALV SELKAISKEV
201 EDSELADVAA VSAGNNNEVG NMIAEAMSKV GRKGVVTLEE GK**SAENSLYV**
251 **VEGMQFDRGY** **ISPYFVTDSE** **K**MAVEYENCK LLLVDKKITN AR**DLINILED**
301 **AIR**SGYPILI IAEDIEQEAL ATLVVNKLRG ALKIAALKAP GFGER**KSQYL**
351 **DDIAILTGGT** **VIR**DEVGLSL DKASKEVLGH ASKVVLTKDT TTIVGDGSTQ
401 EAVNKRVVQI KNLIEAAEQD YEKEKLNERI AKLSGGVAVI QVGAQTETEL
451 KEKKLRVEDA LNATK**AAVEE** **GIVVGGGCTL** **LR**LASKVDAI KDSLDNDEEK
501 VGADIVKRAL SYPLKLIAKN AGVNGSVVSE KVLSNDNPRY GFNAATGNYE
551 DLMSAGIIDP TKVVRCCLEH AASVAKTFLM SDCVVVEIKE PEPVPAGNPM
601 DNSGYGY

Spot No.: E15

Protein name: Chaperonin-60alpha

Peptide sequences:

K.EIAFDQSSR.A;K.LADAVGLTLGPR.G;R.AIELPNAMENAGAALIR.E;K.LVCEFENAR.V

Accession No.: Gohir.A05G105800.1.p

Mascot score: 97 **Sequence coverage %:** 8

Calculated Mr: 62013 **Calculated *p*I:** 5.26

PFF Searched Score:

Ions score is -10*Log(P), where P is the probability that the observed match is a random event.
Individual ions scores > 34 indicate identity or extensive homology ($p<0.05$).
Protein scores are derived from ions scores as a non-probabilistic basis for ranking protein hits.

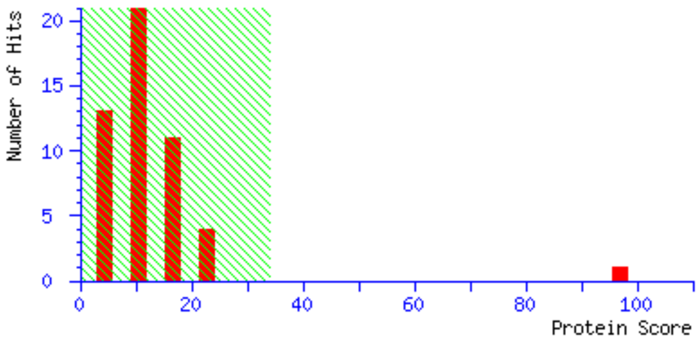

Matched peptide sequences: shown in Bold Red:

```
  1 MASANALSSA SVLCTSKQGS LGRRGNQRQN QRVNYRQGNS RFGVRACAKE
 51 IAFDQSSRAA MQAGIDKLAD AVGLTLGPRG RNVVLDEYGS PKVVNDGVTI
101 ARAIELPNAM ENAGAALIRE VASKTNDSAG DGTTTASILA REIIKLGLLS
151 VTSGANPVSV KRGIDKTVQR LVEELEKKAR PVKGRDDIKA VASVSSGNDD
201 LIGTMIADAI DKVGPDGVLS IESSSSFETT VDVEEGMEID RGYISPQFVT
251 NPEKLVCEFE NARVLVTDQK ISAIKDIIPL LEKTTQLRSP LLIIAEDVSG
301 EALATLVVNK LRGILNVAAI KAPGFGERRK ALLQDIAILT GAEFQASDLG
351 LLVENTSVEQ LGIARKVIIT KDSTQLIAEA ASKDEIQTRV AQLKKELAQT
401 DSVYDSEKLA ERIAKLSGGV AVIKVGAATE TELEDRKLRI EDAKNATFAA
451 IEEGIVPGGG AALVHLSSCV PAIKEKLEDA DERLGADIVQ KALVAPALLI
501 AQNAGMEGEV VVEKVKSSEW EVGYNAMTDK FENLLEAGVI DPAKVTRCAL
551 QNASSVAGMV LTTQAIVVEK PKPKASAAAA PSGLAV
```

Spot No.: E16

Protein name: ATP synthase subunit beta

Peptide sequences:

K.IVNTGTVLQVGDGIAR.I;K.IAQIPVSEAYLGR.V;R.LIESPAPGIISR.R;K.ASSVAQVVTTFQER.G

Accession No.: Gohir.A03G084900.1

Mascot score: 201 **Sequence coverage %:**10

Calculated Mr: 55437 **Calculated *p*I:** 5.25

PFF Searched Score:

Ions score is -10*Log(P), where P is the probability that the observed match is a random event.
Individual ions scores > 35 indicate identity or extensive homology (p<0.05).
Protein scores are derived from ions scores as a non-probabilistic basis for ranking protein hits.

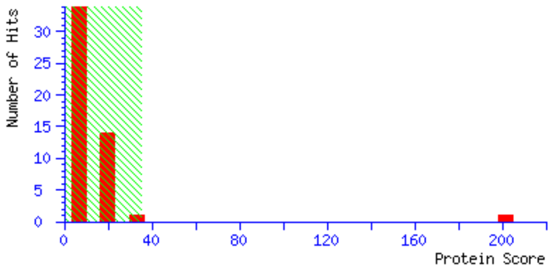

Matched peptide sequences: shown in Bold Red:

1 MVTIRADEIS NIIRERIEQY NREVK**IVNTG TVLQVGDGIA R**IHGLDEVMA
51 GELVEFEEGT IGIALNLESN NVGVVLMGDG LMIQEGSSVK ATGK**IAQIPV**
101 **SEAYLGR**VIN ALAKPIDGRG EISASESR**LI ESPAPGIISR** RSVYEPLQTG
151 LIAIDSMIPI GRGQRELIIG DRQTGKTAVA TDTILNQQGQ NVICVYVAIG
201 QK**ASSVAQVV TTFQER**GAME YTIVVAETAD SPATLQYLAP YTGAALAEYF
251 MYRERHTLII YDDLSKQAQA YRQMSLLLRR PPGREAYPGD VFYLHSRLLE
301 RAAKSSSQLG EGSMTALPIV ETQSGDVSAY IPTNVISITD GQIFLSADLF
351 NAGIRPAINV GISVSRVGSA AQIKAMKQVA GKSKLELAQF AELEAFAQFA
401 SDLDKATQNQ LARGQRLREL LKQSQSAPLT VAEQISTIYT GTNGYLDSLE
451 IGQVRKFLVE LRTYLKTNKP QFQEIISSTK TFTEEAETLL KDAIQDQMER
501 FRLQEQL

Spot No.: E18

Protein name: ATPase V1 complex subunit B protein

Peptide sequences:

K.YQEIVNIR.L;R.VTLFLNLANDPTIER.I;K.FVTQGAYDTR.N

Accession No.: Gohir.A01G109000.1.p

Mascot score: 81 **Sequence coverage %:**6

Calculated Mr: 54369 **Calculated *p*I:** 4.96

PFF Searched Score:

Ions score is -10*Log(P), where P is the probability that the observed match is a random event.
Individual ions scores > 34 indicate identity or extensive homology (p<0.05).
Protein scores are derived from ions scores as a non-probabilistic basis for ranking protein hits.

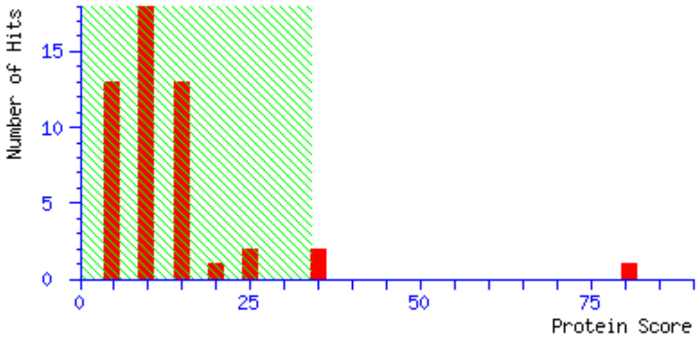

Matched peptide sequences: shown in Bold Red:

```
  1 MGAEKNFIDM EEGTLEIGME YRTVSGVAGP LVILDKVKGP KYQEIVNIRL
 51 GDGTTRRGQV LEVDGEKAVV QVFEGTSGID NKYTTVQFTG EVLKTPVSLD
101 MLGRIFNGSG KPIDNGPPIL PEAYLDISGS SINPSERTYP EEMIQTGIST
151 IDVMNSIARG QKIPLFSAAG LPHNEIAAQI CRQAGLVKRL EKSDNLLDDQ
201 EEDNFAIVFA AMGVNMETAQ FFKRDFEENG SMERVTLFLN LANDPTIERI
251 ITPRIALTTA EYLAYECGKH VLVILTDMSS YADALREVSA AREEVPGRRG
301 YPGYMYTDLA TIYERAGRIE GRKGSITQIP ILTMPNDDIT HPTPDLTGYI
351 TEGQIYIDRQ LHNRQIYPPI NVLPSLSRLM KSAIGEGMTR RDHADVSNQL
401 YANYAIGKDV QAMKAVVGEE ALSSEDLLYL EFLDKFERKF VTQGAYDTRN
451 IFQSLDLAWT LLRIFPRELL HRIPAKTLDQ YYSRES
```

Spot No.: E19

Protein name: ATP synthase subunit alpha

Peptide sequences:

R.ADEISNIIR.E;K.IVNTGTVLQVGDGIAR.I;K.IAQIPVSEAYLGR.V;R.LIESPAPGIISR.R;R.SVYEPLQTGLIAIDSMIPIGR.G;K.ASSVAQVVTTFQER.G;R.KFLVELR.T

Accession No.: Gohir.D01G031800.1.p

Mascot score: 561 **Sequence coverage %:**18

Calculated Mr: 55437 **Calculated *p*I:** 5.25

PFF Searched Score:

Ions score is -10*Log(P), where P is the probability that the observed match is a random event.
Individual ions scores > 35 indicate identity or extensive homology (p<0.05).
Protein scores are derived from ions scores as a non-probabilistic basis for ranking protein hits.

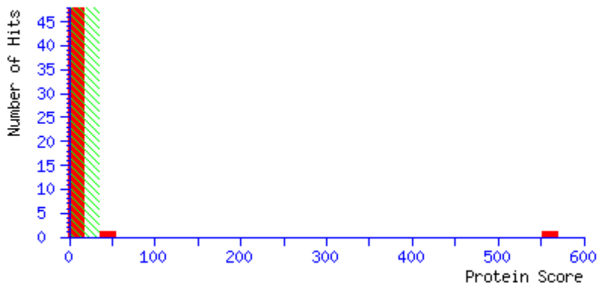

Matched peptide sequences: shown in Bold Red:

```
  1 MVTIRADEIS NIIRERIEQY NREVKIVNTG TVLQVGDGIA RIHGLDEVMA
 51 GELVEFEEGT IGIALNLESN NVGVVLMGDG LMIQEGSSVK ATGKIAQIPV
101 SEAYLGRVIN ALAKPIDGRG EISASESRLI ESPAPGIISR RSVYEPLQTG
151 LIAIDSMIPI GRGQRELIIG DRQTGKTAVA TDTILNQQGQ NVICVYVAIG
201 QKASSVAQVV TTFQERGAME YTIVVAETAD SPATLQYLAP YTGAALAEYF
251 MYRERHTLII YDDLSKQAQA YRQMSLLLRR PPGREAYPGD VFYLHSRLLE
301 RAAKSSSQLG EGSMTALPIV ETQSGDVSAY IPTNVISITD GQIFLSADLF
351 NAGIRPAINV GISVSRVGSA AQIKAMKQVA GKSKLELAQF AELEAFAQFA
401 SDLDKATQNQ LARGQRLREL LKQSQSAPLT VAEQISTIYT GTNGYLDSLE
451 IGQVRKFLVE LRTYLKTNKP QFQEIISSTK TFTEEAETLL KDAIQDQMER
501 FRLQEQL
```

Spot No.: E20

Protein name: ATP synthase subunit alpha

Peptide sequences:

K.IVNTGTVLQVGDGIAR.I;K.IAQIPVSEAYLGR.V;R.LIESPAPGIISR.R;R.SVYEPLQTGLIAIDSMIPIGR.G;K.ASSVAQVVTTFQER.G;K.QSQSAPLTVAEQISTIYTGTNGYLDSLEIGQVR.K

Accession No.: Gohir.D01G031800.1.p

Mascot score: 542 **Sequence coverage %:**21

Calculated Mr: 55437 **Calculated *p*I:** 5.25

PFF Searched Score:

Ions score is -10*Log(P), where P is the probability that the observed match is a random event.
Individual ions scores > 34 indicate identity or extensive homology (p<0.05).
Protein scores are derived from ions scores as a non-probabilistic basis for ranking protein hits.

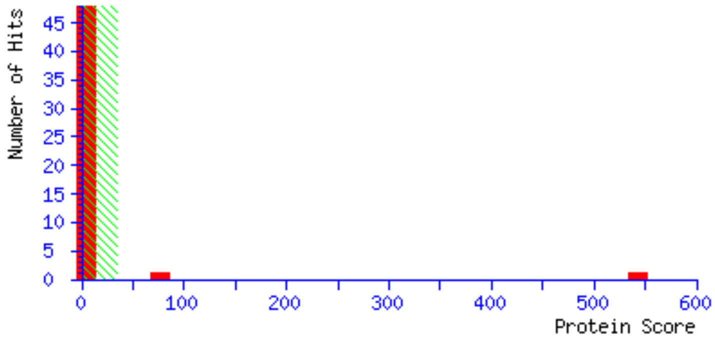

Matched peptide sequences: shown in Bold Red:

```
  1 MVTIRADEIS NIIRERIEQY NREVKIVNTG TVLQVGDGIA RIHGLDEVMA
 51 GELVEFEEGT IGIALNLESN NVGVVLMGDG LMIQEGSSVK ATGKIAQIPV
101 SEAYLGRVIN ALAKPIDGRG EISASESRLI ESPAPGIISR RSVYEPLQTG
151 LIAIDSMIPI GRGQRELIIG DRQTGKTAVA TDTILNQQGQ NVICVYVAIG
201 QKASSVAQVV TTFQERGAME YTIVVAETAD SPATLQYLAP YTGAALAEYF
251 MYRERHTLII YDDLSKQAQA YRQMSLLLRR PPGREAYPGD VFYLHSRLLE
301 RAAKSSSQLG EGSMTALPIV ETQSGDVSAY IPTNVISITD GQIFLSADLF
351 NAGIRPAINV GISVSRVGSA AQIKAMKQVA GKSKLELAQF AELEAFAQFA
401 SDLDKATQNQ LARGQRLREL LKQSQSAPLT VAEQISTIYT GTNGYLDSLE
451 IGQVRKFLVE LRTYLKTNKP QFQEIISSTK TFTEEAETLL KDAIQDQMER
501 FRLQEQL
```

Spot No.: E21

Protein name: ATP synthase subunit alpha

Peptide sequences:

R.ADEISNIIR.E;K.IVNTGTVLQVGDGIAR.I;K.IAQIPVSEAYLGR.V;R.LIESPAPGIISR.R;R.SVYEPLQTGLIAIDSMIPIGR.G;K.ASSVAQVVTTFQER.G;K.ATQNQLAR.G

Accession No.: Gohir.D01G031800.1.p

Mascot score: 449 **Sequence coverage %:**18

Calculated Mr: 55437 **Calculated *p*I:** 5.25

PFF Searched Score:

Ions score is -10*Log(P), where P is the probability that the observed match is a random event.
Individual ions scores > 35 indicate identity or extensive homology (p<0.05).
Protein scores are derived from ions scores as a non-probabilistic basis for ranking protein hits.

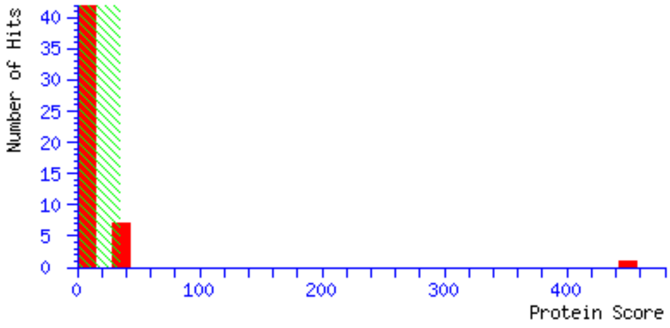

Matched peptide sequences: shown in Bold Red:

1 MVTIR**ADEIS** **NIIR**ERIEQY NREVK**IVNTG** **TVLQVGDGIA** **R**IHGLDEVMA
51 GELVEFEEGT IGIALNLESN NVGVVLMGDG LMIQEGSSVK ATGK**IAQIPV**
101 **SEAYLGR**VIN ALAKPIDGRG EISASESR**LI** **ESPAPGIISR** **RSVYEPLQTG**
151 **LIAIDSMIPI** **GR**GQRELIIG DRQTGKTAVA TDTILNQQGQ NVICVYVAIG
201 QK**ASSVAQVV** **TTFQER**GAME YTIVVAETAD SPATLQYLAP YTGAALAEYF
251 MYRERHTLII YDDLSKQAQA YRQMSLLLRR PPGREAYPGD VFYLHSRLLE
301 RAAKSSSQLG EGSMTALPIV ETQSGDVSAY IPTNVISITD GQIFLSADLF
351 NAGIRPAINV GISVSRVGSA AQIKAMKQVA GKSKLELAQF AELEAFAQFA
401 SDLDK**ATQNQ** **LAR**GQRLREL LKQSQSAPLT VAEQISTIYT GTNGYLDSLE
451 IGQVRKFLVE LRTYLKTNKP QFQEIISSTK TFTEEAETLL KDAIQDQMER
501 FRLQEQL

Spot No.: E22

Protein name: ATP synthase subunit beta

Peptide sequences:

R.GMEVIDTGAALSVPVGGATLGR.I;K.VVDLLAPYR.R;K.AHGGVSVFGGVGER.T;K.VALVYGQMNEPPGAR.M;R.VGLTALTMAEYFR.D;R.DVNEQDVLLFIDNIFR.F;R.FVQAGSEVSALLGR.M

Accession No.: Gohir.A03G084900.1.p

Mascot score: 660 **Sequence coverage %:** 20

Calculated Mr: 53611 **Calculated *p*I:** 5.22

PFF Searched Score:

Ions score is -10*Log(P), where P is the probability that the observed match is a random event.
Individual ions scores > 34 indicate identity or extensive homology (p<0.05).
Protein scores are derived from ions scores as a non-probabilistic basis for ranking protein hits.

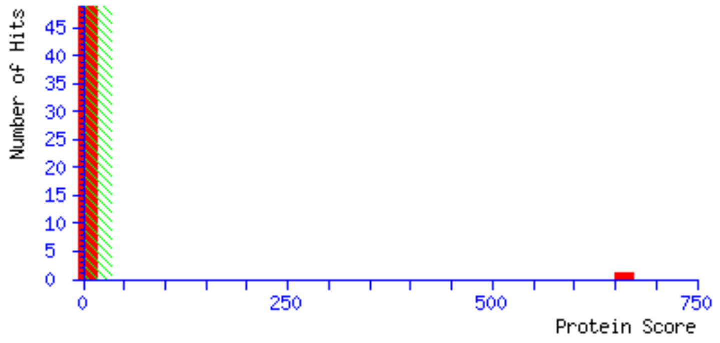

Matched peptide sequences: shown in Bold Red:

```
  1 MKINPTTSVP GVSTLEKENL GRISQIIGPV LDVAFPPGKM PNIYNALVVK
 51 GQDTAGQQIN VTCEVQQLLG NNRVRAVAMS ATDGLTRGME VIDTGAALSV
101 PVGGATLGRI FNVLGEPVDN LGPVDTRTTS PIHKSAPAFI QLDTKLSIFE
151 TGIKVVDLLA PYRRGGKIGL FGGAGVGKTV LIMELINNIA KAHGGVSVFG
201 GVGERTREGN DLYMEMKESG VINEQNLAES KVALVYGQMN EPPGARMRVG
251 LTALTMAEYF RDVNEQDVLL FIDNIFRFVQ AGSEVSALLG RMPSAVGYQP
301 TLSTEMGTLQ ERITSTKEGS ITSIQAVYVP ADDLTDPAPA TTFAHLDATT
351 VLSRGLAAKG IYPAVDPLDS TSTMLQPRIV GEEHYETAQR VKQTLQRYKE
401 LQDIIAILGL DELSEEDRLT VARARKIERF LSQPFFVAEV FTGSPGKYVG
451 LAETIRGFKL ILSGELDGLP EQAFYLVGNI DEATAKATNL EMESKLKK
```

Spot No.: E23

Protein name: ATP synthase subunit beta

Peptide sequences:

R.GMEVIDTGAALSVPVGGATLGR.I;K.VVDLLAPYR.R;K.AHGGVSVFGGVGER.T;K.VALVYGQMNEPPGAR.M;R.VGLTALTMAEYFR.D;R.DVNEQDVLLFIDNIFR.F;R.FVQAGSEVSALLGR.M

Accession No.: Gohir.A03G084900.1.p

Mascot score: 743 **Sequence coverage %:**20

Calculated Mr: 53611 **Calculated *p*I:** 5.22

PFF Searched Score:

Ions score is -10*Log(P), where P is the probability that the observed match is a random event.
Individual ions scores > 34 indicate identity or extensive homology (p<0.05).
Protein scores are derived from ions scores as a non-probabilistic basis for ranking protein hits.

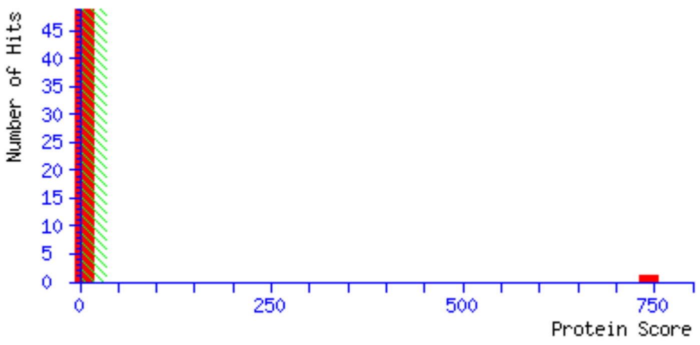

Matched peptide sequences: shown in Bold Red:

1 MKINPTTSVP GVSTLEKENL GRISQIIGPV LDVAFPPGKM PNIYNALVVK
51 GQDTAGQQIN VTCEVQQLLG NNRVRAVAMS ATDGLTR**GME** **VIDTGAALSV**
101 **PVGGATLGR**I FNVLGEPVDN LGPVDTRTTS PIHKSAPAFI QLDTKLSIFE
151 TGIK**VVDLLA** **PYR**RGGKIGL FGGAGVGKTV LIMELINNIA K**AHGGVSVFG**
201 **GVGER**TREGN DLYMEMKESG VINEQNLAES K**VALVYGQMN** **EPPGAR**MRVG
251 **LTALTMAEYF** R**DVNEQDVLL** **FIDNIFR**FVQ AGSEVSALLG RMPSAVGYQP
301 TLSTEMGTLQ ERITSTKEGS ITSIQAVYVP ADDLTDPAPA TTFAHLDATT
351 VLSRGLAAKG IYPAVDPLDS TSTMLQPRIV GEEHYETAQR VKQTLQRYKE
401 LQDIIAILGL DELSEEDRLT VARARKIERF LSQPFFVAEV FTGSPGKYVG
451 LAETIRGFKL ILSGELDGLP EQAFYLVGNI DEATAKATNL EMESKLKK

Spot No.: E24

Protein name: Tubulin alpha-3

Peptide sequences:

R.AVFVDLEPTVIDEVR.T;R.LISQTISSLTTSLR.F;R.FDGAINVDITEFQTNLVPYPR.I;R.AVCMISNNTAVAEVFAR.I;R.AFVHWYVGEGMEEGEFSEAR.E

Accession No.: Gohir.A11G076100.1

Mascot score: 369 **Sequence coverage %:** 19

Calculated Mr: 50264 **Calculated *p*I:** 5.01

PFF Searched Score:

Ions score is -10*Log(P), where P is the probability that the observed match is a random event.
Individual ions scores > 33 indicate identity or extensive homology (p<0.05).
Protein scores are derived from ions scores as a non-probabilistic basis for ranking protein hits.

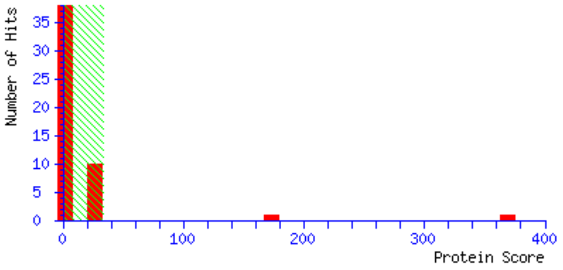

Matched peptide sequences: shown in Bold Red:

```
  1 MREIISVHIG QAGIQVGNSC WELYCLEHVI HPDGTMPSDT SMGVAHDAFN
 51 TFFSETGSGK HVPRAVFVDL EPTVIDEVRT GPYRQLFHPE QLISGKEDAA
101 NNFARGHYTI GKEIVDLCLD RVRKLADNCT GLQGFMVFNA VGGGTGSGLG
151 SLLLERLSVD YGKKSKLGFT IYPSPQVSTA VVEPYNSVLS THSLLEHTDV
201 AVLLDNEAIY DICRRSLDIE RPTYTNLNRL ISQTISSLTT SLRFDGAINV
251 DITEFQTNLV PYPRIHFMLS SYAPVISAEK AYHEQISVPE ITNAVFEPSS
301 MMAKCDPRHG KYMACCLMYR GDVVPKDVNA AVATIKTKRT VQFVDWCPTG
351 FKCGINYQPP AVVPGGDLAK VQRAVCMISN NTAVAEVFAR IDHKFDLMYS
401 KRAFVHWYVG EGMEEGEFSE AREDLAALEK DYEEVGAEGG DDEEEGEEY
```

Spot No.: F3

Protein name: **Ribulose bisphosphate carboxylase**

Peptide sequences:

K.GLAYDISDDQQDITR.G;K.MCALFINDLDAGAGR.M;R.VPIIVTGNDFSTLYAPLIR.D;K.FYWAPTR.D;K.LVDTFPGQSIDFFGALR.A;K.LLEYGNMLVAEQENVKR.V

Accession No.: **Gohir.A06G166200.1.p**

Mascot score: **674** **Sequence coverage %:** **19**

Calculated Mr: **51953** **Calculated *p*I:** **5.37**

PFF Searched Score:

Ions score is -10*Log(P), where P is the probability that the observed match is a random event.
Individual ions scores > 34 indicate identity or extensive homology (p<0.05).
Protein scores are derived from ions scores as a non-probabilistic basis for ranking protein hits.

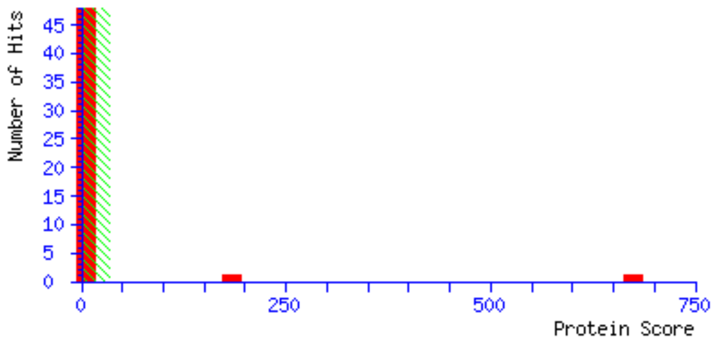

Matched peptide sequences: shown in Bold Red:

1 MAAAISTVAA AAAANRALGV SVPTSAFMGK KVSSRFNNTR QVPSGSFKVM
51 AAEKEIDEET QTEKDRWK**GL** **AYDISDDQQD** **ITR**GKGMVDS LFQAPMNDGT
101 HYAVMSSYEY ISQGLKTYNL DNNMDGFYIA PAFMDKLVVH ISKNFMSLPN
151 IKVPLILGIW GGKGQGKSFQ CELVFAKMGI NPIMMSAGEL ESGNAGEPAK
201 LIRQRYREAA DIIKKGK**MCA** **LFINDLDAGA** **GR**MGGTTQYT VNNQMVNATL
251 MNIADNPTNV QLPGMYNKEE NPR**VPIIVTG** **NDFSTLYAPL** **IR**DGRMEK**FY**
301 **WAPTR**DDRIG VCKGIFRTDG VRDEDIVK**LV** **DTFPGQSIDF** **FGALR**ARVYD
351 DEVRKWISEV GVASVGKKLV NSREGPPTFE QPKMTIEK**LL** **EYGNMLVAEQ**
401 **ENVKR**VQLAD KYLSEAALGE ANEDSINRGT FYGKAAQQVG VPVPEGCTDP
451 NADNFDPTAR SDDGTCTYKF

Spot No.: F4

Protein name: Regulatory particle triple-A ATPase 5A

Peptide sequences:

K.DSYLILDTLPSEYDSR.V;R.KIEFPHPTEEAR.A

Accession No.: Gohir.A11G294500.1.p

Mascot score: 113 **Sequence coverage %:**6

Calculated Mr: 47690 **Calculated *p*I:** 4.99

PFF Searched Score:

Ions score is -10*Log(P), where P is the probability that the observed match is a random event.
Individual ions scores > 34 indicate identity or extensive homology (p<0.05).
Protein scores are derived from ions scores as a non-probabilistic basis for ranking protein hits.

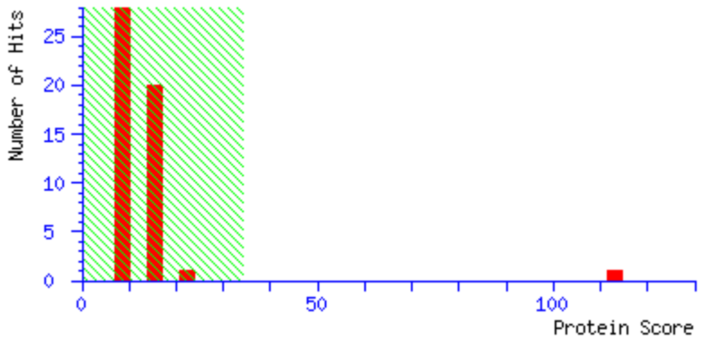

Matched peptide sequences: shown in Bold Red:

```
  1 MATAMVEDSS FEEDQLASMT TEDIVRASRL LDNEIRILKE EMQRTNLELD
 51 SYKEKVKENQ EKIKLNKQLP YLVGNIVEIL EMNPEDEAEE DGANIDLDSQ
101 RKGKCVVLKT STRQTIFLPV VGLVDPDKLK PGDLVGVNKD SYLILDTLPS
151 EYDSRVKAME VDEKPTEDYN DIGGLEKQIQ ELVEAIVLPM THKERFQKLG
201 VRPPKGVLLY GPPGTGKTLM ARACAAQTNA TFLKLAGPQL VQMFIGDGAK
251 LVRDAFQLAK EKSPCIIFID EIDAIGTKRF DSEVSGDREV QRTMLELLNQ
301 LDGFSSDERI KVIAATNRAD ILDPALMRSG RLDRKIEFPH PTEEARARIL
351 QIHSRKMNVH PDVNFEELAR STDDFNGAQL KAVCVEAGML ALRRDATEVN
401 HEDFNEGIIQ VQAKKKASLN YYA
```

Spot No.: F5

Protein name: Ribulose bisphosphate carboxylase

Peptide sequences:

K.GLAYDISDDQQDITR.G;K.MCALFINDLDAGAGR.M;R.VPIIVTGNDFSTLYAPLIR.D;K.FYWAPTR.D;K.LVDTFPGQSIDFFGALR.A;K.LLEYGNMLVAEQENVKR.V

Accession No.: Gohir.A06G166200.1

Mascot score: 686 **Sequence coverage %:**19

Calculated Mr: 51953 **Calculated *p*I:** 5.37

PFF Searched Score:

Ions score is -10*Log(P), where P is the probability that the observed match is a random event.
Individual ions scores > 34 indicate identity or extensive homology (p<0.05).
Protein scores are derived from ions scores as a non-probabilistic basis for ranking protein hits.

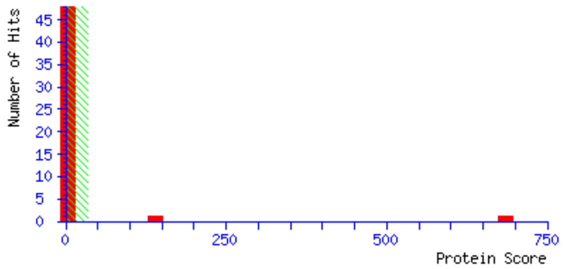

Matched peptide sequences: shown in Bold Red:

```
  1 MAAAISTVAA AAAANRALGV SVPTSAFMGK KVSSRFNNTR QVPSGSFKVM
 51 AAEKEIDEET QTEKDRWKGL AYDISDDQQD ITRGKGMVDS LFQAPMNDGT
101 HYAVMSSYEY ISQGLKTYNL DNNMDGFYIA PAFMDKLVVH ISKNFMSLPN
151 IKVPLILGIW GGKGQGKSFQ CELVFAKMGI NPIMMSAGEL ESGNAGEPAK
201 LIRQRYREAA DIIKKGKMCA LFINDLDAGA GRMGGTTQYT VNNQMVNATL
251 MNIADNPTNV QLPGMYNKEE NPRVPIIVTG NDFSTLYAPL IRDGRMEKFY
301 WAPTRDDRIG VCKGIFRTDG VRDEDIVKLV DTFPGQSIDF FGALRARVYD
351 DEVRKWISEV GVASVGKKLV NSREGPPTFE QPKMTIEKLL EYGNMLVAEQ
401 ENVKRVQLAD KYLSEAALGE ANEDSINRGT FYGKAAQQVG VPVPEGCTDP
451 NADNFDPTAR SDDGTCTYKF
```

Spot No.: F6

Protein name: Ribulose bisphosphate carboxylase

Peptide sequences:

K.GLAYDISDDQQDITR.G;K.MCALFINDLDAGAGR.M;R.VPIIVTGNDFSTLYAPLIR.D;K.FYWAPTR.D;K.LVDTFPGQSIDFFGALR.A;K.LLEYGNMLVAEQENVKR.V

Accession No.: Gohir.A06G166200.1.p

Mascot score: 721 **Sequence coverage %:**19

Calculated Mr: 51953 **Calculated *p*I:** 5.37

PFF Searched Score:

Ions score is -10*Log(P), where P is the probability that the observed match is a random event.
Individual ions scores > 34 indicate identity or extensive homology (p<0.05).
Protein scores are derived from ions scores as a non-probabilistic basis for ranking protein hits.

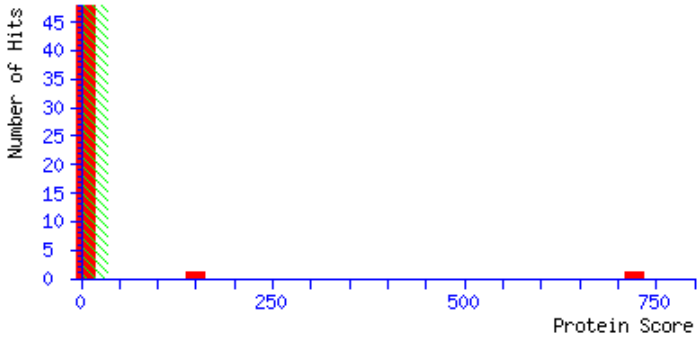

Matched peptide sequences: shown in Bold Red:

```
  1 MAAAISTVAA AAAANRALGV SVPTSAFMGK KVSSRFNNTR QVPSGSFKVM
 51 AAEKEIDEET QTEKDRWKGL AYDISDDQQD ITRGKGMVDS LFQAPMNDGT
101 HYAVMSSYEY ISQGLKTYNL DNNMDGFYIA PAFMDKLVVH ISKNFMSLPN
151 IKVPLILGIW GGKGQGKSFQ CELVFAKMGI NPIMMSAGEL ESGNAGEPAK
201 LIRQRYREAA DIIKKGKMCA LFINDLDAGA GRMGGTTQYT VNNQMVNATL
251 MNIADNPTNV QLPGMYNKEE NPRVPIIVTG NDFSTLYAPL IRDGRMEKFY
301 WAPTRDDRIG VCKGIFRTDG VRDEDIVKLV DTFPGQSIDF FGALRARVYD
351 DEVRKWISEV GVASVGKKLV NSREGPPTFE QPKMTIEKLL EYGNMLVAEQ
401 ENVKRVQLAD KYLSEAALGE ANEDSINRGT FYGKAAQQVG VPVPEGCTDP
451 NADNFDPTAR SDDGTCTYKF
```

Spot No.: F7

Protein name: Ribulose bisphosphate carboxylase

Peptide sequences:

K.GLAYDISDDQQDITR.G;K.MCALFINDLDAGAGR.M;R.VPIIVTGNDFSTLYAPLIR.D;K.FYWAPTR.D;K.LVDTFPGQSIDFFGALR.A;K.LLEYGNMLVAEQENVKR.V

Accession No.: Gohir.A06G166200.1.p

Mascot score: 605 **Sequence coverage %:**19

Calculated Mr: 51953 **Calculated *p*I:** 5.37

PFF Searched Score:

Ions score is -10*Log(P), where P is the probability that the observed match is a random event.
Individual ions scores > 34 indicate identity or extensive homology (p<0.05).
Protein scores are derived from ions scores as a non-probabilistic basis for ranking protein hits.

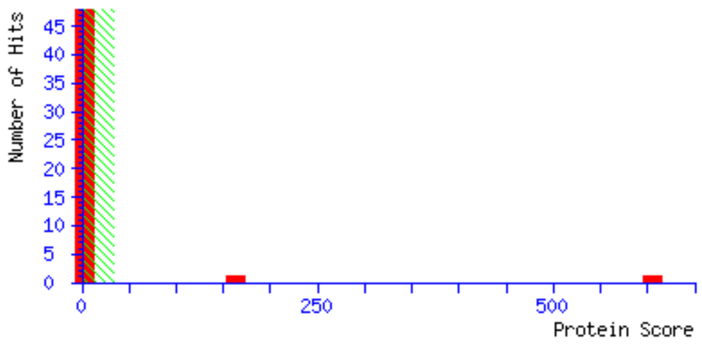

Matched peptide sequences: shown in Bold Red:

1 MAAAISTVAA AAAANRALGV SVPTSAFMGK KVSSRFNNTR QVPSGSFKVM
51 AAEKEIDEET QTEKDRWK**GL** **AYDISDDQQD** **ITR**GKGMVDS LFQAPMNDGT
101 HYAVMSSYEY ISQGLKTYNL DNNMDGFYIA PAFMDKLVVH ISKNFMSLPN
151 IKVPLILGIW GGKGQGKSFQ CELVFAKMGI NPIMMSAGEL ESGNAGEPAK
201 LIRQRYREAA DIIKKGK**MCA** **LFINDLDAGA** **GR**MGGTTQYT VNNQMVNATL
251 MNIADNPTNV QLPGMYNKEE NPR**VPIIVTG** **NDFSTLYAPL** **IR**DGRMEK**FY**
301 **WAPTR**DDRIG VCKGIFRTDG VRDEDIVK**LV** **DTFPGQSIDF** **FGALR**ARVYD
351 DEVRKWISEV GVASVGKKLV NSREGPPTFE QPKMTIEK**LL** **EYGNMLVAEQ**
401 **ENVKR**VQLAD KYLSEAALGE ANEDSINRGT FYGKAAQQVG VPVPEGCTDP
451 NADNFDPTAR SDDGTCTYKF

Spot No.: F9

Protein name: S-adenosylmethionine synthetase 2

Peptide sequences:

K.VLVNIEQQSPDIAQGVHGHFTK.R;K.TIFHLNPSGR.F;R.FVIGGPHGDAGLTGR.K;K.ENFDFRPGMITINLDLK.R;K.TAAYGHFGR.D

Accession No.: Gohir.D07G135100.1.p

Mascot score: 329 **Sequence coverage %:** 18

Calculated Mr: 43500 **Calculated *p*I:** 5.49

PFF Searched Score:

Ions score is -10*Log(P), where P is the probability that the observed match is a random event.
Individual ions scores > 35 indicate identity or extensive homology (p<0.05).
Protein scores are derived from ions scores as a non-probabilistic basis for ranking protein hits.

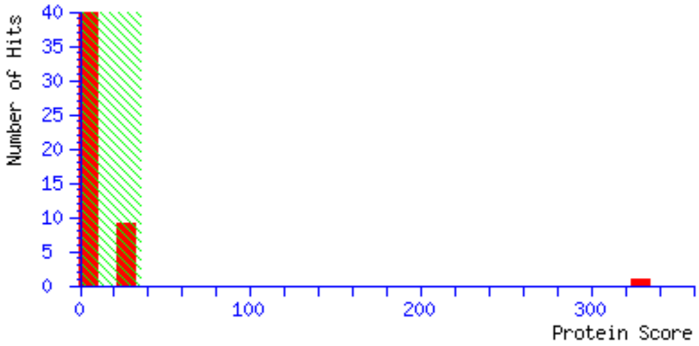

Matched peptide sequences: shown in Bold Red:

```
  1 METFLFTSES VNEGHPDKLC DQVSDAVLDA CLAQDPDSKV ACETCTKTNM
 51 VMVFGEITTK ANVDYEKIVR DTCRSIGFVS DDVGLDADNC KVLVNIEQQS
101 PDIAQGVHGH FTKRPEEIGA GDQGHMFGYA TDETPEFMPL SHVLATKLGA
151 RLTDVRKNGT CPWLRPDGKT QVTVEYYNDN GAMVPVRVHT VLISTQHDET
201 VTNDEIAADL KEHVIKPVIP EKYLDEKTIF HLNPSGRFVI GGPHGDAGLT
251 GRKIIIDTYG GWGAHGGGAF SGKDPTKVDR SGAYIVRQAA KSIVANGLAR
301 RCIVQVSYAI GVPEPLSVFV DSYGTGKIPD KEILQIVKEN FDFRPGMITI
351 NLDLKRGGNS RFLKTAAYGH FGRDDPDFTW EAVKPLKWDK PQS
```

Spot No.: F14

Protein name: Actin 7

Peptide sequences:

R.AVFPSIVGR.P;K.IWHHTFYNELR.V;K.NYELPDGQVITIGAER.F;K.GEYDESGPSIVHR.K

Accession No.: Gohir.A03G105800.1.p

Mascot score: 175 **Sequence coverage %:**12

Calculated Mr: 41913 **Calculated *p*I:** 5.31

PFF Searched Score:

Ions score is -10*Log(P), where P is the probability that the observed match is a random event.
Individual ions scores > 34 indicate identity or extensive homology (p<0.05).
Protein scores are derived from ions scores as a non-probabilistic basis for ranking protein hits.

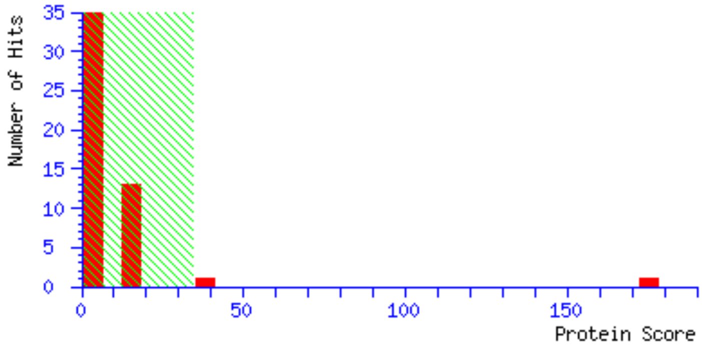

Matched peptide sequences: shown in Bold Red:

1 MADAEDIQPL VCDNGTGMVK AGFAGDDAPR **AVFPSIVGR**P RHTGVMVGMG
51 QKDAYVGDEA QSKRGILTLK YPIEHGIVSN WDDMEK**IWHH TFYNELR**VAP
101 EEHPVLLTEA PLNPKANREK MTQIMFETFN VPAMYVAIQA VLSLYASGRT
151 TGIVLDSGDG VSHTVPIYEG YALPHAILRL DLAGRDLTDS LMKILTERGY
201 MFTTTAEREI VRDMKEKLAY VALDYEQELE TAKSSSSVEK **NYELPDGQVI**
251 **TIGAER**FRCP EVLFQPSLIG MEAAGIHETT YNSIMKCDVD IRKDLYGNIV
301 LSGGSTMFPG IADRMSKEIT ALAPSSMKIK VVAPPERKYS VWIGGSILAS
351 LSTFQQMWIS K**GEYDESGPS IVHR**KCF

Spot No.: F15

Protein name: RAB GTPase homolog E1B

Peptide sequences:

K.KYDEIDAAPEER.A;R.GITINTATVEYETENR.H;K.IYELMDAVDSYIPVPQR.Q;K.TLDDAMAGDNVGLLLR.G

Accession No.: Gohir.A03G057300.1.p

Mascot score: 372 **Sequence coverage %:**12

Calculated Mr: 52793 **Calculated** ***p*I:** 6.21

PFF Searched Score:

Ions score is -10*Log(P), where P is the probability that the observed match is a random event.
Individual ions scores > 34 indicate identity or extensive homology (p<0.05).
Protein scores are derived from ions scores as a non-probabilistic basis for ranking protein hits.

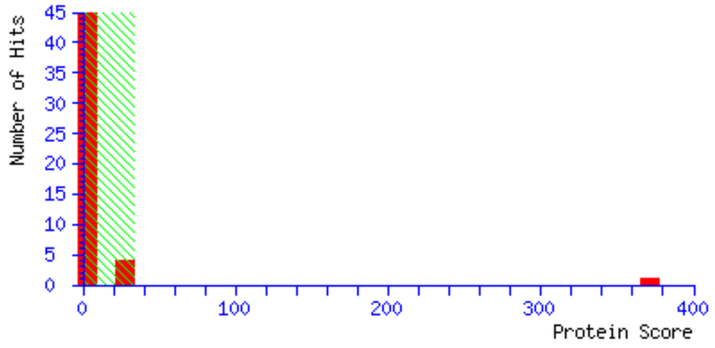

Matched peptide sequences: shown in Bold Red:

```
  1 MAISSTAAAT AASSKLRYPH ASPFPTHSST TTSAFFSSIP SKLTPTHLSS
 51 SFLPPFLTTV ATTYVFPRRR DSFTVKAARG KFERKKPHVN IGTIGHVDHG
101 KTTLTAALTM ALASMGNSAP KKYDEIDAAP EERARGITIN TATVEYETEN
151 RHYAHVDCPG HADYVKNMIT GAAQMDGAIL VVSGADGPMP QTKEHILLAK
201 QVGVPNVVVF LNKQDQVDDE ELLQLVELEV RELLSSYEFP GDDVPIISGS
251 ALLALEALMA KPSIPRGENQ WVDKIYELMD AVDSYIPVPQ RQTDLPFLLA
301 VEDVFSITGR GTVATGRIER GTVKVGETVD IVGLKDTRNV TVTGVEMFQK
351 TLDDAMAGDN VGLLLRGVQK ADIQRGMVLA KPGTITPHTK FSAIVYVLKK
401 EEGGRHSPFF AGYRPQFYMR TTDVTGRVAS IMNDKDEESK MVMPGDRVKM
451 VVELIMPVAC EQGMRFAIRE GGKTVGAGVI QSIIE
```

Spot No.: F16

Protein name: Glutamine synthetase

Peptide sequences:

R.GGNNILVICDSYTPAGEPIPTNKR.H;R.HKEHISAYGEGNER.R;K.EHISAYGEGNER.R;K.HETASINTFSWGVANR.G;R.RPASNMDPYVVTALLAETTLLYEPTLEAEALAAQK.I

Accession No.: Gohir.D02G032300.1

Mascot score: 431 **Sequence coverage %:**20

Calculated Mr: 47737 **Calculated *p*I:** 7.08

PFF Searched Score:

Ions score is -10*Log(P), where P is the probability that the observed match is a random event.
Individual ions scores > 34 indicate identity or extensive homology (p<0.05).
Protein scores are derived from ions scores as a non-probabilistic basis for ranking protein hits.

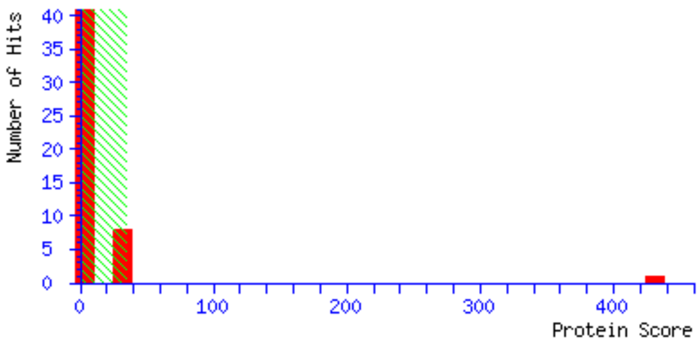

Matched peptide sequences: shown in Bold Red:

```
  1 MAQLLAPSTQ WQMTLPKTST YGSPIATKMW SSLVLKQNKK GAAKSSGKFK
 51 VVALSENSTV NRLENLLNMD VTPYTDKIIA EYIWIGGSGL DMRSKSRTIS
101 KPVKHPSELP KWNYDGSSTG QAPGEDSEVI LYPQAIFKDP FRGGNNILVI
151 CDSYTPAGEP IPTNKRHRAA EIFSNKKVVD EVPWFGIEQE YTLLQQNVKW
201 PLGWPVGGYP GPQGPYYCAA GADKSFGRDI SDAHYKACLY AGINISGTNG
251 EVMPGQWEYQ VGPSVGIEAG DHIWCSRYIL ERITEQAGVV LSLDPKPIEG
301 DWNGAGCHTN YSTKSMREDG GFEVIKKAIL NLSLRHKEHI SAYGEGNERR
351 LTGKHETASI NTFSWGVANR GCSIRVGRDT EKNGKGYLED RRPASNMDPY
401 VVTALLAETT LLYEPTLEAE ALAAQKIALN V
```

Spot No.: F19

Protein name: Rubisco activase

Peptide sequences:

K.GLAYDISDDQQDITR.G;R.VPIIVTGNDFSTLYAPLIR.D;K.FYWAPTR.E;R.IGVCTGIFR.T;K.LVDTFPGQSIDFFGALR.A;K.YLSEAALGNANDDAIKR.G

Accession No.: Gohir.A10G221700.1.p

Mascot score: 625 **Sequence coverage %:**19

Calculated Mr: 48189 **Calculated *p*I:** 5.40

PFF Searched Score:

Ions score is -10*Log(P), where P is the probability that the observed match is a random event.
Individual ions scores > 34 indicate identity or extensive homology (p<0.05).
Protein scores are derived from ions scores as a non-probabilistic basis for ranking protein hits.

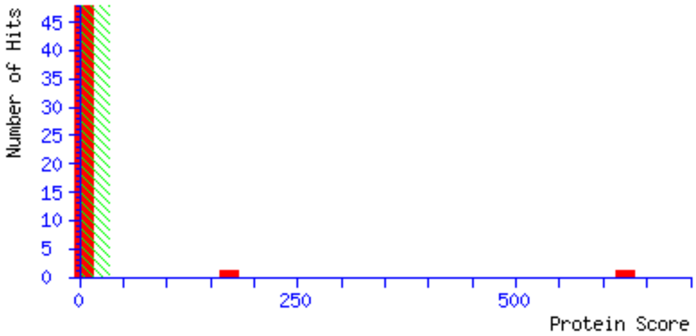

Matched peptide sequences: shown in Bold Red:

1 MAAAVSTIGA VNRAPLSLNG SGAGASAPSS AFMGNSLKKV SARFNNNGKA
51 PVGSFMIVAA KEIDEDTQTD QDRWK**GLAYD ISDDQQDITR** GKGMVDSLFQ
101 APMNDGTHYA VMSSYEYISQ GLRTYDLDNN MDGFYIAPAF MDKLVVHITK
151 NYMTLPNIKV PLILGIWGGK GQGKSFQCEL VFAKMGINPI MMSAGELESG
201 NAGEPAKLIR QRYREAADII KKGKMCCLFI NDLDAGAGRM GGTTQYTVNN
251 QMVNATLMNI ADNPTNVQLP GMYNKEENPR **VPIIVTGNDF STLYAPLIR**D
301 GRMEK**FYWAP TR**EDR**IGVCT GIFR**TDNVPV DDIVK**LVDTF PGQSIDFFGA**
351 **LR**ARVYDDEV RKWIGEVGVN SVGKKLVNSR EGPPSFEQPT MTIEKLLEYG
401 NMLVAEQENV KRVQLADK**YL SEAALGNAND DAIKR**GAF

Spot No.: F21

Protein name: Rubisco activase

Peptide sequences:

K.GLAYDISDDQQDITR.G;R.VPIIVTGNDFSTLYAPLIR.D;K.FYWAPTR.E;R.IGVCTGIFR.T;K.LVDTFPGQSIDFFGALR.A;K.YLSEAALGNANDDAIKR.G

Accession No.: Gohir.A10G221700.1.p

Mascot score: 669 **Sequence coverage %:**19

Calculated Mr: 48189 **Calculated *p*I:** 5.40

PFF Searched Score:

Ions score is -10*Log(P), where P is the probability that the observed match is a random event.
Individual ions scores > 34 indicate identity or extensive homology (p<0.05).
Protein scores are derived from ions scores as a non-probabilistic basis for ranking protein hits.

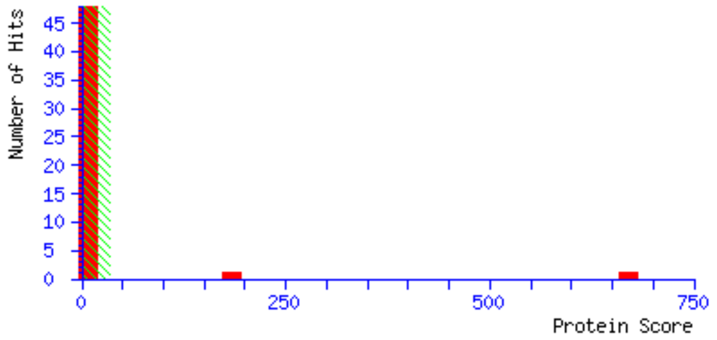

Matched peptide sequences: shown in Bold Red:

1 MAAAVSTIGA VNRAPLSLNG SGAGASAPSS AFMGNSLKKV SARFNNNGKA

51 PVGSFMIVAA KEIDEDTQTD QDRWK**GLAYD** **ISDDQQDITR** GKGMVDSLFQ

101 APMNDGTHYA VMSSYEYISQ GLRTYDLDNN MDGFYIAPAF MDKLVVHITK

151 NYMTLPNIKV PLILGIWGGK GQGKSFQCEL VFAKMGINPI MMSAGELESG

201 NAGEPAKLIR QRYREAADII KKGKMCCLFI NDLDAGAGRM GGTTQYTVNN

251 QMVNATLMNI ADNPTNVQLP GMYNKEENPR **VPIIVTGNDF** **STLYAPLIR**D

301 GRMEK**FYWAP** **TR**EDR**IGVCT** **GIFR**TDNVPV DDIVK**LVDTF** **PGQSIDFFGA**

351 **LR**ARVYDDEV RKWIGEVGVN SVGKKLVNSR EGPPSFEQPT MTIEKLLEYG

401 NMLVAEQENV KRVQLADK**YL** **SEAALGNAND** **DAIKR**GAF

Spot No.: F23

Protein name: Phosphoribulokinase

Peptide sequences:

K.ILVIEGLHPMFDER.V;R.VRDLLDFSIYLDISNEVK.F;K.HFSPVYLFDEGSTISWIPCGR.K;R.LDELIYVESHLSNISTR.F;K.IRDLYEQITSSK.T

Accession No.: Gohir.A12G204600.1.p

Mascot score: 449 **Sequence coverage %:**20

Calculated Mr: 45621 **Calculated *p*I:** 6.14

PFF Searched Score:

Ions score is -10*Log(P), where P is the probability that the observed match is a random event.
Individual ions scores > 34 indicate identity or extensive homology (p<0.05).
Protein scores are derived from ions scores as a non-probabilistic basis for ranking protein hits.

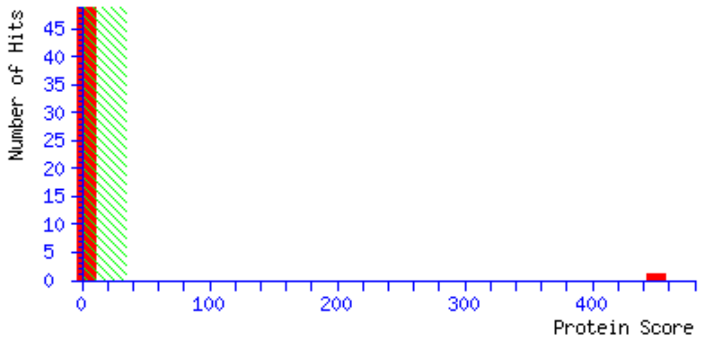

Matched peptide sequences: shown in Bold Red:

```
  1 MAVCSAYTTQ SLHSTCSIST PAKTHLGFHQ KQVVFYRRSS KRGGSGSSSG
 51 PCVITCSAGD SQTVVIGLAA DSGCGKSTFM RRLTSVFGGA AEPPKGGNPD
101 SNTLISDMTT VICLDDYHSL DRTGRKEKGV TALDPRANDF DLMYEQVKAI
151 KSGVAVDKPI YNHVTGLLDP PELIKPPKIL VIEGLHPMFD ERVRDLLDFS
201 IYLDISNEVK FAWKIQRDMA ERGHSLESIR ASIEARKPDF DAYIDPQKKY
251 ADAVIEVLPT QLIPDDNEGK VLRVRLIMKE GVKHFSPVYL FDEGSTISWI
301 PCGRKLTCSY PGIKFHYGPD TYFGNEVSIL EMDGQFDRLD ELIYVESHLS
351 NISTRFYGEV TQQMLKHADF PGSNNGTGLF QTIVGLKIRD LYEQITSSKT
401 AAPLEATKA
```

Spot No.: F24

Protein name: Phosphoribulokinase

Peptide sequences:

K.ILVIEGLHPMFDER.V;R.VRDLLDFSIYLDISNEVK.F;R.DLLDFSIYLDISNEVK.F;R.KPDFDAFIDPQK.Q;K.EGVEHFSPVYLFDEGSTISWIPCGR.K;K.FSYGPDTYFGHEVSVLEMDGQFDR.L

Accession No.: Gohir.D03G177400.1.p

Mascot score: 436 **Sequence coverage %:**22

Calculated Mr: 45591 **Calculated *p*I:** 5.96

PFF Searched Score:

Ions score is -10*Log(P), where P is the probability that the observed match is a random event.
Individual ions scores > 33 indicate identity or extensive homology (p<0.05).
Protein scores are derived from ions scores as a non-probabilistic basis for ranking protein hits.

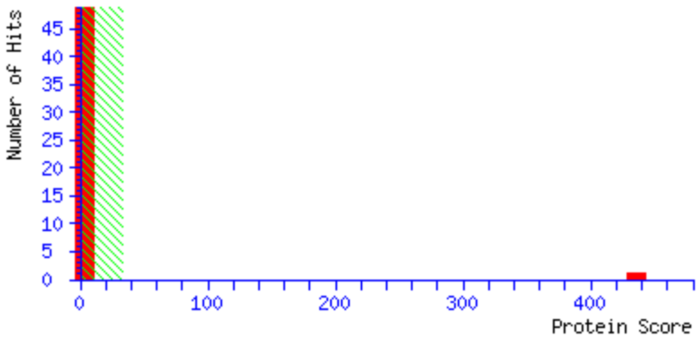

Matched peptide sequences: shown in Bold Red:

```
  1 MAVCPVYTTQ SLNSTCSIST PSKTHFSSHH NHLVFYRTSK RTSKRGGSSS
 51 CVITCSAGDS QTVVIGLAAD SGCGKSTFMR RLTSVFGGAA EPPKGGNPDS
101 NTLISDMTTV ICLDDYHSLD RTGRKEKGVT ALDPRANDFD LMYEQVKALK
151 SGIAVDKPIY NHVTGLLDPP ELIKPPKILV IEGLHPMFDE RVRDLLDFSI
201 YLDISNEVKF AWKIQRDMAE RGHSLESIKA SIEARKPDFD AFIDPQKQYA
251 DAVIEVLPTQ LIPDDNEGKV LRVRLIMKEG VEHFSPVYLF DEGSTISWIP
301 CGRKLTCSYP GIKFSYGPDT YFGHEVSVLE MDGQFDRLDE LIYVESHLSN
351 LSTKFYGEVT QQMLKHADFP GSNNGTGLFQ TIVGLKIRDL YEQITASKTA
401 APLQATKA
```

Spot No.: G1

Protein name: Phosphoribulokinase

Peptide sequences:

K.ILVIEGLHPMFDER.V;R.VRDLLDFSIYLDISNEVK.F;R.KPDFDAFIDPQK.Q;K.FSYGPDTYFGHEVSVLEMDGQFDR.L

Accession No.: Gohir.D03G177400.1.p

Mascot score: 234 **Sequence coverage %:** 16

Calculated Mr: 45591 **Calculated *p*I:** 5.96

PFF Searched Score:

Ions score is -10*Log(P), where P is the probability that the observed match is a random event.
Individual ions scores > 34 indicate identity or extensive homology (p<0.05).
Protein scores are derived from ions scores as a non-probabilistic basis for ranking protein hits.

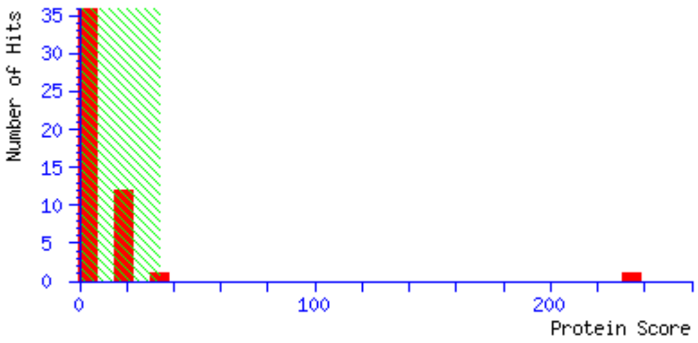

Matched peptide sequences: shown in Bold Red:

```
  1 MAVCPVYTTQ SLNSTCSIST PSKTHFSSHH NHLVFYRTSK RTSKRGGSSS
 51 CVITCSAGDS QTVVIGLAAD SGCGKSTFMR RLTSVFGGAA EPPKGGNPDS
101 NTLISDMTTV ICLDDYHSLD RTGRKEKGVT ALDPRANDFD LMYEQVKALK
151 SGIAVDKPIY NHVTGLLDPP ELIKPPKILV IEGLHPMFDE RVRDLLDFSI
201 YLDISNEVKF AWKIQRDMAE RGHSLESIKA SIEARKPDFD AFIDPQKQYA
251 DAVIEVLPTQ LIPDDNEGKV LRVRLIMKEG VEHFSPVYLF DEGSTISWIP
301 CGRKLTCSYP GIKFSYGPDT YFGHEVSVLE MDGQFDRLDE LIYVESHLSN
351 LSTKFYGEVT QQMLKHADFP GSNNGTGLFQ TIVGLKIRDL YEQITASKTA
401 APLQATKA
```

Spot No.: G3

Protein name: Malate dehydrogenase 1

Peptide sequences:

R.VLVTGAAGQIGYALVPMIAR.G;K.MELVDAAFPLLK.G;K.GVVATTDAVEACTGVNVAVMVGGFPR.K;R.ELVKDDAWLNGEFITTVQQR.G;K.LSSALSAASAACDHIR.D

Accession No.: Gohir.D02G045500.1.p

Mascot score: 288 **Sequence coverage %:**28

Calculated Mr: 35830 **Calculated *p*I:** 6.10

PFF Searched Score:

Ions score is -10*Log(P), where P is the probability that the observed match is a random event.
Individual ions scores > 34 indicate identity or extensive homology (p<0.05).
Protein scores are derived from ions scores as a non-probabilistic basis for ranking protein hits.

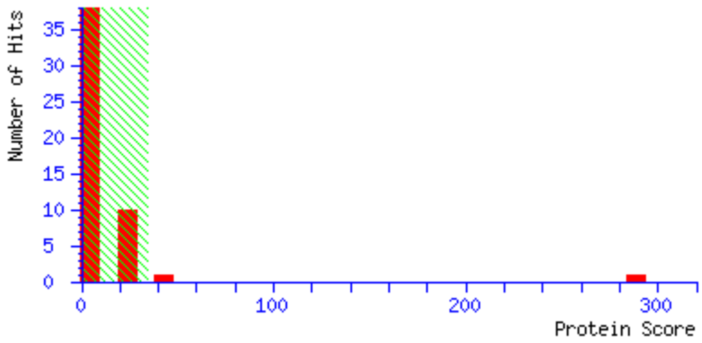

Matched peptide sequences: shown in Bold Red:

```
  1 MAKEPVRVLV TGAAGQIGYA LVPMIARGVM LGADQPVILH MLDIPPAAEA
 51 LNGVKMELVD AAFPLLKGVV ATTDAVEACT GVNVAVMVGG FPRKEGMERK
101 DVMSKNVSIY KSQASALEQH AAPNCKVLVV ANPANTNALI LKEFAPSIPA
151 KNITCLTRLD HNRALGQISE KLNVQVSDVK NVIIWGNHSS TQYPDVNHAT
201 VMTPSGEKPV RELVKDDAWL NGEFITTVQQ RGAAIIKARK LSSALSAASA
251 ACDHIRDWVL GTPEGTWVSM GVYSDGSYNA PAGVIYSFPV TCKNGEWTIV
301 QGLAIDEFSR KKLDLTGVEL TEEKELAYSC LS
```

Spot No.: G5

Protein name: Coproporphyrinogen III oxidase 1

Peptide sequences:

K.AGVNISVVYGVMPPEAYR.A;K.NPFAPTLHFNYR.Y;K.FDPSFYPR.F;R.YVEFNLVYDR.G;R.IESILVSLPLSAR.W

Accession No.: Gohir.A08G050600.1.p

Mascot score: 411 **Sequence coverage %:**20

Calculated Mr: 34176 **Calculated** ***p*****I:** 5.63

PFF Searched Score:

Ions score is -10*Log(P), where P is the probability that the observed match is a random event.
Individual ions scores > 35 indicate identity or extensive homology (p<0.05).
Protein scores are derived from ions scores as a non-probabilistic basis for ranking protein hits.

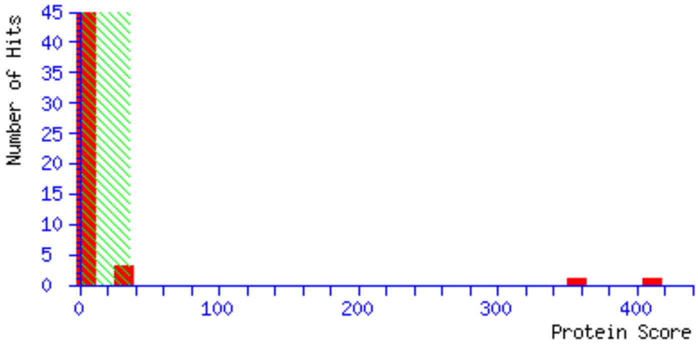

Matched peptide sequences: shown in Bold Red:

1 MILEAQESVC AALEAVDGAG KFKEDAWTRP GGGGGISRVL QDGAVFEK**AG**
51 **VNISVVYGVM PPEAYR**AAKA AADDQKPGPV PFFAAGISSV LHPK**NPFAPT**
101 **LHFNYR**YFET DAPKDTPGAP RQWWFGGGTD LTPAYIFEED VKHFHSIQKK
151 ACDK**FDPSFY PR**FKKWCDDY FYIKHRGERR GLGGIFFDDL NDYDQEMLLS
201 FATECANSVV PAYIPIIEKR KDTPFNESQK AWQQLRRGR**Y VEFNLVYDR**G
251 TTFGLKTGGR **IESILVSLPL SAR**WEYDHKP EEGSEEWKLL DACINPKEWI
301

Spot No.: G6

Protein name: Fructose-bisphosphate aldolase

Peptide sequences:

R.LASIGLENTEANR.Q;K.GLVPLPGSNNESWCQGLDGLSSR.T;R.TAAYYQQGAR.F;R.YAAISQDSGLVPIVEPEILLDGDHGIDR.T

Accession No.: Gohir.A13G030400.1.p

Mascot score: 327 **Sequence coverage %:**18

Calculated Mr: 43216 **Calculated *p*I:** 8.44

PFF Searched Score:

Protein score is -10*Log(P), where P is the probability that the observed match is a random event.
Protein scores greater than 62 are significant (p<0.05).
Protein scores are derived from ions scores as a non-probabilistic basis for ranking protein hits.

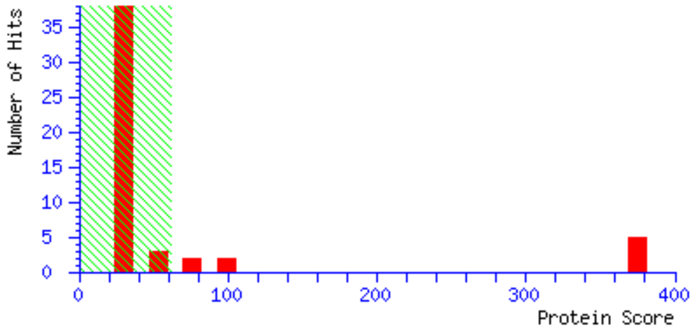

Matched peptide sequences: shown in Bold Red:

1 MASASATLLK SSPIIDKSEW IKGQNLRHPS VCFVRSHPTS AAFTVRASSY
51 ADELVKTAKT VASPGRGILA MDESNATCGK R**LASIGLENT** **EANR**QAYRTL
101 LVSAPGLGNY ISGAILFEET LYQSTIDGKK MVDVLVEQNI VPGIKVDK**GL**
151 **VPLPGSNNES** **WCQGLDGLSS** **RTAAYYQQGA** **R**FAKWRTVVS IPNGPSALAV
201 KEAAWGLAR**Y** **AAISQDSGLV** **PIVEPEILLD** **GDHGIDR**TFE VAQKVWAEVF
251 FYLAENNVMF EGILLKPSMV TPGAECKDKA TPQQVADYTL KLLHRRIPPA
301 VPGIMFLSGG QSEVEATLNL NAMNQSPNPW HVSFSYARAL QNTCLKTWGG
351 RPENVKAAQD TLLVRAKANS LAQLGKYTGE GESEEAKKGM FVKGYVY

Spot No.: G9

Protein name: 30S ribosomal protein, putative

Peptide sequences: R.AEEDAETVYGSIDLVSSILQR.K

Accession No.: Gohir.1Z057400.1.p

Mascot score: 202 **Sequence coverage %:**7

Calculated Mr: 32072 **Calculated *p*I:** 5.53

PFF Searched Score:

Ions score is -10*Log(P), where P is the probability that the observed match is a random event.
Individual ions scores > 34 indicate identity or extensive homology (p<0.05).
Protein scores are derived from ions scores as a non-probabilistic basis for ranking protein hits.

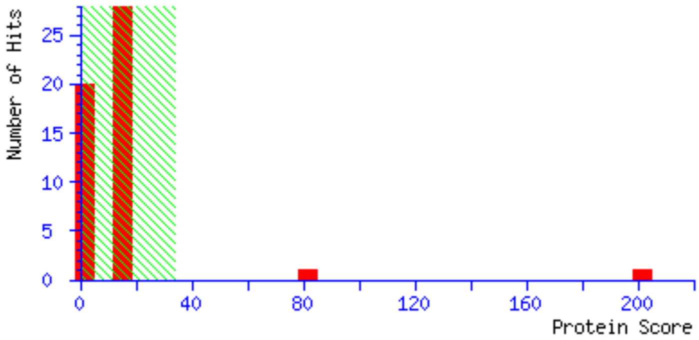

Matched peptide sequences: shown in Bold Red:

```
  1 MATTLLASSQ TSFHHPLSVS DSSFSSPSSS SSSVSMFTLT KPRIPSLPSY
 51 KSSFFNPIGN YFKCVEMEPR KIRNSSLTVR MSWDGPLASV KLIIQGKSLE
101 LTDTVKQHVE EKVTLFTKKH GVVRAEEDAE TVYGSIDLVS SILQRKLRKI
151 KEKESDRGRH MKGFSRSKVR EPVAVVVDDD AEAVPEQVVA DDDVAAVPEQ
201 EDDSFIDEIV RTKYFEMPPL TVSEAVEQLE NVDHDFYGFR NEETGEINII
251 YKRKAGGYGL IIPKGNGKAE KLEPLVVESA KEHSYVE
```

Spot No.: G11

Protein name: Ferredoxin-NADP(+)-oxidoreductase 2

Peptide sequences:

K.ITGDDAPGETWHMVFSTEGEVPYR.E;K.DPNATIIMLATGTG IAPFR.S

Accession No.: Gohir.A05G186100.1.p

Mascot score: 210 **Sequence coverage %:**11

Calculated Mr: 40678 **Calculated *p*I:** 8.36

PFF Searched Score:

Ions score is -10*Log(P), where P is the probability that the observed match is a random event.
Individual ions scores > 34 indicate identity or extensive homology (p<0.05).
Protein scores are derived from ions scores as a non-probabilistic basis for ranking protein hits.

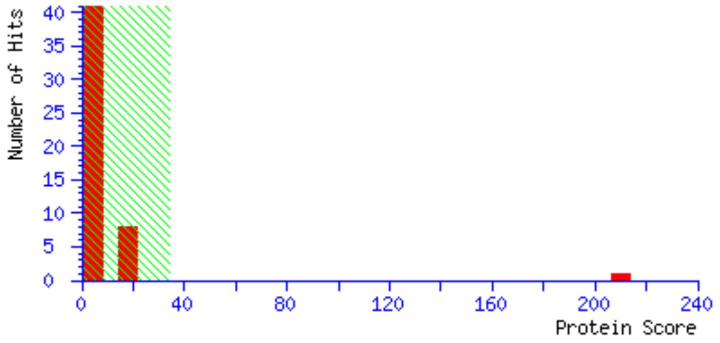

Matched peptide sequences: shown in Bold Red:

```
  1 MAMTVNAAVS LPSSKSSTLS FKNSVTVPER INFNKSVLYP KNVSLGGNVV
 51 SIRAQVTTEA PAKAPKISKK DDEGIVVNKF KPKEPYIGKC LLNTKITGDD
101 APGETWHMVF STEGEVPYRE GQSIGVIPDG IDKNGKPHKL RLYSIASSAL
151 GDFGNSQTVS LCVKRLVYTN EQGEIVKGVC SNFLCDLKPG AEVKITGPVG
201 KEMLLPKDPN ATIIMLATGT GIAPFRSFLW KMFFEKHDDY KFNGLAWLFL
251 GVPTSSSLLY PEEFEKMKEK APNNLRVDYA ISREQTNEQG EKMYIQTRMA
301 QYAEELWELL KKDNTYVYMC GLKGMEKGID DIMTSLAAKD GIDWIEYKRQ
351 MKKGEQWNVE VY
```

Spot No.: G15

Protein name: Photosystem II subunit O-2

Peptide sequences:

K.RLTYDEIQSK.T;K.FEEKDGIDYAAVTVQLPGGER.V;K.DGIDYAAVTVQLPGGER.V;R.VPFLFTIK.Q;R.GGSTGYDNAVALPAGGR.G;K.SKPETGEVIGVFESLQPSDTDLGAK.T

Accession No.: Gohir.D11G194600.1.p

Mascot score: 623 **Sequence coverage %:**24

Calculated Mr: 35293 **Calculated *p*I:** 5.37

PFF Searched Score:

Ions score is -10*Log(P), where P is the probability that the observed match is a random event.
Individual ions scores > 34 indicate identity or extensive homology (p<0.05).
Protein scores are derived from ions scores as a non-probabilistic basis for ranking protein hits.

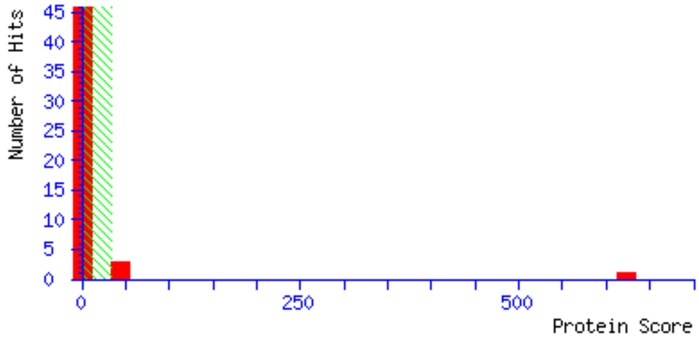

Matched peptide sequences: shown in Bold Red:

```
  1 MSVSLQAAAT LMQPIKVAAP SRTNVLLRSS QSVSKAFGLE PVGARLTCSL
 51 QTDLKDLAQT CVDATKLAGF ALATSALVVS GASAEGVPKR LTYDEIQSKT
101 YMEVKGTGTA NQCPTIDGGV DSFAFKPGKY YAKKFCLEPT SFTVKAEGVN
151 KNAPPEFQNT KLMTRLTYTL DEIEGPFEVS TDGTVKFEEK DGIDYAAVTV
201 QLPGGERVPF LFTIKQLVAS GKPDSFGGDF LVPSYRGSSF LDPKGRGGST
251 GYDNAVALPA GGRGDEEELA KENNKSAASS SGKITLSVTK SKPETGEVIG
301 VFESLQPSDT DLGAKTPKDV KITGVWYAQL DS
```

Spot No.: G16

Protein name: 2-phosphoglycolate phosphatase 1

Peptide sequences:

K.LIDGVPETLDMLR.S;K.DVGAVVVGFDR.Y;K.VQYGTLCIR.E;R.ENPGCLFIATNR.D

Accession No.: Gohir.A02G108400.1.p

Mascot score: 142 **Sequence coverage %:**12

Calculated Mr: 41067 **Calculated *p*I:** 8.45

PFF Searched Score:

Ions score is -10*Log(P), where P is the probability that the observed match is a random event.
Individual ions scores > 35 indicate identity or extensive homology (p<0.05).
Protein scores are derived from ions scores as a non-probabilistic basis for ranking protein hits.

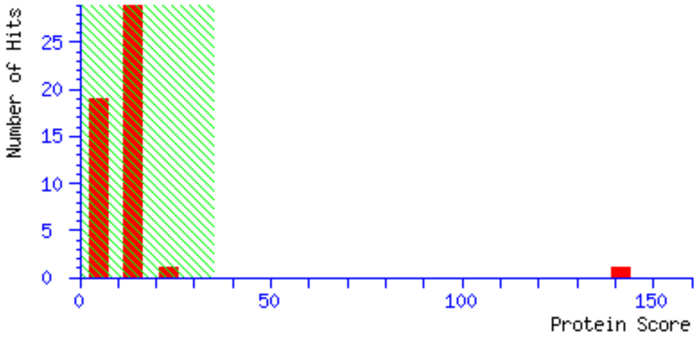

Matched peptide sequences: shown in Bold Red:

1 MATKAAVSVA ASVTAASSSS SKFVINTPHR FLCLKRLSSF SSFSASALAI
51 KCTSISNCSP NKSARSMEGF TTRTKASAQP LENADELIAS VETFIFDCDG
101 VIWKGDK**LID GVPETLDMLR** SKGKRLVFVT NNSTKSRKQY GKKFETLGLN
151 VNEEEIFASS FAAAAYLKSI NFPKDKKVYV IGEDGILKEL ELAGFQYLGG
201 PEDGGKKIEL KPGFLMEHDK **DVGAVVVGFD R**YFNYYK**VQY GTLCIRENPG**
251 **CLFIATNR**DA VTHLTDAQEW AGGGSMVGAI CGSTQREPLV VGKPSTFMMD
301 YLANKFGILK SQICMVGDRL DTDILFGQNG GCKTLLVLSG VTNLSMLQSP
351 SNSIQPDFYT NKISDFLSIK TATV

Spot No.: G17

Protein name: Oxygen-evolving enhancer protein 1

Peptide sequences:

K.FEEKDGIDYAAVTVQLPGGER.V;K.DGIDYAAVTVQLPGGER.V;R.GGSTGYDNAVALPAGGR.G

Accession No.: Gohir.A11G188100.1.p

Mascot score: 246 **Sequence coverage %:**11

Calculated Mr: 35233 **Calculated *p*I:** 5.84

PFF Searched Score:

Ions score is -10*Log(P), where P is the probability that the observed match is a random event.
Individual ions scores > 34 indicate identity or extensive homology (p<0.05).
Protein scores are derived from ions scores as a non-probabilistic basis for ranking protein hits.

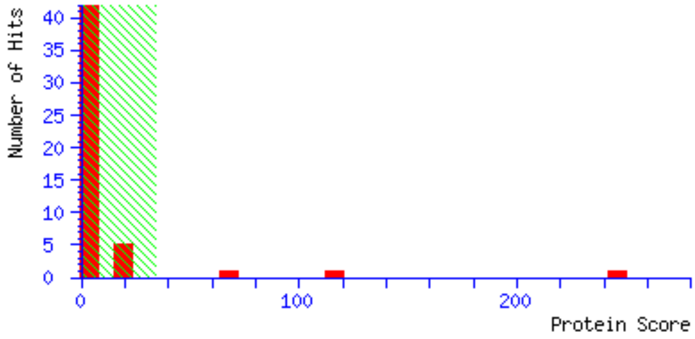

Matched peptide sequences: shown in Bold Red:

```
  1 MAVSLQAAAT LMQPIKVAAP SRTNVPLRSS QSVSKAFGLE PVGARLTCSL
 51 QTDLKDLALK CVDATKLAGF ALATSALVVS GASAEGVSKR LTYAEIQSKT
101 YMEVKGTGTA NQCPTIDGGV DSFAFKPGKY YAKKFCLEPT SFTVKAEGVN
151 KNAPPEFQNT KLMTRLTYTL DEIEGPFEVS TDGTLKFEEK DGIDYAAVTV
201 QLPGGERVPF LFTIKQLVAS GKPDSFGGDF LVPSYRGSSF LDPKGRGGST
251 GYDNAVALPA GGRGDEEELA KENNKSAASS SGKITLSVTK SKPETGEVIG
301 VFESLQPSDT DLGAKTPKDV KITGVWYAQL DS
```

Spot No.: G18

Protein name: Oxygen-evolving enhancer protein 1

Peptide sequences:

K.FEEKDGIDYAAVTVQLPGGER.V;K.DGIDYAAVTVQLPGGER.V;R.VPFLFTIK.Q;R.GGSTGYDNAVALPAGGR.G;K.SKPETGEVIGVFESLQPSDTDLGAK.T

Accession No.: Gohir.A11G188100.1.p

Mascot score: 343 **Sequence coverage %:**21

Calculated Mr: 35233 **Calculated *p*I:** 5.84

PFF Searched Score:

Ions score is -10*Log(P), where P is the probability that the observed match is a random event.
Individual ions scores > 34 indicate identity or extensive homology (p<0.05).
Protein scores are derived from ions scores as a non-probabilistic basis for ranking protein hits.

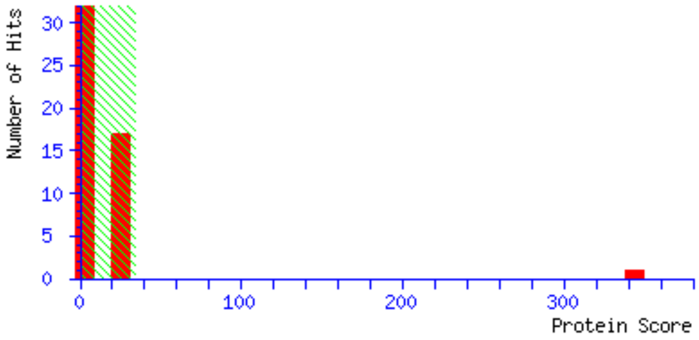

Matched peptide sequences: shown in Bold Red:

```
  1 MAVSLQAAAT LMQPIKVAAP SRTNVPLRSS QSVSKAFGLE PVGARLTCSL
 51 QTDLKDLALK CVDATKLAGF ALATSALVVS GASAEGVSKR LTYAEIQSKT
101 YMEVKGTGTA NQCPTIDGGV DSFAFKPGKY YAKKFCLEPT SFTVKAEGVN
151 KNAPPEFQNT KLMTRLTYTL DEIEGPFEVS TDGTLKFEEK DGIDYAAVTV
201 QLPGGERVPF LFTIKQLVAS GKPDSFGGDF LVPSYRGSSF LDPKGRGGST
251 GYDNAVALPA GGRGDEEELA KENNKSAASS SGKITLSVTK SKPETGEVIG
301 VFESLQPSDT DLGAKTPKDV KITGVWYAQL DS
```

Spot No.: G19

Protein name: Glycine-rich RNA-binding protein 3

Peptide sequences:

K.LFVGGISYQTDDQGLR.E;R.GFGFVTYTSSKDASSALQALDGQTLHGR.Q

Accession No.: Gohir.D07G072800.1

Mascot score: 90 **Sequence coverage %:**16

Calculated Mr: 27996 **Calculated *p*I:** 4.77

PFF Searched Score:

Ions score is -10*Log(P), where P is the probability that the observed match is a random event.
Individual ions scores > 35 indicate identity or extensive homology (p<0.05).
Protein scores are derived from ions scores as a non-probabilistic basis for ranking protein hits.

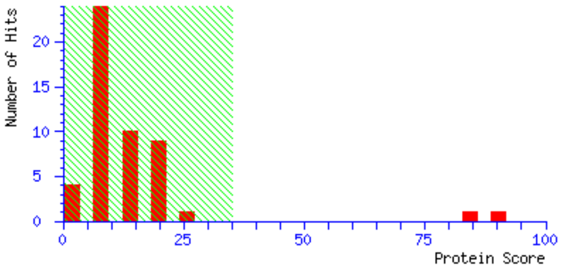

Matched peptide sequences: shown in Bold Red:

```
  1 MAFLSKIGNM LRQTSTTQIN AQLSASSPSL FQVFRCMSNA PSSKLFVGGI
 51 SYQTDDQGLR EAFSKYGEVI EARIIVDRET GRSRGFGFVT YTSSKDASSA
101 LQALDGQTLH GRQVRVNYAT ERAPRNFGGG GYGGGGYGGG GYGGGGYGGG
151 GYGGGDYGRN AAPTSGNYGG NVGYGGSGNY GGQGSHGGFG QSDATNYESG
201 SFSVAGGGGG IDGVGTGGSA GFGSPNMGID SVDKFSSTED GFKEEEAFDS
251 NEPMNENFRD EDDENGSDFA KRA
```

Spot No.: G21

Protein name: Ribose 5-phosphate isomerase, type A protein

Peptide sequences:

R.TQEQAASLNIPLSTLDLHPR.I;R.IDLAIDGADEVDPNLDLVK.G

Accession No.: Gohir.A11G253800.1.p

Mascot score: 113 **Sequence coverage %:** 13

Calculated Mr: 29906 **Calculated *p*I:** 6.44

PFF Searched Score:

Ions score is -10*Log(P), where P is the probability that the observed match is a random event.
Individual ions scores > 32 indicate identity or extensive homology (p<0.05).
Protein scores are derived from ions scores as a non-probabilistic basis for ranking protein hits.

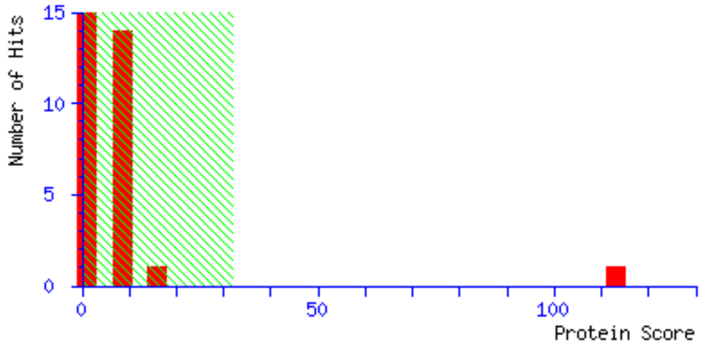

Matched peptide sequences: shown in Bold Red:

```
  1 MASLSLNLSS LHHTSINPLI LRCTPLNLRT PSQKPFSIRS QAAPVLSQDD
 51 LKKLAADKAV ESVKSGMILG LGTGSTAAFV VDKIGQLLST GQLSNIVGIP
101 TSKRTQEQAA SLNIPLSTLD LHPRIDLAID GADEVDPNLD LVKGRGGALL
151 REKIVEAASS SFIVVADESK LVSGLGGSGL AMPVEVVQFC WKYNLIRLQG
201 LFKELGCEAK LRLVGDGSEK PYVTDNGNYI VDLYFKNPIK DGFGAGKEIS
251 ALEGVVEHGL FLGMATSVII AGKTGIEVMT K
```

Spot No.: G24

Protein name: Photosystem I light harvesting complex gene 3

Peptide sequences:

K.AGLIPAETALPWFR.T;K.YLGGSGEPAYPGGPLFNPLGFGKD EK.S

Accession No.: Gohir.A11G146500.1.p

Mascot score: 152 **Sequence coverage %:** 8.86

Calculated Mr: 29458 **Calculated *p*I:** 14

PFF Searched Score:

Ions score is -10*Log(P), where P is the probability that the observed match is a random event.
Individual ions scores > 35 indicate identity or extensive homology (p<0.05).
Protein scores are derived from ions scores as a non-probabilistic basis for ranking protein hits.

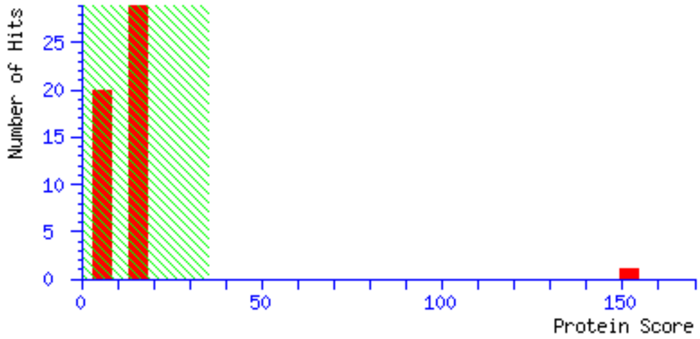

Matched peptide sequences: shown in Bold Red:

```
  1 MATQALVSSS LTYSVETARQ ILGAKRQIGS SRKGSFVVKA ASTPPVKQGA
 51 DRPLWFASKQ SLSYLDGSLP GDYGFDPLGL SDPEGPGGFI EPKWLAYGEI
101 INGRYAMLGA VGAIAPEILG KAGLIPAETA LPWFRTGVIP PAGTYNYWAD
151 PYTLFVFEMA LMGFAEHRRF QDWAKPGSMG KQYFLGFEKY LGGSGEPAYP
201 GGPLFNPLGF GKDEKSLKDL KLKEVKNGRL AMLAILGYFI QGLVTGVGPY
251 QNLLDHLADP FNNNVLTNLK FH
```

Spot No.: H1

Protein name:

Peptide sequences:

Accession No.:

Mascot score: **Sequence coverage %:**

Calculated Mr: **Calculated *p*I:**

PFF Searched Score:

Ions score is -10*Log(P), where P is the probability that the observed match is a random event.
Individual ions scores > 34 indicate identity or extensive homology (p<0.05).
Protein scores are derived from ions scores as a non-probabilistic basis for ranking protein hits.

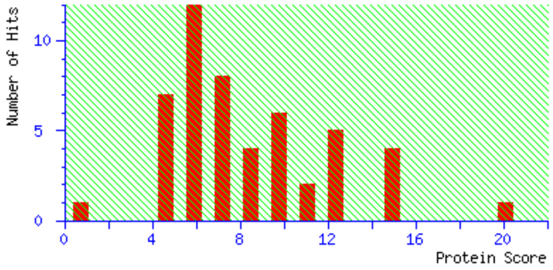

Matched peptide sequences: shown in Bold Red:

Spot No.: H2

Protein name: Oxygen-evolving enhancer protein 2-1

Peptide sequences:

K.EVEFPGQVLR.Y;K.SITDYGSPEEFLSSVDYLLGK.Q;K.TDSEGGFDSGAVATANILESSSSTVGGKPYYFLSVLTR.T;K.PYYFLSVLTR.T

Accession No.: Gohir.D04G034900.1.p

Mascot score: 352 **Sequence coverage %:** 26

Calculated Mr: 28189 **Calculated *p*I:** 7.67

PFF Searched Score:

Ions score is -10*Log(P), where P is the probability that the observed match is a random event.
Individual ions scores > 34 indicate identity or extensive homology (p<0.05).
Protein scores are derived from ions scores as a non-probabilistic basis for ranking protein hits.

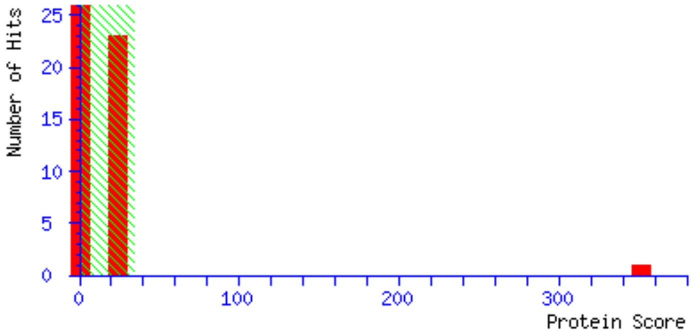

Matched peptide sequences: shown in Bold Red:

1 MASAACFLHH HALTTNGRSS SLPPSLRQVS NMKPNQQVVC KAQKQEDDGS
51 LVSRRLALTV LIGAAAVGSK VSPADAAYGE AANVFGKPKT DTEFMPYNGD
101 GFKLSIPSKW NPSK**EVEFPG** **QVLR**YEDNFD TTSNLSVMIT PTDKK**SITDY**
151 **GSPEEFLSSV** **DYLLGK**QAYF GK**TDSEGGFD** **SGAVATANIL** **ESSSSTVGGK**
201 **PYYFLSVLTR** TADGDEGGKH QLITATVNNG KLYICKAQAG DKRWFKGARK
251 FVESAASSFS VA

Spot No.: H3

Protein name: Photosystem II subunit P-1

Peptide sequences:

K.EVEFPGQVLR.Y;K.SITDYGSPEEFLSSVDYLLGK.Q;K.TDAEGGFDSGAVATANILESSSSTVGGKPYYFLSVLTR.T;K.PYYFLSVLTR.T

Accession No.: Gohir.A05G380700.1.p

Mascot score: 313 **Sequence coverage %:** 26
Calculated Mr: 28109 **Calculated *p*I:** 7.67

PFF Searched Score:

Ions score is -10*Log(P), where P is the probability that the observed match is a random event.
Individual ions scores > 34 indicate identity or extensive homology (p<0.05).
Protein scores are derived from ions scores as a non-probabilistic basis for ranking protein hits.

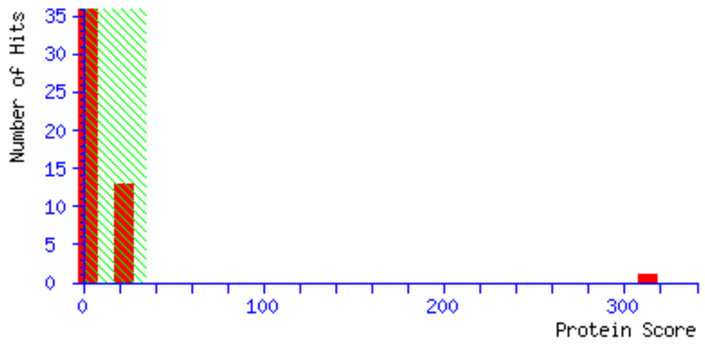

Matched peptide sequences: shown in Bold Red:

```
  1 MASAACFLHH HALTTNGRSS SLSPSLRQVS NMKPTQQVVC KAQKQEDDGS
 51 LVSRRLALTV LIGAAAVGSK VSPADAAYGE AANVFGKPKT DTDFMPYSGD
101 GFKLSIPSKW NPSKEVEFPG QVLRYEDNFD TTSNLSVMIT PTDKKSITDY
151 GSPEEFLSSV DYLLGKQAYF GKTDAEGGFD SGAVATANIL ESSSSTVGGK
201 PYYFLSVLTR TADGDEGGKH QLITATVNNG KLYICKAQAG DKRWFKGARK
251 FVESAASSFS VA
```

Spot No.: H5

Protein name: Ribulose-bisphosphate carboxylases

Peptide sequences:

K.LTYYTPEYEVK.D;K.DTDILAAFR.V;K.TFKGPPHGIQVER.D

Accession No.: Gohir.A03G084800.1

Mascot score: 165 **Sequence coverage %:** 8

Calculated Mr: 46433 **Calculated *p*I:** 5.99

PFF Searched Score:

Ions score is -10*Log(P), where P is the probability that the observed match is a random event.
Individual ions scores > 35 indicate identity or extensive homology (p<0.05).
Protein scores are derived from ions scores as a non-probabilistic basis for ranking protein hits.

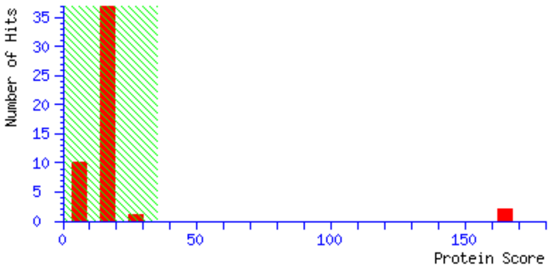

Matched peptide sequences: shown in Bold Red:

1 MSCREGLMSP QTETKASVGF KAGVKEYK**LT YYTPEYEVKD TDILAAFR**VT
51 PQPGVPPEEA GAAVAAESST GTWTTMWTDG LTSLDRYEGR CYDIEPVSGE
101 EDQYICYVAY PLDLFEEGSV TNMFTSIVGN VFGFKALRAL RLEDLRVPTA
151 YIK**TFKGPPH GIQVER**DKLN KYGRPLLGCT IKPKLDDENV NSQPFMRWRD
201 RFLFCAKAIF KSQAETGEIK GHYLNATAGT CEEMIKRAMC ARELGVPIVM
251 HDYLTDNGLL LHIHRAMHAV IDRQKNHGMH FRVLAKALRM SGGDHIHAGT
301 VVDRSRGIYF TQDWVSMPGV LPVASGGIHV WHMPALIEIF GDDSVLQFAN
351 RVALEACVQA RNEGRDLARE GNEIIREASK WSPELAVACE VWKAIKFEFD
401 AVDKLDKVEK

Spot No.: H10

Protein name: Photosynthetic electron transfer C

Peptide sequences:

K.DAIGNDVIAEEWLK.T;K.FICPCHGSQYNDQGR.V;R.GPAPLSLALAHAGVEDGK.V;K.VVFVPWVETDFR.T

Accession No.: Gohir.D02G184300.1.p

Mascot score: 256 **Sequence coverage %:** 25

Calculated Mr: 24700 **Calculated *p*I:** 8.48

PFF Searched Score:

Ions score is -10*Log(P), where P is the probability that the observed match is a random event.
Individual ions scores > 34 indicate identity or extensive homology (p<0.05).
Protein scores are derived from ions scores as a non-probabilistic basis for ranking protein hits.

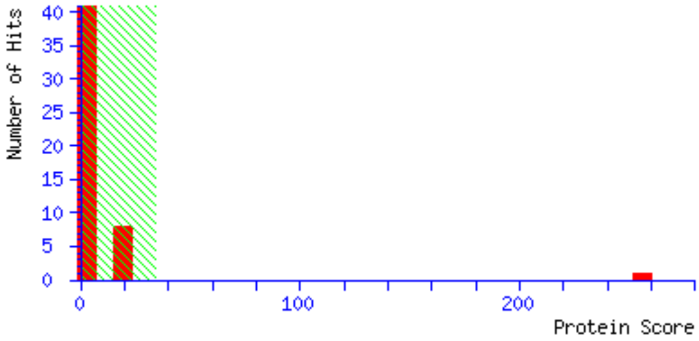

Matched peptide sequences: shown in Bold Red:

```
  1 MASSTLSPAA PWQLCSGKSG MFCPSQAFLV KPTRTHMVKN EKGMRITCQA
 51 TSIPADRVPD MGKRQLMNLL LLGAISLPSG FMLVPYAAFF VPSGGRGTGG
101 GTVAKDAIGN DVIAEEWLKT HGPGDRTLTQ GLKGDPTYLV VEKDRTLATY
151 GINAVCTHLG CVVPWNQAEN KFICPCHGSQ YNDQGRVVRG PAPLSLALAH
201 AGVEDGKVVF VPWVETDFRT GDGPWWS
```

Spot No.: **H15**

Protein name: **Nucleoside diphosphate kinase 2**

Peptide sequences:

Accession No.: **Gohir.A09G245300.1**

Mascot score: **Sequence coverage %:**

Calculated Mr: **Calculated *p*I:**

PFF Searched Score:

Ions score is -10*Log(P), where P is the probability that the observed match is a random event.
Individual ions scores > 35 indicate identity or extensive homology (p<0.05).
Protein scores are derived from ions scores as a non-probabilistic basis for ranking protein hits.

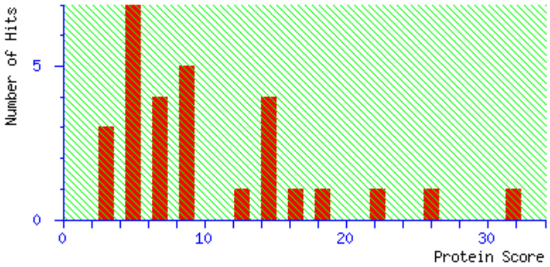

Matched peptide sequences: shown in Bold Red:

Spot No.: **H19**

Protein name: **Nucleoside diphosphate kinase family protein**

Peptide sequences:

R.GLVGEIIGR.F;K.IIGATNPAESAPGTIR.G;R.GDFAIDIGR.N

Accession No.: **Gohir.D08G160400.1.p**

Mascot score: **106** **Sequence coverage %:** **22**

Calculated Mr: **16400** **Calculated *p*I:** **6.30**

PFF Searched Score:

Ions score is -10*Log(P), where P is the probability that the observed match is a random event.
Individual ions scores > 34 indicate identity or extensive homology (p<0.05).
Protein scores are derived from ions scores as a non-probabilistic basis for ranking protein hits.

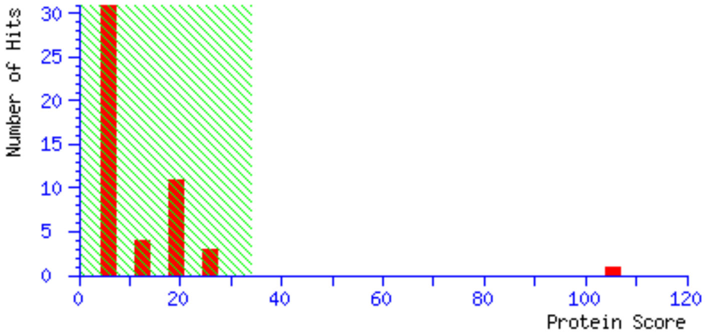

Matched peptide sequences: shown in Bold Red:

```
  1 MEQTFIMIKP DGVQRGLVGE IIGRFEKKGF YLKGLKLITV DQSFAEKHYA
 51 DLSAKPFFNG LIEYIISGPV VAMIWEGKNV VTTGRKIIGA TNPAESAPGT
101 IRGDFAIDIG RNVIHGSDSV ESAKKEIALW FPESPVNWQS SVHPWIYE
```
